# *Diuqin lechiguanae* gen. et sp. nov., a new unenlagiine (Theropoda: Paraves) from the Bajo de la Carpa Formation (Neuquén Group, Upper Cretaceous) of Neuquén Province, Patagonia, Argentina

**DOI:** 10.1186/s12862-024-02247-w

**Published:** 2024-06-14

**Authors:** Juan D. Porfiri, Mattia A. Baiano, Domenica D. dos Santos, Federico A. Gianechini, Michael Pittman, Matthew C. Lamanna

**Affiliations:** 1 Museo de Ciencias Naturales, Secretaría de Extensión, Universidad Nacional del Comahue, Buenos Aires, Neuquén, 1400 Argentina; 2 Cátedras de Paleontología y Reptiles Mesozoicos, Facultad de Ingeniería, Universidad Nacional del Comahue, Buenos Aires, Neuquén, 1400 Argentina; 3 Museo del Desierto Patagónico de Añelo, Calle 1 Familia Chávez y Calle 6 Auca Mahuida, Añelo, Neuquén Argentina; 4 School of Life Sciences, The Chinese University of Hong Kong, Shatin, Hong Kong SAR China; 5 CONICET-Área Laboratorio e Investigación, Museo Municipal “Ernesto Bachmann,” Dr. Natali s/n, Villa El Chocón, Neuquén, Argentina; 6 Universidad Nacional de Río Negro, Isidro Lobo 516, General Roca, Río Negro, 8332 Argentina; 7 Instituto Multidisciplinario de Investigaciones Biológicas de San Luis (CONICET-Universidad Nacional de San Luis), Ejército de Los Andes 950, San Luis, Argentina; 8 Section of Vertebrate Paleontology, Carnegie Museum of Natural History, 4400 Forbes Avenue, Pittsburgh, PA 15213 USA

**Keywords:** Unenlagiinae, Dromaeosauridae, Cretaceous, Bajo de la Carpa Formation, Patagonia, South America, Biostratigraphy, Phylogeny, Paleoecology, Megaraptoridae

## Abstract

**Background:**

Unenlagiine paravians are among the most relevant Gondwanan theropod dinosaur clades for understanding the origin of birds, yet their fossil record remains incomplete, with most taxa being represented by fragmentary material and/or separated by lengthy temporal gaps, frustrating attempts to characterize unenlagiine evolution.

**Results and conclusions:**

Here we describe *Diuqin lechiguanae* gen. et sp. nov., a new unenlagiine taxon from the Upper Cretaceous (Santonian) Bajo de la Carpa Formation of the Neuquén Basin of Neuquén Province in northern Patagonia, Argentina that fills a substantial stratigraphic gap in the fossil record of these theropods. Although known only from a very incomplete postcranial skeleton, the preserved bones of *Diuqin* differ from corresponding elements in other unenlagiines, justifying the erection of the new taxon. Moreover, in several morphological aspects, the humerus of *Diuqin* appears intermediate between those of geologically older unenlagiines from the Neuquén Basin (e.g., *Unenlagia* spp. from the Turonian–Coniacian Portezuelo Formation) and that of the stratigraphically younger, larger-bodied *Austroraptor cabazai* from the Campanian–Maastrichtian Allen Formation. Consequently, the morphology of the new taxon appears to indicate a transitional stage in unenlagiine evolution. Phylogenetic analysis recovers *Diuqin* as a paravian with multiple plausible systematic positions, but the strongest affinity is with Unenlagiinae. The humerus of the new form exhibits subcircular punctures near its distal end that are interpreted as feeding traces most likely left by a conical-toothed crocodyliform, mammal, or theropod, the latter potentially corresponding to a megaraptorid or another unenlagiine individual. Thus, in addition to filling important morphological and temporal gaps in unenlagiine evolutionary history, the new taxon also offers information relating to the paleoecology of these theropods.

**Supplementary Information:**

The online version contains supplementary material available at 10.1186/s12862-024-02247-w.

## Background

Unenlagiines are Gondwanan (Southern Hemisphere) predatory dinosaurs that are nested within Paraves, the clade that includes birds and their closest non-avian theropod relatives. The unenlagiine fossil record comes predominantly from Argentina, where the greatest number of specimens and the most complete skeletons have been found, although other materials at least tentatively assigned to Unenlagiinae have also been recovered from Brazil [1–3], Chile [4], Colombia [5], and Antarctica [6–8]. The small-bodied, potentially volant Madagascan theropod *Rahonavis ostromi* [9, 10] has also been frequently regarded as an unenlagiine, depending on the specific phylogenetic hypothesis employed [10–14]. Unenlagiines are most frequently interpreted as early diverging dromaeosaurids [2, 11, 12, 15], although other authors have instead regarded these theropods as a distinct paravian clade (Unenlagiidae; see [16–18]).

Unenlagiines are an important clade for understanding bird origins due to their close phylogenetic relationship with Avialae [11, 12, 14, 15, 17]. Unfortunately, however, most taxa are represented only by fragmentary fossils. At present, the definitive Argentinian unenlagiine record consists of six named species from Upper Cretaceous (Cenomanian–Maastrichtian) horizons in the Neuquén Basin of northern Patagonia: *Buitreraptor gonzalezorum* from the Cenomanian Candeleros Formation [11]; *Unenlagia comahuensis* [19], *Unenlagia paynemili* [20], *Neuquenraptor argentinus* [21], and *Pamparaptor micros* [22] from the Turonian–Coniacian Portezuelo Formation, and *Austroraptor cabazai* from the Campanian–Maastrichtian Allen Formation [23]. Some workers (e.g., [11, 12]) have suggested that *Neuquenraptor* may be a junior synonym of *Unenlagia* due to the strongly similar morphology of their few overlapping skeletal elements (all presently limited to the hind limb) as well as the close correspondence in geographic and stratigraphic provenance of the known specimens. Conversely, other authors (e.g., [18, 22]) have maintained the distinction of the two genera based on proposed anatomical differences between their hind limb bones. Given that the issue remains unresolved, we provisionally consider *Neuquenraptor* a valid taxon pending the discovery of additional material. Moreover, a paravian recently described from the Cenomanian–Turonian Huincul Formation, *Overoraptor chimentoi*, was recovered as the sister taxon of *Rahonavis*, though neither taxon was regarded as a member of Unenlagiinae by the describers of the former [24]. Other fragmentary possible Argentinian unenlagiine records have been reported from the Huincul [25] and the Coniacian–Santonian Plottier [26] formations of the Neuquén Basin, the Campanian–Maastrichtian Chorrillo Formation of the Austral-Magallanes Basin of southern Patagonia [27], and the Campanian or Maastrichtian Los Blanquitos Formation of Salta Province in northwestern Argentina (*Unquillosaurus ceibalii* [17, 28, 29]). Here we describe *Diuqin lechiguanae* gen. et sp. nov., the first unenlagiine taxon from the Santonian Bajo de la Carpa Formation of the Neuquén Basin. We show that *Diuqin* fills a crucial temporal and morphological gap in unenlagiine evolutionary history; moreover, we present new insights into the Bajo de la Carpa Formation paleoecosystem arising from tooth marks preserved on the humerus of the type specimen.

### Geological setting

#### Stratigraphic and paleoenvironmental context

**Fig. 1:**
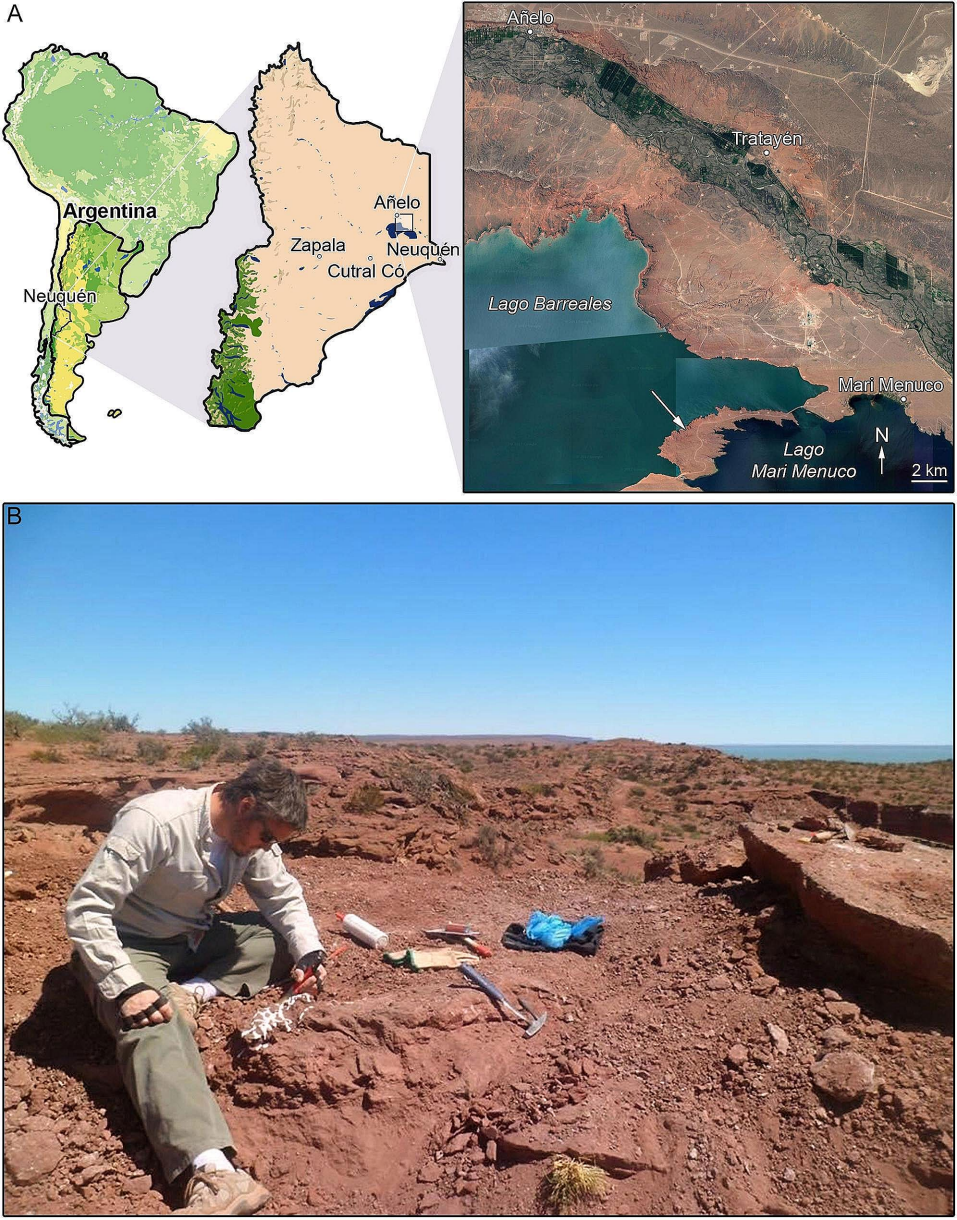
Type locality of *Diuqin lechiguanae* gen. et sp. nov., on the isthmus between Lago Barreales and Lago Mari Menuco, Neuquén Province, northern Patagonia, Argentina. **A**, map (modified from Porfiri et al. [35]; arrow in inset at right indicates fossil site). **B**, photograph of first author (JDP) excavating the holotype

The type and only known specimen of *Diuqin lechiguanae* was recovered from a red, uncemented, quartz-rich sandstone belonging to the Upper Cretaceous (Santonian) Bajo de la Carpa Formation exposed on the isthmus between Lago Barreales and Lago Mari Menuco in Neuquén Province of northern Patagonia, Argentina (Fig. 1). The Bajo de la Carpa Formation crops out in several areas of Neuquén and Río Negro provinces. In Neuquén, horizons of this formation are exposed on the campus of the Universidad Nacional del Comahue in the city of Neuquén, in the Tratayén area, and in the vicinity of Rincón de los Sauces (e.g., at the La Invernada locality); in Río Negro, these strata crop out in the Área Natural Protegida Municipal Paso Córdoba. The Bajo de la Carpa Formation is thought to have been deposited under a warm, semiarid paleoclimatic regime [30]. Most sediments of this formation were laid down in low-sinuosity fluvial paleoenvironments, though some of its strata exposed in and near the city of Neuquén are considered aeolian in origin [31]. Within the Upper Cretaceous, the age of the Bajo de la Carpa Formation is interpreted as Santonian [30–34]. See Garrido [30] and Rodríguez et al. [31] for further discussions of the geology and stratigraphic relationships of this unit.

#### Associated biota

The Bajo de la Carpa Formation has yielded fossils that collectively represent a diverse and important paleobiota [30, 32, 36–55]. Vertebrate remains are abundant and often well-preserved, and include those of snakes (*Dinilysia patagonica*), lizards (*Paleochelco occultato*), turtles (*Lomalatachelys neuquina*), crocodyliforms (*Notosuchus terrestris*, *Comahuesuchus brachybuccalis*, *Cynodontosuchus rothi*, *Wargosuchus australis*, *Neuquensuchus universitas*, *Microsuchus schilleri*, *Gasparinisuchus peirosauroides*, *Kinesuchus overoi*), indeterminate pterosaurs, ornithopods (*Mahuidacursor lipanglef*), titanosaurian sauropods (*Bonitasaura salgadoi*, *Overosaurus paradasorum*, *Rinconsaurus caudamirus*, *Traukutitan eocaudata*), non-avian theropods (*Velocisaurus unicus*, *Viavenator exxoni*, *Llukalkan aliocranianus*, *Tratayenia rosalesi*, *Alvarezsaurus calvoi*, *Achillesaurus manazzonei*), and birds (*Neuquenornis volans*, *Patagopteryx deferrariisi*, numerous nests and eggs).

#### List of institutional abbreviations

AMNH, American Museum of Natural History, New York, New York, USA. FMNH, Field Museum of Natural History, Chicago, Illinois, USA. MAU, Museo Municipal Argentino Urquiza, Rincón de los Sauces, Neuquén, Argentina. MCF-PVPH, Museo Carmen Funes, Plaza Huincul, Neuquén, Argentina. MML, Museo Municipal de Lamarque, Lamarque, Río Negro, Argentina. MPCA, Museo Provincial Carlos Ameghino, Cipolletti, Río Negro, Argentina. MPCN-PV, Museo Patagónico de Ciencias Naturales, General Roca, Río Negro, Argentina. MUCPv, Museo de Ciencias Naturales, Universidad Nacional del Comahue, Neuquén, Neuquén, Argentina. NHMUK, The Natural History Museum, London, United Kingdom. UA, Université d’Antananarivo, Antananarivo, Madagascar.

## Materials and methods

The holotype of the new unenlagiine taxon (MUCPv 1401) consists of the posteriormost sacral vertebral neural arch (MUCPv 1401/1), an anterior caudal vertebral neural arch (MUCPv 1401/2), the almost complete left humerus lacking the proximal end (MUCPv 1401/4), and four indeterminate bone fragments (at least two of which may correspond to vertebral fragments). The specimen was recovered from a stratum of the Bajo de la Carpa Formation by the first author (JDP) and his team at the Museo de Ciencias Naturales of the Universidad Nacional del Comahue from the isthmus between Lago Barreales and Lago Mari Menuco in Neuquén Province of northern Patagonia, Argentina, an area the museum is permitted to excavate fossils from under its terms of operation (Fig. 1). Following local law, the discovery was then communicated to the Dirección General de Patrimonio Cultural (Subsecretaría de Cultura de la Provincia de Neuquén) by JDP. The fossil was then prepared at the Museo de Ciencias Naturales of the Universidad Nacional del Comahue.

The holotype is considered to belong to a single unenlagiine individual due to: (1) the morphological similarities of its bones (mainly the humerus and posteriormost sacral vertebra) to those of other unenlagiine specimens; (2) the close physical proximity of the elements to one another (recovered from 1 m^2^); (3) the relative sizes of the bones, all of which are consistent with a single paravian individual; and (4) their similar color and preservational qualities. Moreover, the only other fossil discovered in reasonably close proximity is a tooth that pertains to what was almost certainly a much larger-bodied theropod taxon (a megaraptorid tetanuran, see below).

The following Patagonian Late Cretaceous unenlagiine taxa and specimens were examined firsthand during the course of this study: *Austroraptor cabazai* (MML 195 [holotype] and MML 220 [referred specimen]); *Buitreraptor gonzalezorum* (MPCA 245 [holotype] and MPCA 238, MPCA 471, and MPCA 478 [referred specimens]); *Neuquenraptor argentinus* (MCF-PVPH 77 [holotype]); *Pamparaptor micros* (MUCPv 1163 [holotype]); *Unenlagia comahuensis* (MCF-PVPH 78 [holotype]); *Unenlagia paynemili* (MUCPv 349 [holotype] and MUCPv 343, MUCPv 409, MUCPv 415, MUCPv 416, and MUCPv 1066 [referred specimens]). Morphological and/or taphonomic observations were supplemented using the following literature sources for the Gondwanan Late Cretaceous paravian taxa in question: *Austroraptor* [23, 56, 57]; *Buitreraptor* [11, 15, 18, 58–62]; *Imperobator antarcticus* [6, 7]; *Neuquenraptor* [21, 63]; *Overoraptor chimentoi* [24]; *Pamparaptor* [22, 57]; *Rahonavis ostromi* [9, 10]; *U*. *comahuensis* [19, 57, 64–66]; *U*. *paynemili* [20, 57]; *Ypupiara lopai* [2].

The specimen was diagnosed by experts specializing in a range of theropod dinosaurs, including unenlagiines (FAG), using both autapomorphies and a unique combination of characters. When the specimen was assigned to Unenlagiinae, additional advice was sought from a second unenlagiine expert who was not part of the study (but thanked in the acknowledgments) to refine our original assignment.

### Nomenclature

The nomenclature of vertebral neural arch laminae follows Wilson [67, 68], whereas that of fossae follows Wilson et al. [69]. Furthermore, we employ the taxonomic scheme in which Unenlagiinae is considered to be a subclade of Dromaeosauridae, in accordance with most previous works [e.g., 11, 12, 70] and the results of our phylogenetic analysis (see below).

### Phylogenetic analysis

To evaluate the phylogenetic position of *Diuqin lechiguanae*, we carried out a cladistic analysis using the data matrix of Gianechini et al. [15], adding some of the modifications of Napoli et al. ([70]; i.e., with some characters rescored in selected taxa and six species [*Kuru kulla*, *Moros intrepidus*, *Shri devi*, *Suskityrannus hazelae*, *Timurlengia euotica*, and *Ypupiara lopai*] added); this dataset is itself a modified version of the Theropod Working Group (TWiG) matrix [14, 71]. As in previous iterations of that matrix, *Neuquenraptor* and *Unenlagia* were combined into a single operational taxonomic unit (OTU), even though we remain agnostic on the proposed synonomy of these genera and therefore refer to them as separate taxa throughout the remainder of this work. The final data matrix consists of 167 taxa and 884 characters, with some characters treated as ordered (see Supplementary Material). We also rescored characters 503 (state 1) and 876 (state 0) for *Timurlengia* and character 131 (state 1) for *U*. *comahuensis*. The matrix was analyzed in Tree analysis using New Technology (TNT) v. 1.5 [72, 73], with all characters treated as equally weighted. Following Napoli et al. [70], we employed a heuristic tree search strategy using “New Technology,” utilizing the sectorial search, ratchet, tree drifting, and tree fusing algorithms until it produced 20 hits on the shortest tree length. The most parsimonious trees (MPTs) were then subjected to a final round of tree bisection-reconnection (TBR) branch-swapping. Zero-length branches were collapsed during the analysis (rule 1 of Coddington & Scharff [74]). Unstable taxa were detected by implementing the IterPCR procedure [75], which provides a reduced consensus that does not include these taxa, as well as their alternative positions. We implemented the jackknife protocol employed by Pol & Goloboff [76] to calculate nodal support values.

## Results

### Systematic paleontology

Theropoda Marsh, 1881 [77].

Tetanurae Gauthier, 1986 [78].

Coelurosauria Huene, 1920 [79].

Paraves Sereno, 1997 [80].

Dromaeosauridae Matthew & Brown, 1922 [81].

Unenlagiinae Bonaparte, 1999 [82].

***Diuqin lechiguanae*** gen. et sp. nov.

ZooBank genus registration: urn: lsid: zoobank.org: act: BF5CDA11-1682-4E48-8A75-5661A6648B68.

ZooBank species registration: urn: lsid: zoobank.org: act:0972C0F4-E3DE-47E4-A589-DDC0B2D35138.

#### Etymology

Genus name: *Diuqin* (from Mapuzungun, the language of the Mapuce people indigenous to the region where the fossil was found), bird of prey. Species name: *lechiguanae*, after Lechiguana, the witch in the 1975 film *Nazareno Cruz y el Lobo* (directed by eminent Argentinian filmmaker Leonardo Favio) who foresaw that the film’s titular character would become a werewolf. Intended etymology: “Lechiguana’s bird of prey.”

#### Holotype

MUCPv 1401, a fragmentary but associated postcranial skeleton consisting of the posteriormost sacral vertebral neural arch, an anterior caudal vertebral neural arch, the nearly complete left humerus, and four unidentified fragments (at least two of which may be small pieces of vertebrae). Accessioned in the Museo de Ciencias Naturales of the Universidad Nacional del Comahue in Neuquén, Neuquén Province, Argentina to ensure free access to qualified researchers in perpetuity.

#### Locality and horizon

The specimen was collected from the isthmus between the southeast coast of Lago Barreales and the northwest coast of Lago Mari Menuco, in Neuquén Province, northwestern Patagonia, Argentina (Fig. 1), from a stratum of the Bajo de la Carpa Formation of the Neuquén Group (Upper Cretaceous: Santonian [30]).

#### Associated fauna

An isolated megaraptorid theropod tooth (MUCPv 1557; see below) and fragmentary bones of an indeterminate sauropod were also found near the site that yielded the *D*. *lechiguanae* holotype (MUCPv 1401). The megaraptorid tooth was found approximately 2–3 m from *Diuqin*, whereas the sauropod fragments were found some 10–12 m away.

#### Diagnosis

First unenlagiine theropod dinosaur to be discovered from the Bajo de la Carpa Formation (Neuquén Group, Upper Cretaceous), exhibiting the following autapomorphies and a unique combination of characters. *Autapomorphies*: (1) horizontal accessory lamina between spinopostzygapophyseal laminae on posteriormost sacral vertebra; (2) pair of elliptical, bilateral, dorsolaterally–ventromedially oriented foramina immediately anterolateral to base of neural spine in (at least) posteriormost sacral and anterior caudal vertebrae; (3) distolateral deltopectoral ridge of humerus arises on distal half of deltopectoral crest. *Unique combination of characters*: postzygapophyses of posteriormost sacral vertebra strongly posteriorly projected; humeral deltopectoral crest oriented anteriorly (also present in *Austroraptor cabazai*); absence of sulcus between deltopectoral crest and humeral shaft (also present in *Austroraptor*); sharp crest proximal to humeral ectepicondyle with proximally positioned tubercle (also present in *Buitreraptor*).

### Description

**Fig. 2:**
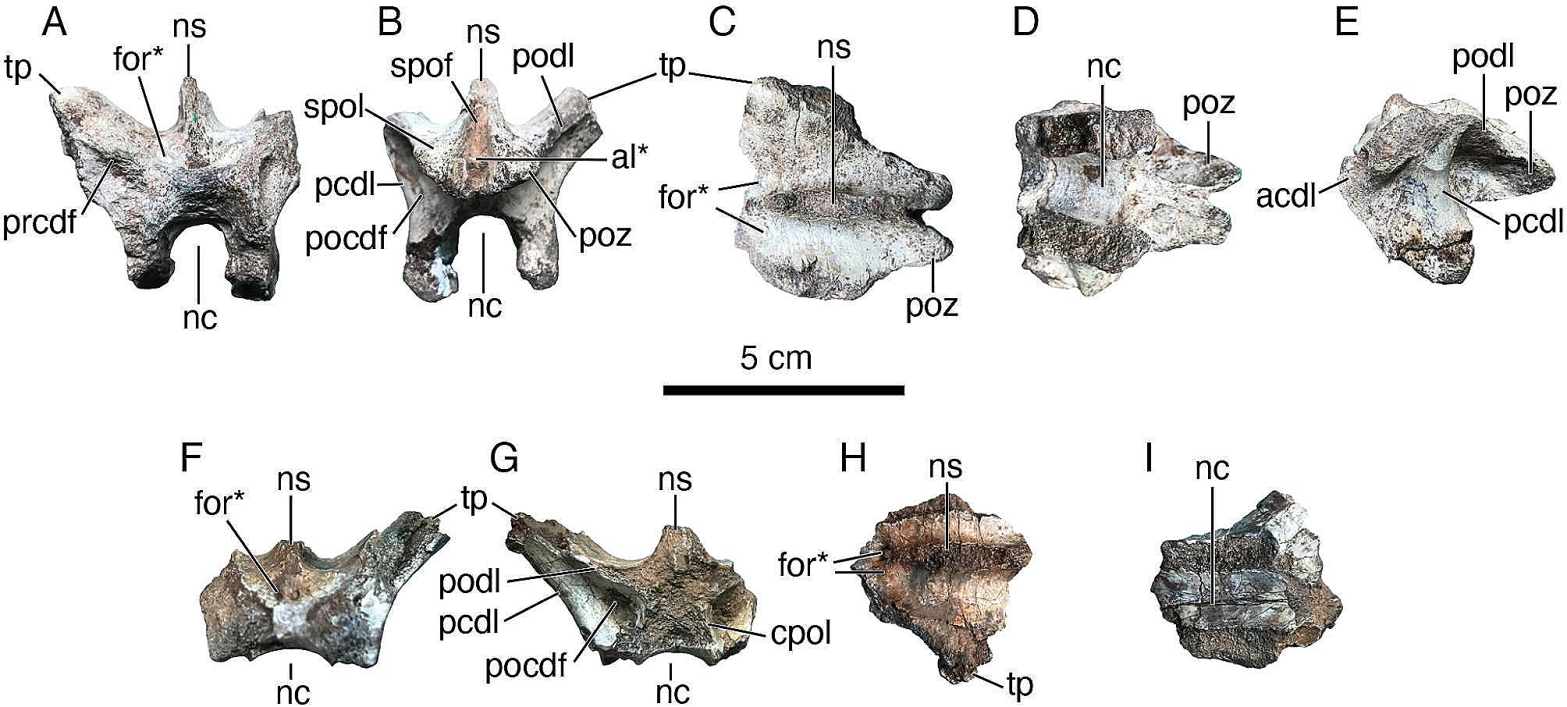
Postcranial axial skeletal elements of *Diuqin lechiguanae* gen. et sp. nov. (MUCPv 1401). **A**–**E**, posteriormost (last) sacral neural arch (MUCPv 1401/1) in anterior (**A**), posterior (**B**), dorsal (**C**), ventral (**D**), and left lateral (**E**) views. **F**–**I**, anterior caudal neural arch (MUCPv 1401/2) in anterior (**F**), posterior (**G**), dorsal (**H**), and ventral (**I**) views. Abbreviations: acdl, anterior centrodiapophyseal lamina; al, accessory lamina; cpol, centropostzygapophyseal lamina; for, foramen; nc, neural canal; ns, neural spine; pcdl, posterior centrodiapophyseal lamina; pocdf, postzygapophyseal centrodiapophyseal fossa; podl, postzygodiapophyseal lamina; poz, postzygapophysis; prcdf, prezygapophyseal centrodiapophyseal fossa; spof, spinopostzygapophyseal fossa; spol, spinopostzygapophyseal lamina; tp, transverse process. Asterisks indicate hypothesized autapomorphic features (the horizontal accessory lamina and bilateral neural arch foramina). Scale bar equals 5 centimeters

**Fig. 3:**
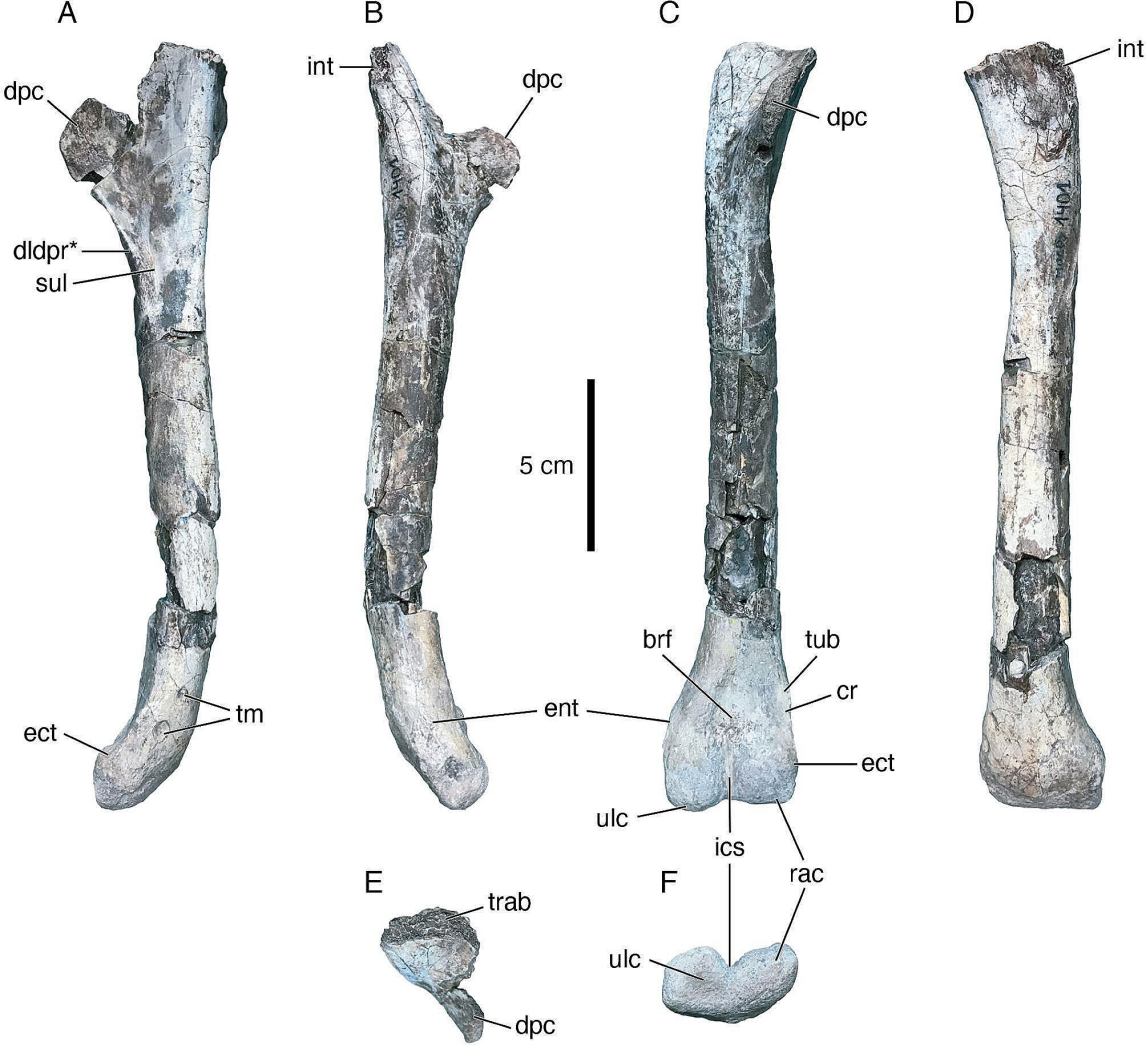
Left humerus of *Diuqin lechiguanae* gen. et sp. nov. (MUCPv 1401/4) in lateral (**A**), medial (**B**), anterior (**C**), posterior (**D**), proximal (**E**), and distal (**F**) views. Abbreviations: brf, brachial fossa; cr, crest; dldpr, distolateral deltopectoral ridge; dpc, deltopectoral crest; ect, ectepicondyle; ent, entepicondyle; ics, intercondylar sulcus; int, internal tuberosity; rac, radial condyle; sul, sulcus; tm, tooth marks; trab, trabecular tissue; tub, tuberosity; ulc, ulnar condyle. Asterisk indicates hypothesized autapomorphic feature (the distally arising distolateral deltopectoral ridge). Scale bar equals 5 centimeters

*Posteriormost sacral vertebra (MUCPv 1401/1) (*Fig. 2A–E; Table 1*).* Only the neural arch of this vertebra is preserved. Based on the morphology of the transverse processes, the lack of obvious external pneumatic features ventral to these processes, and the posterior elongation of the postzygapophyses, we regard this vertebra as the posteriormost (i.e., last) sacral. Moreover, this partial vertebra is similar to the posteriormost sacral of *Buitreraptor gonzalezorum* (see Gianechini et al. [15]: Fig. 3a), especially in that the postzygapophyses are situated close to the sagittal midline. In addition, the neural spine is not fused to that of the preceding sacral vertebra, as in the posteriormost sacrals of *Rahonavis ostromi* [10] and possibly *Unenlagia comahuensis* [66], although the preservation of the sacrum of the latter species is too poor to be certain of this feature. The neurocentral suture is open (Fig. 2A, B, D, E), and as such, the neural arch was unfused to its respective centrum at the time of death; consequently, the holotype of *Diuqin lechiguanae* is inferred to represent a somatically immature individual. The articular surface for the centrum is almost totally broken away, except for a small portion that remains on the posteroventral part of the left neural arch pedicle (Fig. 2D). This surface is rugose and ‘corrugated,’ showing evidence of an interdigitating suture.

**Table 1:** Measurements (millimeters) of vertebrae of *Diuqin lechiguanae* gen. et sp. nov. (MUCPv 1401). N/A = not applicable

Measurement	Sacral neural arch (MUCPv 1401/1)	Caudal neural arch (MUCPv 1401/2)
Anteroposterior length, neural spine	34.5	28.3
Transverse width, neural spine base	8.9	7.7
Anteroposterior length, anterior margin of transverse process to posterior end of postzygapophysis	47.1	N/A
Anteroposterior length, postzygapophyseal articular facet	16.4	N/A
Mediolateral width, postzygapophyseal articular facet	12.6	N/A
Anteroposterior length, neural canal	24.2	N/A
Transverse width, anterior opening of neural canal	10.1	N/A
Transverse width, posterior opening of neural canal	14.5	N/A

The neural arch preserves the bases of the transverse processes and neural spine as well as the complete postzygapophyses (Fig. 2A–E), whereas the prezygapophyses have been totally lost. In anterior view, the neural canal entry is ovoid in contour, being slightly taller than wide (Fig. 2A). The prezygapophyseal centrodiapophyseal fossae (prcdf *sensu* Wilson et al. [69]) are deepest medially. The transverse processes are directed dorsolaterally at an angle of approximately 41.5 degrees to the horizontal. On the anterolateral sides of the base of the neural spine there are two elliptical foramina that we regard as an autapomorphic feature of *Diuqin* (Fig. 2A, C). The neural spine is transversely thin and lacks spinoprezygapophyseal laminae (Fig. 2A, B).

In posterior view (Fig. 2B), the neural canal is oval in shape and is wider and taller than it is anteriorly. The rounded posterior centrodiapophyseal lamina (pcdl *sensu* Wilson [67]) and the sharp postzygodiapophyseal (podl) and centropostzygapophyseal (cpol) laminae delimit a deep, wide, and subtriangular postzygapophyseal centrodiapophyseal fossa (pocdf). The postzygapophyses are conjoined medially, forming a vertical lamina that lacks a hyposphene on its ventral part. The postzygapophyseal articular facets are ventrolaterally oriented. Two stout spinopostzygapophyseal laminae (spol) arise from the dorsal part of the postzygapophyses and converge anterodorsally. These laminae frame a subtriangular spinopostzygapophyseal fossa (spof) that is transversely narrow and anteroposteriorly deep. This fossa is subdivided by a small, horizontal lamina that links the medial surfaces of the postzygapophyses. This lamina, herein referred to as an accessory lamina (al), is not observed in other unenlagiines and is therefore regarded as an autapomorphy of *Diuqin*.

In dorsal view (Fig. 2C), the anterior foramina are obliquely oriented with the long axis directed medially. The posterior ends of the postzygapophyses diverge slightly. The neural spine base is transversely widest in the vicinity of its anteroposterior midpoint.

In ventral view (Fig. 2D), the neural canal is transversely wider than the neural arch pedicles, which diverge posterolaterally. The articular facet of the postzygapophysis is oval in contour, being substantially longer anteroposteriorly than wide mediolaterally.

As observed in lateral view (Fig. 2E), the transverse processes are situated on the anterior part of the neural arch and are less than half the anteroposterior length of the latter. The ventral surface of each process has a shallow depression that lacks fossae or foramina. The anterior centrodiapophyseal lamina (acdl) is stout laterally, forming a projection that is oval and anteroventrally–posterodorsally elongate in cross-section. This projection may have articulated with the medial side of the ilium. The pcdl is robust but more homogeneous in thickness than the acdl. The pcdl is inclined anterodorsally–posteroventrally and triangular in cross-section. The podl is stout and horizontal in lateral view. The postzygapophyses are strongly posteriorly projected, far surpassing the posterior base of the neural arch. The base of the neural spine is half the anteroposterior length of the neural arch, at least as the latter is preserved.

*Anterior caudal vertebra (MUCPv 1401/2) (*Fig. 2F–I; Table 1*).* This vertebra preserves only part of the neural arch, consisting of the roof of the neural canal, the partial left transverse process, and the base of the neural spine. Based on the morphology and orientation of the transverse process and the absence of fossae or laminae ventral to this structure, we regard this vertebra as an anterior element of the caudal series.

The preserved dorsal margin of the neural canal implies that the canal was large (Fig. 2F). The left transverse process is robust and dorsolaterally inclined at an angle of roughly 45 degrees. Anterior to the neural spine, there is a shallow and anteroposteriorly elongate spinoprezygapophyseal fossa (sprf), delimited dorsolaterally by vestigial spinoprezygapophyseal laminae (sprl). Within the sprf, two small foramina open bilaterally, separated by the ventral base of the prespinal lamina (prsl). These foramina are absent in other unenlagiines and are therefore regarded as autapomorphic of *Diuqin*. The prcdf is in a similar position as in the sacral vertebra but contains several foramina.

Both pocdf are preserved, with the left being in better condition (Fig. 2G). This fossa is smaller than that of the sacral vertebra but shows a similar morphology in being delimited by the same laminae. The pocdf has a small internal foramen.

The dorsal surface of the neural arch is transversely concave between the bases of the transverse processes and that of the neural spine. Judging from the morphology of its broken base, the neural spine appears to have extended across much of the anteroposterior length of the neural arch (Fig. 2H). As in MUCPv 1401/1, its base is widest near its anteroposterior midpoint. Broken surfaces of MUCPv 1401/2 reveal that this neural arch is internally composed of camellate tissue (Fig. 2I).

*Humerus (MUCPv 1401/4) (*Fig. 3; Table 2*).* The most complete bone of *Diuqin* is the left humerus, which is missing only parts of the proximal end and deltopectoral crest. There are no synapomorphies in the humerus of unenlagiines described by previous authors [14, 17, 83]. Nevertheless, that of *Diuqin* can be unequivocally assigned to Unenlagiinae based on the following combination of characters: (1) deltopectoral crest proximodistally short with respect to the humerus as a whole (as in the *Buitreraptor* holotype and *Unenagia*); (2) presence of a distolateral ridge on the lateral surface of the deltopectoral crest (as in *Buitreraptor* and *U*. *comahuensis*); (3) distal end of humerus curved anteriorly (as in *Austroraptor* and a referred specimen of *Buitreraptor*); and (4) differentiated and distally extended distal flexor process (highly developed in *Rahonavis* and *Buitreraptor*, absent in most non-avian paravians such as *Deinonychus antirrhopus*; see [10, 15]) (M. Motta pers. comm. 2024). Based on the proximodistal length of the preserved portion, when complete, the humerus of *Diuqin* was probably longer than that of *Unenlagia paynemili* but shorter than those of *U*. *comahuensis* and *Austroraptor cabazai* (Table 2). The proximal end of the humerus is posterolaterally inclined. In proximal view (Fig. 3E), its anterior and posterior margins are sigmoid, narrowing towards the deltopectoral crest. In this view, the broken proximal end of the humerus exposes internal bone tissue consisting of hollow spaces separated by thin trabeculae. Although much of the internal tuberosity is missing, it seems to have projected posteromedially. The deltopectoral crest is directed anteriorly, forming an angle of 90 degrees with respect to the humeral head.

**Fig. 4:**
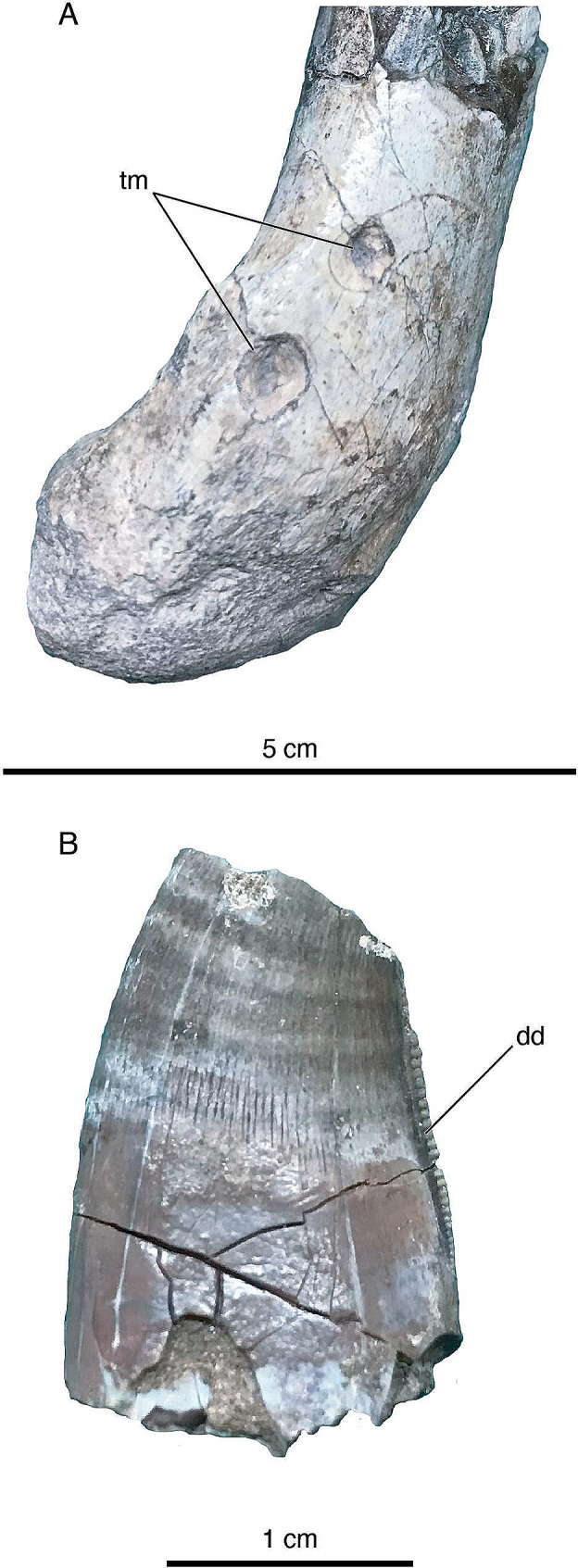
**A**, tooth marks (‘punctures’ *sensu* Gianechini & de Valais [60]) on distal end of lateral surface of humerus of *Diuqin lechiguanae* gen. et sp. nov. (MUCPv 1401/4). **B**, isolated tooth of Megaraptoridae indet. (MUCPv 1557) discovered near holotype of *Diuqin lechiguanae* gen. et sp. nov. (MUCPv 1401). Abbreviations: dd, distal denticles; tm, tooth marks. Scale bar equals 5 centimeters in **A**; 1 centimeter in **B**

**Table 2:** Measurements (millimeters) of the humerus of *Diuqin lechiguanae* gen. et sp. nov. (MUCPv 1401/4) and other unenlagiine and probable unenlagiine taxa. * = humerus incomplete, measurement as preserved; ** = humerus incomplete, measurement estimated; N/A = not applicable . Sources: *Austroraptor cabazai*: MML 195 (Novas et al. [23]: table 1), MML 220 (Currie & Paulina Carabajal [56]: table 1); *Buitreraptor gonzalezorum*: MPCA 245 (Novas et al. [23]: table 1; Gianechini et al. [15]: table S1); *Rahonavis ostromi*: FMNH PA 746, UA 9604 (Forster et al. [10]: table 6); *Unenlagia comahuensis*: MCF-PVPH 78 (Novas et al. [66]: table 1); *Unenlagia paynemili*: MUCPv 349 [20]

Measurement/ taxon (specimen)	*Diuqin lechiguanae* (MUCPv 1401/4)	*Buitreraptor gonzalezorum* (MPCA 245)	*Unenlagia comahuensis* (MCF-PVPH 78)	*Unenlagia paynemili* (MUCPv 349)	*Austroraptor cabazai* (MML 195)	*Austroraptor cabazai* (MML 220)	*Rahonavis ostromi* (FMNH PA 746)	*Rahonavis ostromi* (UA 9604)
Proximodistal length, total	200.0*	134.9	270.0**	217.0	262.0	232.0*	N/A	N/A
Anteroposterior width, deltopectoral crest	43.5	N/A	N/A	N/A	N/A	N/A	N/A	N/A
Anteroposterior width, midshaft	18.0	N/A	N/A	17.5	N/A	N/A	5.3	5.4
Anteroposterior width, distal	16.0	N/A	N/A	N/A	N/A	N/A	9.0	9.7
Mediolateral width, proximal	21.0	N/A	N/A	N/A	N/A	N/A	N/A	N/A
Mediolateral width, midshaft	16.5	6.0	17.0	14.0	22.0	N/A	7.8	7.3
Mediolateral width, distal	34.0	N/A	N/A	N/A	N/A	N/A	16.2	16.9
Ratio total length/midshaft width	12.1*	22.5	15.9**	15.5	11.9	N/A	N/A	N/A

The anterior surface of the humerus is smooth and flat medial to the deltopectoral crest. However, this surface becomes slightly bulbous towards the medial margin of the bone (Fig. 3B, C). In tandem with the medial surface of the deltopectoral crest, this area has been interpreted as the insertion of the M. coracobrachialis in other non-avian theropods [84–88]. The anteriormost end of the deltopectoral crest has a vaguely ‘D-shaped’ contour, with the medial rim convex and the lateral rim slightly concave; moreover, this end of the crest curves laterally (Fig. 3C). Distally, the deltopectoral crest forms a laterally directed ridge (Fig. 3A). The humeral shaft is straight, and its cross-section is subcircular at midshaft but subtriangular more distally (being narrower anteroposteriorly than mediolaterally in this region). Distally (Fig. 3F), the ulnar and radial condyles are anteriorly projected and separated by a deep intercondylar sulcus (our identification of the distal condyles follows previous authors (e.g., [15, 71, 85, 89, 90]). The ulnar condyle is more distally projected than the radial condyle (Fig. 3C, D). The condyles also differ in shape, with the ulnar condyle subrectangular and the radial condyle subquadrangular in anterior view (Fig. 3C). Proximal to the distal condyles, the brachial (= anterodistal) fossa is shallow and triangular in outline. The entepicondyle is low and medially directed. The ectepicondyle is on the proximolateral part of the radial condyle and is anteriorly projected. The ectepicondyle is poorly developed, similar to *Buitreraptor* and *Rahonavis*, differing from its better-developed counterpart in *Deinonychus*. A sharp longitudinal crest that may be homologous to the processus supracondylaris dorsalis of birds [91] extends from the proximal part of the ectepicondyle. A small tubercle arises from the proximalmost part of this crest (Fig. 3C).

In lateral view (Fig. 3A), the deltopectoral crest is triangular, with its proximal and distal margins meeting at a right angle. The distal margin of the deltopectoral crest forms a pronounced ridge, here termed the distolateral deltopectoral ridge. It arises from the distal half of the distal margin of the deltopectoral crest and extends across the anterolateral one-third of the shaft, a condition that is herein considered an autapomorphy of *Diuqin*. Between this ridge and the shaft there is a deep sulcus that contains proximodistally aligned longitudinal striae. The angle between the deltopectoral crest and the shaft is 144 degrees.

In medial view (Fig. 3B), the deltopectoral crest occupies the proximal ∼one-third of the humerus. The crest merges gently with the humeral shaft, without the presence of a sulcus. Though the internal tuberosity is partly lost, it was clearly stout and seemingly directed posteromedially. The shaft is straight, but the distal end of the bone is anteriorly bowed. In this view, the distal part of the shaft is anteroposteriorly narrow when compared to the mediolateral width, rendering it oval in cross section.

In posterior view (Fig. 3D), the preserved distal portion of the internal tuberosity is placed more proximally than the apex of the deltopectoral crest. There is a small depression near the distal end of the internal tuberosity. Lateral to this depression the shaft is convex.

*Indeterminate fragments.* The *Diuqin* holotype also includes four small fragments of bone that collectively preserve little osteological information. At least two of these may correspond to pieces of vertebrae (potentially transverse processes), but this identification is extremely tentative and as such the fragments are not described further herein.

#### Tooth marks

Two subconical depressions herein interpreted as tooth marks are clearly visible on the distal end of the lateral surface of the humerus of the *Diuqin lechiguanae* holotype (Figs. 3A and 4A). In each, the apex of the ‘cone’ points towards the interior of the humerus. The marks are one and two millimeters deep and five and four millimeters in maximum diameter, respectively, being separated from each other by ten millimeters. There are no grooves on the inwardly collapsed cortex, differing from the *Buitreraptor gonzalezorum* specimen MPCA 470-75 that shows several furrows [60]. Similar marks observed on some bones of the *Buitreraptor* holotype (MPCA 245) have been termed ‘punctures’ and attributed to crocodyliform or mammalian trace makers [60]. Around the punctures on the *Diuqin* humerus, there is no evidence of bone reaction or healing, such as the formation of new bone, or lysis, such as osteophytes, exostosis, or empty spaces (e.g., cloacae) representing fibriscesses. Therefore, these marks must have been produced either postmortem or very shortly before death.

#### Associated tooth

At the type locality of *Diuqin lechiguanae* (approximately 2–3 m from the holotype, MUCPv 1401), we also recovered an isolated tooth (MUCPv 1557; Fig. 4B). The preserved region is 22.69 mm in apicobasal height and is formed by part of the crown (12.21 mm) and a smaller part of the root (10.48 mm); approximately the apical one-third of the crown is missing. The tooth is labiolingually compressed, though not as strongly as in some theropods, with a Crown Base Ratio (CBR; *sensu* Smith et al. [92]; i.e., ratio of labiolingual to mesiodistal dimension measured at the base of the crown) of approximately 0.61. MUCPv 1557 is suboval in cross-section, with the mesial region slightly labiolingually broader than the distal, though there is also a modest labiolingual constriction near the mesiodistal midline. The labial surface is more convex than the lingual. The tooth is recurved (i.e., in labial or lingual view, its mesial margin is convex and its distal margin is concave), indicating that it probably does not pertain to the premaxillary arcade. The concave distal margin is shared with megaraptorid tetanurans such as *Orkoraptor burkei* [93], *Megaraptor namunhuaiquii* [94], and *Murusraptor barrosaensis* [95] and contrasts the straight distal margin typical of abelisaurid crowns [96–98]. The mesial carina lacks denticles, a character that is generally rare among non-avian theropods but is again shared with megaraptorids (e.g., all known teeth of *Orkoraptor* and *Megaraptor*, most teeth of *Murusraptor*) [99]. The distal carina approaches the lingual surface. Its most apical preserved denticles (corresponding to those near midheight of the crown in the complete tooth) are larger than the basal denticles; the apicalmost preserved denticles have a density of two per millimeter whereas that of the basal denticles is five per millimeter.

MUCPv 1557 differs in multiple regards from the teeth of non-avian Paraves. The crown base lacks the constriction observed in several troodontids, which also generally have much larger, hooked denticles [99]. The presence of denticles and transverse undulations in MUCPv 1557 precludes its assignment to halszkaraptorine or unenlagiine dromaeosaurids [58, 83], whereas the absence of longitudinal flutes/ridges and the presence of transverse undulations rules out Microraptorinae as well [99]. Also, there are several differences with eudromaeosaurians; for instance, the denticles are not hook-shaped as in *Atrociraptor marshalli* and *Saurornitholestes langstoni*, nor are there lateral ridges as in several other species (e.g., *Acheroraptor temertyorum*, *Bambiraptor feinbergi*, *Linheraptor exquisitus*, *Velociraptor mongoliensis*) [99]. Furthermore, although the tooth is only partially preserved, it is substantially larger than those of other known unenlagiines [2, 23, 57, 58], even *Austroraptor*, which is widely considered the largest-bodied member of this clade [23, 56, 57], and larger than *Diuqin* based on relative humeral length (Table 2).

Accordingly, based on (1) the morphological differences with paravian teeth enumerated above, and (2) its relatively large size, MUCPv 1557 almost certainly does not pertain to *Diuqin* or any other unenlagiine taxon. Instead, the specimen strongly resembles the teeth of megaraptorids, particularly in its concave distal margin and lack of serrations on the mesial carina. Moreover, Gianechini et al. [100] and Meso et al. [97] documented a similar tooth (MPCA 247) from the La Bonita site of the Bajo de la Carpa Formation, with the latter authors referring the specimen to Megaraptoridae indet. based on the results of their dentition-based cladistic, cluster, and discriminant analyses. MUCPv 1557 shares with MPCA 247 the presence of mesiodistally oriented undulations (enamel wrinkles [101]) extending across the labial and lingual surfaces of the crown. Enamel wrinkles are widely present in abelisaurids and carcharodontosaurids (e.g [99, 102, 103]), but are also evident in several allosauroids, including neovenatorids, as well as in selected coelurosaurs such as tyrannosauroids [99]. MPCA 247 and MUCPv 1557 are closely comparable in all other respects as well, including CBR (0.54 in MPCA 247; 0.61 in MUCPv 1557). Accordingly, we refer the latter tooth to Megaraptoridae indet.

The holotypic specimen of the megaraptorid *Tratayenia rosalesi* [35] was also collected from the Bajo de la Carpa Formation, from a site only 18 km north of the locality that yielded *Diuqin* and MUCPv 1557. Unfortunately, *Tratayenia* is known only from postcranial elements, precluding a definitive comparison with MUCPv 1557. Still, given the morphology of the new tooth—consistent with that of other megaraptorid teeth—as well as its stratigraphic and geographic provenance, we consider it plausible that MUCPv 1557 (and perhaps MPCA 247) could pertain to *Tratayenia*. Another megaraptorid specimen from the Bajo de la Carpa Formation, the fragmentary partial skeleton MAU-Pv-CO-659, was recently reported by Méndez et al. [104], adding to the evidence of these large-bodied theropods in this stratigraphic unit.

## Discussion

### Comparisons with other paravians

**Fig. 5:**
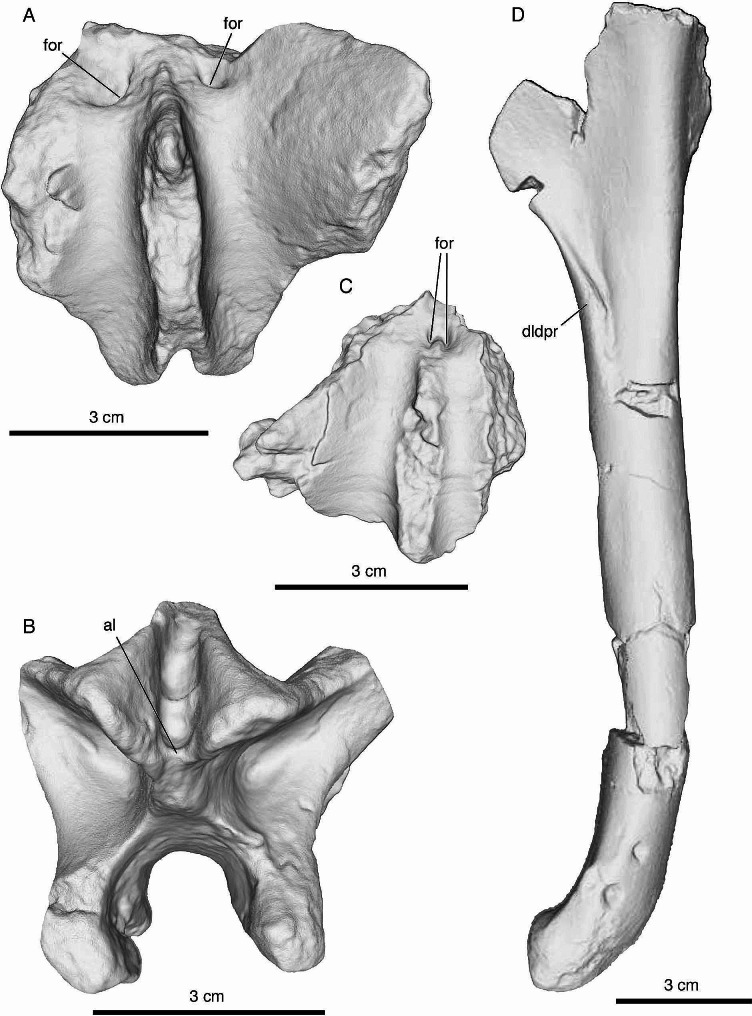
Autapomorphies proposed for *Diuqin lechiguanae* gen. et sp. nov. (MUCPv 1401), with images generated from three-dimensional scans of selected bones. **A**–**B**, posteriormost (last) sacral neural arch (MUCPv 1401/1) in dorsal (**A**) and posterior (**B**) views. **C**, anterior caudal neural arch (MUCPv 1401/2) in dorsal view. **D**, left humerus (MUCPv 1401/4) in lateral view. Abbreviations: al, accessory lamina; dldpr, distolateral deltopectoral ridge; for, foramen. Scale bars equal 3 centimeters

Among other unenlagiines and probable unenlagiines, sacral vertebrae are preserved in *Unenlagia comahuensis*, *Rahonavis ostromi*, and *Buitreraptor gonzalezorum*, although their state of preservation is generally poor. The posteriormost sacral vertebra of *Diuqin lechiguanae* (MUCPv 1401/1) has transverse processes with a dorsal inclination of the articular surface for the ilium, implying that the postacetabular process of the latter was laterally deflected. This feature also occurs in *Buitreraptor*. Moreover, the last sacral transverse processes of *Diuqin*, *Buitreraptor*, and *U*. *comahuensis* are stout and dorsolaterally directed, though in *Diuqin* the anteroposterior development of these processes is reduced compared to the conditions in the other two taxa. The morphology of the posterior end of the postzygapophyses of *Diuqin* also slightly differs from that in *Buitreraptor*, since in the former taxon they are more closely placed. The foramina located anterolateral to the base of the neural spine (Figs. 2A and C and 5A) are not observed in any of the preserved sacral vertebrae of *Buitreraptor* or *U*. *comahuensis*, although in the latter taxon the sacrals are poorly preserved. However, in the anteriormost sacral vertebra of *U. comahuensis*, there are bilateral foramina located more lateral to the neural spine, which are similar to those present in the posteriormost dorsal vertebra of this species [66]. In *Rahonavis*, the sacral neural arches lack foramina [10]. The presence of similar foramina in sacral neural arches has not been reported in other paravians, although the dorsal aspect of the sacrum is usually not described in great detail in these theropods.

*Diuqin* appears to differ from other unenlagiines in having a last sacral vertebra with an accessory lamina between the spinopostzygapophyseal laminae (Figs. 2B and 5B). Nevertheless, the absence of this feature in other members and probable members of the clade is difficult to definitively confirm, in that the sacral neural arches of *U*. *comahuensis* and *Rahonavis* are poorly preserved; moreover, in *Buitreraptor* (MPCA 245, the only specimen of this taxon with preserved posterior sacral postzygapophyses), the posterior face of the posteriormost sacral is largely obscured by sediment and the articulated first caudal (FAG pers. obs.). It is challenging to confirm the absence of the accessory lamina in other paravians as well, given that, in many taxa (e.g., selected eudromaeosaurs [105], the microraptorine *Microraptor zhaoianus* [106], the troodontids *Zanabazar junior* [107] and *Mei long* [108]), the sacral zygapophyses are fused into a longitudinal ridge, the last sacral neural arch is damaged, and/or the known specimens are preserved largely in two (as opposed to three) dimensions.

Among definitive and probable Unenlagiinae, caudal vertebrae are preserved only in *Diuqin*, *Buitreraptor*, *Rahonavis*, and *U*. *comahuensis*; therefore, the morphological information provided by the new Bajo de la Carpa Formation taxon is significant for knowledge of the group more broadly. The caudal vertebrae of these paravians exhibit clear differences. In *Buitreraptor*, the transverse process is thinner and less dorsally inclined than in *Diuqin*. The former taxon also has more developed sprl that delimit a deeper sprf. In *Buitreraptor*, the prcdf and pocdf are difficult to recognize because the vertebrae remain articulated and largely unprepared. In *Rahonavis*, the first caudal transverse process differs in orientation from that of *Diuqin* since it is almost horizontally placed and posteriorly directed. Furthermore, this probable Madagascan unenlagiine lacks foramina at the anterior base of the neural spine. *U*. *comahuensis* preserves only one caudal vertebra, which has been interpreted as the first [66]. This vertebra also has a robust transverse process, although its base is anterodorsally–posteroventrally inclined, thus differing from that of *Diuqin*. Also, the neural arch of the *U*. *comahuensis* caudal is dorsoventrally lower and the base of the transverse process is much closer to that of the neural arch than in *Diuqin*. Unfortunately, the remainder of the neural arch of the *U*. *comahuensis* caudal vertebra is not preserved, precluding further comparisons.

**Fig. 6:**
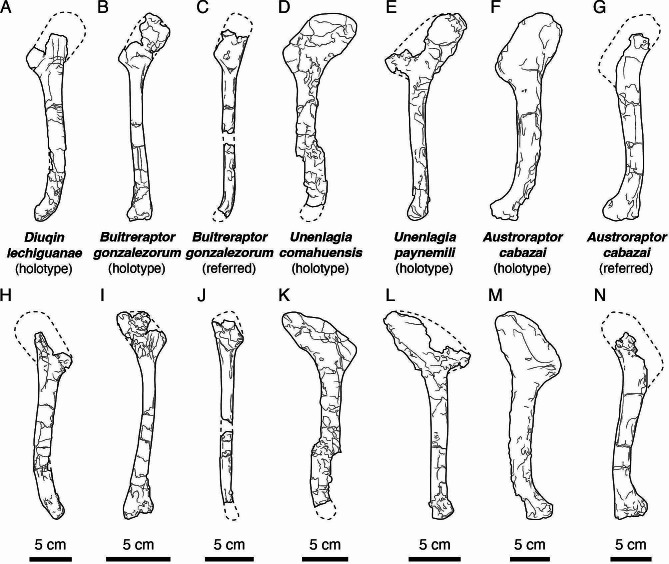
Comparative line drawings of unenlagiine left humeri in lateral (**A**–**G**) and medial (**H**–**N**) views. **A**, **H**, *Diuqin lechiguanae* gen. et sp. nov. (MUCPv 1401, holotype). **B**, **I**, *Buitreraptor gonzalezorum* (MPCA 245, holotype, right humerus reversed, after Gianechini et al. [15]). **C**, **J**, *Buitreraptor gonzalezorum* (MPCN-PV 598, referred specimen, right humerus reversed, after Novas et al. [18]). **D**, **K**, *Unenlagia comahuensis* (MCF-PVPH 78, holotype, after Gianechini [57]). **E**, **L**, *Unenlagia paynemili* (MUCPv 349, holotype, after Gianechini [57]). **F**, **M**, *Austroraptor cabazai* (MML 195, holotype, right humerus reversed, after Gianechini [57]). **G**, **N**, *Austroraptor cabazai* (MML 220, referred specimen, right humerus reversed, after Gianechini [57]). Scale bars equal 5 centimeters

The humerus is an important element for deciphering unenlagiine osteology, taxonomy, and phylogeny; for example, some of the main differences observed between the two known *Unenlagia* species (*Unenlagia paynemili* and *U*. *comahuensis*) are found in this element. The humerus of *Diuqin* is more robust than those of *U*. *paynemili* and *Buitreraptor* but more gracile than that of *Austroraptor cabazai*, most closely resembling *U*. *comahuensis* in this regard (Fig. 6). The sigmoidal curvature of the bone in lateral and medial views resembles that observed in the humeri of other dromaeosaurids [106, 109, 110]. Among unenlagiines, a similar shape is present in *Austroraptor* (Fig. 6F, G, M, N), whereas the humeri of *Buitreraptor* and *U*. *paynemili* are, by contrast, straight in lateral and medial views (Fig. 6B, C, E, I, J, L). As in many non-avian maniraptorans [109, 111, 112], as well as early diverging avialans such as *Archaeopteryx lithographica* and *Confuciusornis sanctus* [113, 114], the proximal portion of the humerus is posteriorly inclined with respect to the long axis of the shaft, although this inclination is more pronounced in avialans. Previous authors (e.g., [20]) have used the angle at which the anterodistal margin of the deltopectoral crest and the anterior margin of the humeral shaft meet when the humerus is observed in lateral view to differentiate between various unenlagiine taxa. This angle is 144 degrees in *Diuqin*, 148 degrees in *Buitreraptor*, approximately 140 degrees in *U*. *comahuensis* and *Austroraptor*, and 116 degrees in *U*. *paynemili*; as such, the new Bajo de la Carpa Formation form resembles most other unenlagiines (with the exception of *U*. *paynemili*) in this regard (Fig. 6A–G). The anterior orientation of the deltopectoral crest of *Diuqin* resembles the condition in *Austroraptor* but differs from that in *Buitreraptor*, *U*. *comahuensis*, and *U*. *paynemili*, in which this crest is anterolaterally directed. The deltopectoral crest is also anteriorly directed in most other dromaeosaurids (e.g., *Bambiraptor feinbergi*, *Deinonychus*, *Sinornithosaurus millenii* [109, 115, 116]) as well as in most troodontids (e.g., *Sinornithoides youngi*, *Linhevenator tani* [117, 118]). The apex of the deltopectoral crest of *Diuqin* is more pointed in lateral view than that of *Austroraptor* (Fig. 6A, F, H, M).

The distolateral deltopectoral ridge present in *Diuqin* (Figs. 3A and 5D) also occurs in *U*. *comahuensis* and *Buitreraptor* but is absent in *Austroraptor* (Fig. 6A–D, F). *U*. *paynemili* seems to have a reduced ridge, although this region is too poorly preserved to ascertain its original morphology in this species. The distolateral deltopectoral ridge possibly represents the distal limit of the insertion of M. deltoideus clavicularis, given that this muscle is inferred to have inserted on the lateral surface of the deltopectoral crest in other non-avian theropods [84–86, 88]. The specific morphology of the distolateral deltopectoral ridge differs between *Diuqin*, *U*. *comahuensis*, and *Buitreraptor*. In *U*. *comahuensis*, this ridge is relatively weakly developed and extends nearly to the anterior border of the humeral shaft. In *Diuqin* and *Buitreraptor*, by contrast, this ridge is directed towards the center of the lateral surface. In *Diuqin*, the anterior and posterior margins of the distolateral deltopectoral ridge diverge distolaterally, whereas in *Buitreraptor* and *U*. *comahuensi*s these structures are subparallel. Furthermore, the ridge originates more distally in *Diuqin* than in *Buitreraptor* and *U*. *comahuensis*, in which it arises from the apex of the deltopectoral crest. The extension of the ridge onto the anterolateral surface of the humeral shaft is not observed in other unenlagiines (Fig. 5D). The distolateral deltopectoral ridge is also present in some non-unenlagiine dromaeosaurids, such as *Deinonychus* [109], but absent in others, such as *Bambiraptor* (FAG pers. obs. of AMNH FARB 30556). Also, a similar ridge is observed in some troodontids, such as *Linhevenator* [118], and possibly also in the humeri of the avialans *Archaeopteryx* (NHMUK 37001) and *Confuciusornis* (*sensu* Novas et al. [66]). In non-unenlagiine dromaeosaurids (e.g., *Deinonychus*, *Velociraptor mongoliensis*, *Linheraptor exquisitus*), a ridge extends distal to the deltopectoral crest on the posterior surface of the humeral shaft and delimits a medially located groove [109, 110, 116, 119], a condition similar to that observed in *Diuqin*.

Although the internal tuberosity of the *Diuqin* humerus is mostly broken away, the distal base of this structure is similar in anteroposterior thickness to those of *U*. *comahuensis* and *Austroraptor*, thus differing from the conditions in *Buitreraptor* and *U*. *paynemili* in which the base of the tuberosity is slenderer. Moreover, the internal tuberosity of *Diuqin* was clearly placed proximally. In *Buitreraptor*, *U*. *paynemili*, and *Austroraptor*, the internal tuberosity is situated proximal with respect to the apex of the deltopectoral crest (Fig. 6I, L, M), whereas in *U*. *comahuensis* these structures are at the same proximodistal level (Fig. 6K). As in *Austroraptor*, *Diuqin* lacks the sulcus medial to the deltopectoral crest observed in *Buitreraptor*, *U*. *comahuensis*, and *U*. *paynemili*. This sulcus is also present in non-unenlagiine dromaeosaurids such as *Bambiraptor* and other paravians such as *Balaur bondoc* (Brusatte et al., 2013). In many non-avian theropods (e.g., *Tawa hallae*, *Majungasaurus crenatissimus* [85, 86]), the insertion area of the M. coracobrachialis is generally a concave region distal to the humeral head. In *Diuqin* and especially *U*. *paynemili*, however, a bulbous structure delimits this area medially. The posterior surface of the humerus of *Diuqin* is convex from the proximal part of the shaft nearly to the missing humeral head, a condition shared with *Buitreraptor* and *Austroraptor*. In *U*. *paynemili* and *U*. *comahuensis*, however, this convexity is reduced to a conspicuous ridge; this ridge reaches the proximal articular surface in the former species.

The distal part of the humeral shaft is anteriorly curved in *Diuqin*, a feature that is also observed in *Austroraptor* (Fig. 6A, F, G, H, M, N). In other unenlagiines, by contrast, the distal end of the shaft is straight in lateral or medial view, following the same proximodistal axis as the remainder of the shaft (Fig. 6B–E, I–L). The ectepicondyle of the *Diuqin* humerus is more conspicuous than those of *Buitreraptor* and *U*. *paynemili*. The crest proximal to the ectepicondyle is observed in *Diuqin*, *Buitreraptor*, and *U*. *paynemili*, but is best developed in the former taxon, which also has a small proximal tubercle that is absent in *U*. *paynemili* and less marked in *Buitreraptor*. A similar crest on the distolateral humerus is also observed in other paravians such as *Bambiraptor* and *Balaur*. This crest may be homologous to the processus supracondylaris dorsalis of some Avialae (e.g., [91]). This process is also present in some basal avialans (e.g., isolated humeri from the Late Cretaceous of Madagascar [120]), and Ornithurae such as *Limenavis patagonica*. The distal humeral condyles of the new Bajo de la Carpa taxon are more developed than in *U*. *paynemili* and *Austroraptor*. The ulnar condyle of *Diuqin* extends further distally than the radial condyle, although it does not have the projected conical process observed in *Buitreraptor* and *Rahonavis* [10]. In contrast to basal avialans such as *Confuciusornis* and *Sapeornis chaoyangensis* [113, 121], enantiornithines such as *Eoalulavis hoyasi* [122], and ornithuromorphs such as *Limenavis* [123], the condyles are not better developed on the anterior surface. A brachial fossa is apparently present in the humeri of some dromaeosaurids such as *Deinonychus* and *Bambiraptor* [109, 116], in *Balaur* [71, 124], in basal avialans such as *Confuciusornis*, *Sapeornis*, and *Alcmonavis poeschli* [113, 114], and in Patagonian Cretaceous birds such as the enantiornithine *Neuquenornis volans* and the ornithuromorph *Limenavis*. This fossa is also observed in *Buitreraptor* (MPCA 245), although in this specimen the surface of the bone is fractured in this area and thus it is difficult to ascertain the true depth of this fossa. The presence of this fossa in *Diuqin* and *Buitreraptor* could therefore represent a condition intermediate between that in other non-avian theropods and the deep brachial fossa present in Avialae.

### **Phylogenetic analysis**

**Fig. 7:**
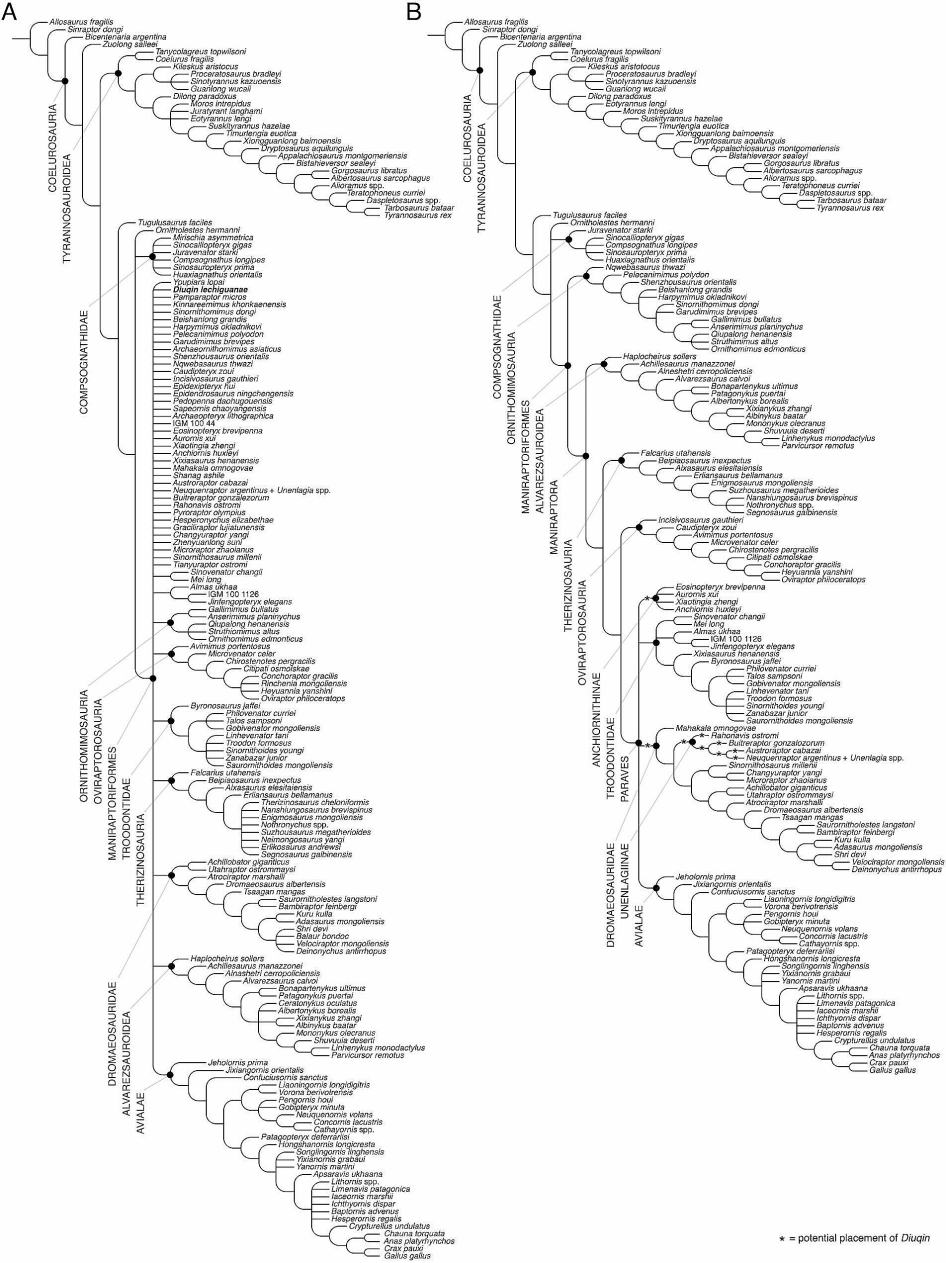
Phylogenetic affinities of *Diuqin lechiguanae* gen. et sp. nov. within Coelurosauria. Depicted topologies are the strict (**A**) and reduced (**B**) consensus of >50,000 most parsimonious trees of 3,711 steps recovered via analysis of a dataset originally consisting of 167 theropod taxa and 884 morphological characters. Asterisks (*) in **B** indicate possible phylogenetic positions of *D*. *lechiguanae*

To more definitively assess the systematic position of *Diuqin lechiguanae* within Paraves, we conducted a phylogenetic analysis (see Materials and methods above for methodological details). The analysis yielded more than 50,000 MPTs of 3,711 steps (consistency index = 0.302; retention index = 0.764). The strict consensus of these trees (Fig. 7A) presents several polytomies within Coelurosauria, with the largest of these including a multitude of maniraptoriform taxa. However, most individual clades within Maniraptoriformes maintain their monophyly (with exceptions including Maniraptora, Paraves, and Unenlagiinae, among others). The IterPCR analysis identified 25 unstable (i.e., ‘wildcard’) OTUs (see Supplementary Material), three of which are the unenlagiines *Diuqin*, *Pamparaptor micros*, and *Ypupiara lopai*. The reduced strict consensus tree recovered after the pruning of these 25 OTUs retains several polytomies, but all principal coelurosaurian clades are supported (Fig. 7B). Minor polytomies were recovered among the following groups: (1) early diverging tyrannosauroids; (2) compsognathids; (3) late diverging ornithomimids; (4) late diverging therizinosaurids; (5) anchiornithines; and (6) troodontids, especially among later diverging members of that clade. *Diuqin* occupies various positions within Paraves, being recovered as either the (1) sister taxon of Anchiornithinae; (2) sister taxon of Dromaeosauridae; (3) sister taxon of Unenlagiinae; or (4) related to each of the individual OTUs within the latter clade (Fig. 7B). *Ypupiara* was recovered as the sister taxon of Oviraptorosauria or *Buitreraptor*, whereas *Pamparaptor* was postulated as the sister taxon of Anchiornithinae, Troodontidae, or the clade formed by the microraptorine dromaeosaurids *Changyuraptor yangi* and *Microraptor zhaoianus*. It is also noteworthy that, among the unstable taxa, the maniraptoriforms *Pyroraptor olympius* and *Shanag ashile* were recovered as related to Unenlagiinae or nested within the latter clade (see Supplementary Material, Fig. S1). In the case of *Pyroraptor* from the Upper Cretaceous of France, Hartman et al. [125] were the first to propose this form as an unenlagiine. If this hypothesis is supported by future work, then, alongside Abelisauridae, Unenlagiinae might represent a typically Gondwanan non-avian theropod lineage that was also present in Europe during the latest Cretaceous.

The jackknife analysis found 33 unstable taxa including *Diuqin*, with an average support value of 76.9, and several minor polytomies plus a major polytomy at the base of Dromaeosauridae. Unenlagiinae is still recovered within Dromaeosauridae, with a jackknife value of 83, whereas two internal nodes have support values of 63 and 84, respectively (Supplementary Material, Fig. S1). The bootstrap analysis found 48 unstable taxa including *Diuqin*, with an average support of 76.2 (slightly lower than the jackknife analysis). The final topology shows better resolution than that of the jackknife analysis, with Unenlagiine still recovered as a monophyletic group with a support value of 84. Internal nodes have bootstrap values of 57 and 83, respectively (Supplementary Material, Fig. S2).

The clade Unenlagiinae is supported by six synapomorphies: 152-1, preacetabular process of ilium more than two-thirds total ilium length (also present in *Alnashetri cerropoliciensis*, *Bambiraptor feinbergi*, *Saurornitholestes langstoni*, *Tianyuraptor ostromi*, *Xiaotingia zhengi*, and several Avialae); 154-1, reduced iliac supraacetabular crest (also present in several tyrannosauroids, alvarezsauroids, and therizinosaurs plus *Anchiornis huxleyi*, *Gobivenator mongoliensis*, and *Cathayornis* spp.); 160-1, cuppedicus fossa of ilium delimited by posteriorly extended ridge that reaches acetabular rim (also present in *Alnashetri*, *Tianyuraptor*, *Zhenyuanlong suni*, some derived dromaeosaurids, and several anchiornithines); 223-1, ilium with deeply concave dorsal rim of postacetabular process; 666-1, dorsal vertebral neural spines surpassing posterior articular surfaces of centra (also present also in several tyrannosaurines and parvicursorines plus *Alxasaurus elesitaiensis*, *Neimongosaurus yangi*, and *Microvenator celer*); 749-0, acromial process of scapula deeper than long (reversal from condition in other pennaraptorans).

#### Stratigraphic and morphological significance

**Fig. 8:**
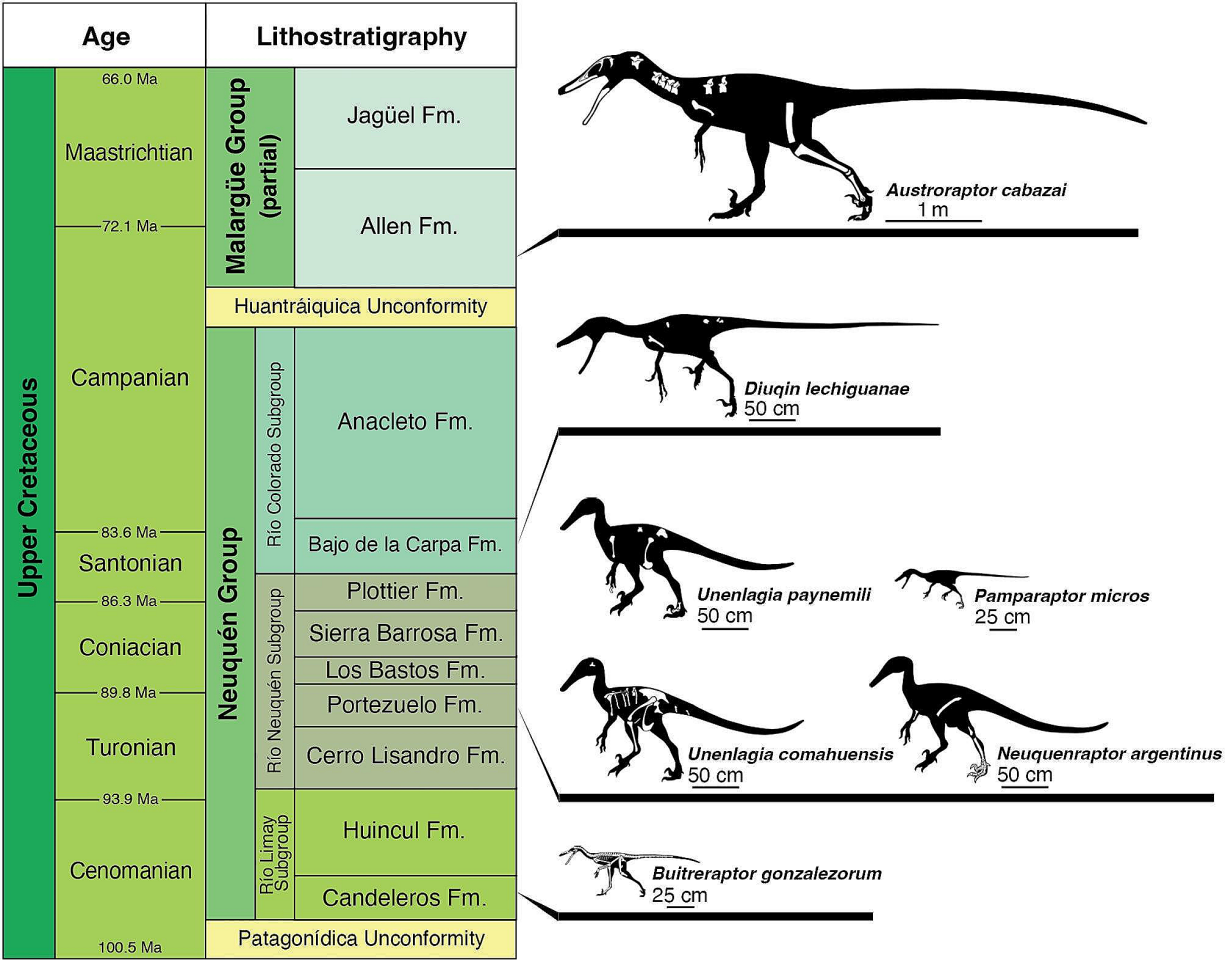
Stratigraphy of the Upper Cretaceous Neuquén Group (after Garrido [30]) indicating stratigraphic positions of definitive unenlagiine taxa (modified from Gianechini [57] and Gianechini & Apesteguía [127]). Skeletal reconstructions redrawn and/or modified from works by Scott Hartman (*Buitreraptor gonzalezorum*, *Austroraptor cabazai*), Gabriel Lio (*Unenlagia comahuensis*, *Neuquenraptor argentinus*, *Unenlagia paynemili*), and Jaime Headden (*Pamparaptor micros*, *Diuqin lechiguanae*), used with permission

*Diuqin lechiguanae* fills a significant temporal hiatus in the fossil record of Unenlagiinae in the Neuquén Basin of northern Patagonia, Argentina (Fig. 8). The geologically oldest definitive unenlagiine is *Buitreraptor gonzalezorum* from the Cenomanian (ca. 98 Ma according to International Chronostratigraphic Chart v. 2022/10 [126]) Candeleros Formation. The next-youngest undoubted unenlagiines are *Unenlagia comahuensis*, *Unenlagia paynemili*, *Neuquenraptor argentinus*, and *Pamparaptor micros* from the Turonian–Coniacian (ca. 90 Ma) Portezuelo Formation, although the potential unenlagiine *Overoraptor chimentoi* comes from the somewhat older (Cenomanian–Turonian, ca. 94 Ma) Huincul Formation. Prior to the discovery of *Diuqin*, the next-youngest unenlagiine was the exceptionally large-bodied form *Austroraptor cabazai* from the Campanian–Maastrichtian (ca. 72 Ma) Allen Formation (Fig. 8). Other possible unenlagiines from different outcrop areas, such as *Ypupiara lopai* from the Maastrichtian Marília Formation of Brazil, *Rahonavis ostromi* from the Maastrichtian Maevarano Formation of Madagascar, and *Imperobator antarcticus* from the Campanian–Maastrichtian Cape Lamb Member of the Snow Hill Island Formation of Antarctica, are of similar age. *Diuqin* comes from the Santonian (ca. 85 Ma) Bajo de la Carpa Formation of the Neuquén Basin, and thus fills a temporal gap of at least 15 Ma in the unenlagiine fossil record (conservatively, 90–75 Ma, possibly greater).

*Diuqin* also helps to close an important morphological gap in unenlagiine evolution as well. The new taxon from the Bajo de la Carpa Formation combines a humeral morphology that is most reminiscent of that of *Austroraptor* (e.g., deltopectoral crest oriented anteriorly; absence of medial sulcus between deltopectoral crest and humeral shaft; distal end strongly bowed anteriorly) (Fig. 6) with a probable body size comparable to those of earlier diverging relatives such as *Unenlagia* spp. (Fig. 6; Table 2). As such, the anatomy of *Diuqin* suggests that, over the course of unenlagiine evolution, humeral morphologies typical of *Austroraptor* antedated the appearance of increased overall body dimensions in these theropods.

### **Paleoecological implications of tooth marks**

Multiple examples of bite-induced punctures produced by terrestrial vertebrates such as theropods [128–131], crocodyliforms [132–136], and mammals [137, 138] have been documented in the fossil record. Two small, subconical tooth marks separated by ten millimeters are evident on the distolateral aspect of the *Diuqin lechiguanae* humerus. These traces are similar to those produced by animals with conical tooth crowns, such as many Upper Cretaceous South American crocodyliforms and two of the few unenlagiine species that preserve dentigerous bones: *Austroraptor cabazai* from the Campanian–Maastrichtian Allen Formation of Argentina [23] and *Ypupiara lopai* from the Maastrichtian Marília Formation of Brazil [2]. However, although crowns of both these unenlagiine species exhibit enamel ridges (i.e., ‘fluting’), they differ in morphology, with crowns of *Austroraptor* being more conical than those of *Ypupiara*, which are instead slightly more ziphodont [2, 23, 58]. The traces present on the distal humerus of MUCPv 1401 show inward-collapsed bone fragments with smooth surfaces, without any sign of furrows or crests that might have been made by enamel ridges. Moreover, several works [58, 83, 139] have proposed that unenlagiines might have been piscivorous, although Gianechini et al. [140] considered these paravians to have been primarily terrestrial predators.

Although many teeth of Unenlagiinae possess ridges and furrows, the apical part of the crown seems to have been smooth in these theropods [2, 58]; as such, we cannot discount the possibility that another unenlagiine individual, perhaps a member of the same species, produced the punctures seen on the *Diuqin* humerus. Similarly, we cannot refute the hypothesis that these marks were produced by the megaraptorid theropod to which the isolated tooth described above (MUCPv 1557) belonged. Megaraptorid premaxillary crowns are conical or D-shaped [94], which could leave a rounded depression after biting. However, conical tooth marks are also made by mammalian bites (e.g., [141]); indeed, mammals were among the proposed producers of several traces and conical punctures on the *Buitreraptor gonzalezorum* holotype MPCA 245 [60].

In sum, we consider the tooth marks on the *Diuqin* humerus to have most likely been made by a crocodyliform, mammal, or theropod, with the latter potentially corresponding to another unenlagiine individual (possibly even a conspecific). If the latter can ultimately be demonstrated (via, e.g., more detailed comparisons, morphometric analyses, and/or computed tomographic investigations of these tooth marks and known unenlagiine dentitions), then the *Diuqin* holotype MUCPv 1401 might constitute one of the very rare instances of cannibalism recorded among non-avian theropods. To date, among these dinosaurs, bite marks interpreted as the product of cannibalistic interactions have been documented only in *Allosaurus fragilis*, *Deinonychus antirrhopus*, *Majungasaurus crenatissimus*, and several tyrannosaurids [130, 131, 142–146].

## Conclusions

*Diuqin lechiguanae* is the first unenlagiine theropod to be discovered from the Upper Cretaceous (Santonian) Bajo de la Carpa Formation of the Neuquén Group of the Neuquén Basin of northwestern Patagonia, Argentina. The new taxon augments the fossil record of South American unenlagiines by filling a significant gap in their temporal distribution. Preserved elements of *Diuqin* show morphological differences from corresponding bones in other unenlagiine taxa, such as an accessory lamina on the posteriormost sacral vertebral neural arch, distinctive paired foramina on the posteriormost sacral and anterior caudal neural arches, and a humerus with a distally placed distolateral deltopectoral ridge and several conditions that appear intermediate between the humeri of *Unenlagia* spp. and the exceptionally large-bodied unenlagiine *Austroraptor cabazai*. Coupled with the multi-million-year stratigraphic gaps between *Diuqin* and geologically older and younger unenlagiines, respectively, these anatomical distinctions support the validity of the new taxon. Further, the humerus of the *Diuqin* type specimen exhibits two conical tooth marks that indicate that the carcass was fed upon by another tetrapod, likely a crocodyliform, mammal, or theropod (perhaps the megaraptorid represented by a tooth found at the same site, or even another unenlagiine individual, potentially a member of the same species).

### Electronic supplementary material

Below is the link to the electronic supplementary material.

Supplementary Material 1

## Data Availability

All data generated or analyzed during this study are included in this published article and its Supplementary Material file.
