# Supplementary material for: Diuqin lechiguanae gen. et sp. nov., a new unenlagiine (Theropoda: Paraves) from the Bajo de la Carpa Formation (Neuquén Group, Upper Cretaceous) of Neuquén Province, Patagonia, Argentina
Source: BMC Ecol Evol. 2024 Jun 14;24:77. doi: 10.1186/s12862-024-02247-w (PMC11177497; doi:10.1186/s12862-024-02247-w)
Supplement: Supplementary file 1 — Supplementary Material 1 [file 12862_2024_2247_MOESM1_ESM.docx]

**Supplementary Material for:**

***Diuqin lechiguanae*** **gen. et sp. nov., a new unenlagiine (Theropoda: Paraves) from the Bajo de la Carpa Formation (Neuquén Group, Upper Cretaceous) of Neuquén, Patagonia, Argentina**

Juan D. Porfiri, Mattia A. Baiano, Domenica D. dos Santos, Federico A. Gianechini, Michael Pittman, and Matthew C. Lamanna

**1. Phylogenetic data matrix**

Xread

884 167

*Allosaurus_fragilis* ?10000?00000001000110010001011001110110010??000000000000100000000001000100000000000101010010000000100101000000000001000000000????000000000000000?1000000000001000010010100000010001000001100000010000000000000000000100000000000?0?000?00110000100000010000111000210110000000002000000000?0000??0000000?00000??000000?010000[01]0?0????????0000020?00?0000000101000?0?0010000?00000000000001000010?01000??0000000?000?000002000000000000?00000?00?0010000000020000?000100??001100000[01]0?00000000100000000110000000000100011000000021121001001000100?1????000000000100??10?0000?00000000000011000?100?000?0?1000000000????000?00010010000101000000000000000102000000000000000000010000000000020101[01]00001000000000000000000100000010?00010000010201100000000011000000001000000000000001001000100000000000000000000000000000000000000000000000??000000000000000000000?000001000000000000000?0000000000000?000000100????????

*Sinraptor_dongi* ?10000?000?0001000100000001011000010110010?00?000000000010000?00000100?10?000000000101010010000000100101000000000001??????0??1?10?0?0??00??0??????000000000?010??01001010000001?0?1000001100000010000000000000?000?000?00?000000?0?000?001100001001000100000100002101???00000000000000000?0000??0000000?00000??000000?010000???00?00??00?????2????????????1010???????????????????????????????????1??????0??000??000000002000000000000000000?00?00??0???00020000?0001?0??0011000??????????00010000000000000000001000001100000002102100000?010?00?10000000000000100??01?0000?00000000000?11000?100?010?0?100?00?001000?0010000100100001010?0?00000000000112000000?00????00000010000?00000020101??0??1000000????????????000010010?0000?000010201101010?00011000000??1?00000000000001001000??0???000000000100?00000000000000???????00000000??000000000000000000000000?0010?00000?00000001???1?10000?0?0000000010????????

*Dromaeosaurus_albertensis* ?0??001000000000010??0???0?01??01110????1111????101?1001100??00000010011111000?0000101001?????????????????????????????????????????????????????????????????1??????????????????????????????????????????11?1?0????00??????00?0?00??1?????????1?00??011000100?0???????????????0???0000?00000??0000??0000000?00000??00?????????????????????????????????????????????????????????????????????????????????00??????????????????????????????????????0????0?????????010121?000??0???00?0?????????0????0??0??0???0??????0?0100?00?00????????????????????????0????00000100000???????0???????0001001?0?0????00??00?????0?0?10???????????000?1000000010????00000000001120100000000?0?00010??????????????????????????????????????????????????????????????????????????0??000?0?????????????0??0?010????????????????????????0200000????????????????????????????????????0?1?0??110????????????????000?0??00???????????0??????20????????

*Deinonychus_antirrhopus* ?0010????1???????1??0000?0111100111000111111100?1??00001110100000?0100111?1000?0100101000?110001100?1121011????00110111121??????1?1101110010011100000100221110101020220?01111211201?1111000000000100011010010000000001100000?00010?0010002110000011110110000??00000122000000000?0????000????0???????????00000??000000?00000?[01]0?0?????????????20000?000?000111000?0?0010000?0000000000000101?0???00000??0100000?000?000000000000000000000000000?00100000000????????0?1????00111?????????0000?????00001?10?0010001?000001010??00?0???????00??0?0??0?????00001000000??01?00?0?00000001??????????????????????0?0?1001000?000?00?????????????????00000000001120100?0000?0?????00000000?00001000100?????00000?1101010000100??1?0000?????????????????00000?000000000?00?100?00??0001?1??0?000010?100000000010?010??00?000000000000000000000000??0????000000000100??1101?000110???200?10001?0000[01]01010200100?0?10120???????[12]

*Velociraptor_mongoliensis* ?001001001000012011200001011100011100012111?10?0101000011101000000010011111000101001010000110001100111210111110001101111211110101111111100100111000001002211101110202202011112212011111?000000000111011010010000000001100000000011?00101021100000111001100000000000112000000000000?000000?0000??0000000??0000??0000?0?000000[01]0?00??000?00000020000?000?000111000?0?001000???00000000000010100???00000??0100000?0000000000000000000000000000000?0110000000010[12]21?0001101100011100000?100000000?0100001?10?0010001?0?00010101000000???0??0?01??00?0????000001000000??01?0000?00000001001?0?0???000??00110100?0?0?01000?000?000001001000?00000000000??00?112010000???????00000??????????????01001000?000001110101?0?0?00?0100000110?01?0100000000000000000000000?000100000?000?101010?0000100100000000010?01002000000000000000000000000000??0000100000000010000?1?10?00110100200010001000101010100001?000110120?[1 2]??????

*Balaur_bondoc* ????????????????????????????????????????????????????????????????????????????????????????????????????1?2??1????0???10?????????0????111[01]11101001?1?00001???2???0111?202?02?0?1?22122????????????????2110001001000??00??1???????????0?00??0??????????????????????1?????2?00???????????????????????????????????????????00?00000?[01]???0????????????20??????0???0111000?0?001000???010?00?????010?00???21??0?00000020?100??0000??????00???0?000000000?00??0?00000????????010????????1????????00000??????????????????????????????????????????????????????????????????????????????????????????????????????????????????????????????????????????????????????????????????????????????????????????????0100?0?0??0?00?1101010000?00?01000?0110?01?????????????000?00??00?????00100000?00??1??????0000100100?0000?????0?0???????????000000000000000000??000????000000210????????000?10???200?1?????????1?10??200????001?0??????????

*Tsaagan_mangas* ?00100100100001201120000101010001?1000121111100010110000100??000000100?11110001010010100001100??1??????0?????????????????1????????11?111???????????????????????????????????????????????????????????????????????00??00??00?0000???????????2110000011?00?0000000?00001????0000000????000000?0000??0000000?00000???000??????????????????????????20000???0?0?0??10????????????????????????????????????????????????????????????????????????????????????0????????0121?00???0??00011?0????????????00?0100001?10?0010001?0?00010101000?00???0??0?01??00?0????000001000000??0??0000?00000001001?000???000??001101?0?0?0?????????????0001001000000000000000??00?112010000000????000000???00?00001??????????????00?110???????????????????????????????????????????????000?000?????????0?10?010?????10?????????????????02?0000000?????????????????????????????????????000?1?1????110????0???000?0??10???????????00?????20????????

*Bambiraptor_feinbergi*

?0010??001000012010[02]00?010111000?1100012111?1000101?00011?0?0?000?0100?1111000?010010100??1110?1100?1??100?0?1100[01]1011??2????0101011011110?001?100000111?211?0101?202202?111022100???11100000000010001101?000000000001100000000011?0010?021100??11?110??0000?000000122000?00000?0??00000??0000??0000000?1?000??000000?00100??0??0?0?0?0?0000020000?00000?011?000?0?0?1000??000000000?00010??????00000??0000000?000?000000000000000000000000?00?011?100000000121?000110??00011101100?10000000010100001?10?0110001?0000010001000000???0??0??1??00?0????000001000000??01?0000?00000001??1?0?????000??001101???????????????????0????????????????0?000??00?1120100?????????????0000000?000010001011000?000001110101000010?00100000110?01?010000000?00000?000000000?0001?000??00001?10?0?100010?100????????????????0?000?????00??00????000000??00001000000000100?0???10000110100200?10001??00010101100110100?000?0????????

*Tianyuraptor_ostromi* ??01?????????????????0????1111????????????0??1???????????????????????????????0?0?0?1010??0???0?????????0?0?0?1??01?0112???????1??00?011110?001?1100001112?1??0?1102223?21111022?0???1?110??????????00?11????000??0???1??00?0??01???1011?0???0??????1?0??????????0????2?000?0??????????????????????????????????????0??????????0?00?00????00000?0?00?000?0?0??????????????????????????????????????????????????00?1?0????10??????????????0?0????????20?101????????????1???????????0000?10??000??1???????????????????????????0??????????????????????????????????????????????????????????????????????????????????????????????????????????????????0?????????????????????????????0???????????????????????????01?1?1???00??0?0????000???????????????0?????0?0???00????0????0?0???0???????0???0?????0??0??????????????????0?????0???0?????????????????????????0010????????0?0???????????0?0???0?0101011?0??1?001112?012?0000?

*Sinornithosaurus_millenii* 0001??????0??????????00???1110????1000111100?1????1??0????????00?00100??1????010100??100????????1??1???????0???00?1??1???????01?1?11011110?0?????0000?00?201?01?112023?2?111022?2?1???1?????????110001?1100?0?00010001??0000???01101111002000000111100?00?00???000???20000?000?????0??000????0????00000??1000??00?00???????0?0??0???????00?0?[12]0000???0?000100?00?0??010?0??00????????????????????0000??0000?0000??0?0?00?????000??0?0000?00?0???0111?11000????????01??10000111?1100?1000000?010100000000?0000000?0?00000001000200???0??0??1??00?0????000000000000??00?0000?00000001??1????0??000??001101?????????0??????????????????????????001000?010112010000???????100?0??????????????????????????0011101????00?000?????????0?01???????????????0?0???00000??0??00000?000?1??0?0?????10?????0?00?????0?0???0100??????0????00000????001?000?????0??000100?011?????0110????0???000???00010111120?11?0??101201101000?

*Microraptor_zhaoianus* 0??????????????????100?????????????????????????????????????????0??010?0?1????0?01000000?????001?1??01?2100?01???0110?1212111?01?101111111010??111000011012111??0112023?2?1110221201?11111??00?0001110111110?100??000?11?000????1010111100?0?0???1?110???0?00??0?00???20??0?000???????????????????????????0?0???????00?01000?[01]0?00?0000?001000200???0?000?01???10?0???10100?000??1?0?000?????????000???????0?00?0000000000????000?00??000000000??1211111??00?021???011011???1?1?1100?1000000??????????????????????????????0?????????????????????????????????????????????????????????????????????????????????????????????????????????????????????00???1?????????????????????00?????????????00001????????01???1?10000?000???0000??0?01??10?????0?00000?0???00????0????0?00??0????1????0000???10??0?00???0?010???????0???0?00??000??0?000000?000?10?000000010??????????0?1??0?????1???1??000101?112???0?????01?01211110?

*Zhenyuanlong_suni* 10?????????????????10000??1111?0??10001?110011????????????????00??0100?11??0?0100001010000???0?11???????00?1????01?0?1????01?01?????0???1??0???110000110221???111011?3010111?21120??????0??0???????00??1?10?00000?00????0?0???00??00011?01000000111?00?1000??0000??1?20000?000??????????????????????0????0?0????0?0?0?????0?0??00????10???????????????????00??00????0?0?????????????????????010?10?0???00000?00?0??0?000??????0????00?000?0?0???01??110000?????????11?110?0????????????0?????????????????????????????????????????????????????????????????????????????????????????????????????????????????????????????????????????????????????????????????????????????????????????????????????????????????????????????????????????????????????????????????????????????????????????????????????????????????????????????????????????????????????????????????????????????????????????????000101011?0?1??00?1???01[1 2]?0000?

*Changyuraptor_yangi* ??????????????????????????????????????????????????????????????000???????1????00000?1?10??1????????0?????????????02???12???11?????0???11?1??00??1000001???????????????????????21?2???????????????????0?011???1?0??000????0????????1?1????0??????????100?0?????????????2??????????????????????????????????????????????0?????0??0?0????????00??0????????????????????????????????????????????????????????????0??????????????????????????????????2???11?11111??????????0???11???????1100??10??????????????????????????????????????????????????????????????????????????????????????????????????????????????????????????????????????????????????????????????????????????????????????????????????????????????????????????????????????????????????????????????????????????????????????????????????????????????????????????????????????????????????????????????????????????????????????????????0?0?01?11?????????1????1211110?

*Graciliraptor_lujiatunensis* ???????????????????????????????????????????????????????????????????????????????020???1??????????????????????????0???11?12????????????????01011?1100001?????????????????????????????????????0??0?010?[01]11?1??0??0??00?????????????0???????????????????00???????????????200??????????????????????????????????????????????????????????????????????????????????????0??????10?0???000??0???????????10?0000??00?000??????????????????????????????????????????10????????????????????????????????000???????????????????????????????????????????????????????????????????????????????????????????????????????????????????????????????????????????????????????????????????????????????0????????????????????????????????1????00?00????????????????????????????0000????00??????100??0??0???????0?????????00?0?00?????01??????????????????0???00????????????????0?000?10????????0?0?????????????????????01???20?1?????????0????????

*Hesperonychus_elizabethae* ?????????????????????????????????????????????????????????????????????????????????????????????????????????????????????????????????????????????????????????21??010?????????????221211?????????????????????1????????????1??????????0??1??????????????????????????????????????????????????????????????????????????????????????????????????????????????????????????????????????????????????????????????????????????????????????????????????????????????????????????????0???????????????????????????????????????????????????????????????????????????????????????????????????????????????????????????????????????????????????????????????????????????????????????????????????????????????????????????????????????????????????01000?0110?01?????????????????????????????????0???0?????1????????????????????????????????????????????????????000?????????????????????????????????????????????????????1??2??????01101??????????

*Pyroraptor_olympius* ?????????????????????????????????????????????????????????????????????????????????????????????????????????0????????1????????????????????????001??????????????????????????????????????????????????????01??1?????????0?????????????0??????0???????????????????????????????????????????????????????????????????????????????????????????????????????????????????????????????????????????????010??0????????????????????????????????????????????????????????0????????????????????????????????????????????????????????????????????????????????????????????????????????????????????????????????????????????????????????????????????????????????????????????????????????????????????????????????????????????0?0??????????0?????????????????????????????????????????0???????????????0?????????????????0????????????????????????????????????????????????????????????????????????????????????????????????????????????????????????

*Rahonavis_ostromi* ??????????????????????????????????????????????????????????????????????????????????0???????????????011121???1?01?011112?12???????????0??11??011??????0?01111?10111120?3?2012101?121??21110001000?011101101?0100????0??1?????0??10001011111?????????????????????????????????????????????????????????????????????????????011000[01]0???????????????2????????????1100??????????????????1??????010100???????????????000000?000000000100000000?00000000?001??01???0????????0?1?11?????1????????00?????????????????????????????????????????????????????????????????????????????????????????????????????????????????????????????????????????????????????????????????????????????????????????????????100?1??0?0??0011??????0?????0?100000????01??10???0?0????00?0???00???????????00??0?00?1????0000??0?0???????0???010???????????000?????????0000000?000?10??00?0001????????00?0?11?0??0????????????1??0110???0??00102??????????

*Buitreraptor_gonzalezorum* ?0010?????????????????0??011?001?????????000?00??01?0?????????00??001???0??????0210??100?001011110011??110???100011000[0 1 2]121???????01101111010?1?1000001?1?1111011112[0 2]230201?10121200??11100?0?000011?01111?0000011?00?1??0?????1101000110011000???0??00???00???0?00?11200?0??0???????????0???????0?000101???0?????00000010000[01]0??????????01000100?0?0?00??0?10000?0?001100??00100100000001?100???0100????00000000???000000?00????????0?00000000?0010?001100????????0?1????00101?1100?10000000010????????1?11?00?0??0010?0101?0??0???????????????????????0000???0?0??0??0???????0??????100?????000??00?1?????????????????????0????????????????0[01]000???0?1?2????????????????000??1????0001001000??00?000001110101000??0000100000??0??1?0100000?000000000?01000?0?000?00000??0?00?1??0?1000100?0??00?0?0100010???0?000100000000000000000000???000100000000010??????10?00?11100100010001??2111010110001?1?1100021????????

'*Neuquenraptor_argentinus*+*Unenlagia_*spp.' ????????????????????????????????????????????????????????????????????????????????????????????????????11211111111???1????1????????????0??1001???????????01111?1011112022020111?121202011?10000??0?010??111100000????0??11????0??110110?110??????????????????????0?????220????????????????????????????????????????????0??0000?0?????????????????1????????????1100?0?0?0011000?00100????????????0???????????????00000?0000000000000000000?00000000?00????????????????????????????????????????????????????????????????????????????????????????????????????????????????????????????????????????????????????????????????????????????????????????????????????????????????????????????????????????11011?0?????0011???01???????00100000110?01?010000000?00?0100???00??????????000?00?00?1????10????01???????00???010?????????????00???0????000000??000010??0?000010?????????0??111001000??????????1??0110???1??01[0 1][0 1]1??????????

*Austroraptor_cabazai* ?0000???????????????????2?100????????12?0001????????????????????0?011??????????0210??10??????0111001???111???????????????????????????????00????????0?0?????????????????????????????????1??????0?010?01?[12]1??????????0????0???????0???????111?00??01??00?????????????0[12]20??0?0??????????????????????????????????????0??00?00????????????????????????????????????00?0?001100???01??0000000???10??????00???????????????????????????????????????????0?????0????????????0?????????????????????000?0??????????0?10100?0000010??1???????1???0????01??00?????????????????0??01?01????????????????????0?10?00????????????00000?000?00?????????????????0??0010000???????????????????000???????0001001101??????????????101?0?0??????????????????????????0?00??00????00?00??????0??0??0?????0?0???????????????0???????0???0?0001000?????0?0????????????????00?????00?????01?1?0??1?????1?0??0001??2111???1?0?????1??????0????????

*Shanag_ashile* ???????????????????1??1010110?????????????????????????????????00??011??????????01001011????????????????????????????????????????????????????????????????????????????????????????????????????????????????????????????0????0????????????????21100001???00???????????????????00??0?????????????????????????????????????????????????????????????????????????????????????????????????????????????????????????????????????????????????????????????????????????????????????????????????????????????????????????1?10???00?0?000000???????????????????????????????????????????????????????????????????????????????????????????????????????????????????0??00??00????????????????????10???????????????????????????????????????????????????????????????????????????????0?0????????????????????0?????????????????????????????00????????????????????????????????????????????1?????????????????00??????????????????????????0????????

*Mahakala_omnogovae* ???????????????101??????????????????????1?00???????0?0011??????????????????????02????1???00??0?11?011?211??1?100011012210????????????????00??1110000?100?211101?2??????????????????0111100????0?011001101?00000??00??11?0?????0000?????00????????0????????????0????1[0 1]2?0??????0?????????????????0??1?00???????????000?010000[0 1]0????????????????????????????10??[0 1]0???0?0000???0?????????????100????0??0???000000??0??001??0???000000000?00000000?001??00?000????1???0??????011?0????????000000???????????????????0???0???????????????????????????????????????????????????????????????????0?????000?00????????0???????????????00010?????????????????????0????????????????????0000?????0?01?000???????000??????????00????00100000???????0100000?0?000?100?0?000?0????0?0?0???0????1?10???00???????????0????010?20????0?0??0???????000000000??0??0100000000010?0????1?000???10????01????0????1???11?00????1??00??????????

*Atrociraptor_marshalli* ???????????????????[02]00?0??1010????????????????????????????????00??010????????0?0000101001??????????????????????????????????????????????????????????????????????????????????????????????????????????????????????????0????0????????????????21?00??0??111?????????1???????????0??????????????????????????????0????0????????????????????????????????????????????????????????????????????????????????????????????????????????????????????????????????????????????????????????????????????????????????00001?1????1???0?0?000?01???????????????????????????????????????????????????????????????????????????????????????????????????????????????????0?????0?00????????????????????0???????????????????????????????????????????????????????????????????????????????0?0?????????????0??????0????????????????????????????000????????????????????????????????????????0??1??????????????????00??????0???????????????????0????????

*Utahraptor_ostrommaysi* ???????????????????[02]00?0??????????0?001??????????????????????????????????????0????0101??1????0?1100?1??1????????0??011?????????????101?1???????????0?????????0?01????????????11?????0111?00??0?001000100???10????????11??0???????0?????0???????????1???0???????0???10???0???0?????????????????????????????????????????0000???????????????????20000?000000???????????????????????????????????????????????????00???????0??0000000000000?0000?????0?10??????????????????????????????????????00?????000001??????????????????????????1???0??00010?0????????????????????????????????????????????????????????????????????????????????????????????????????????????????????????????????????????????????????????????????????????????????????????????????01?????????0?0?????????????0?????0????????????????00????????????0???????????????????????????????0?0???????????????????1???????????????????????????????1???????????????

*Adasaurus_mongoliensis* ?0010?????0????2??????????????0?????0?1??11?1?0?101??001??0?????0????0?2???0?????????????01100?11?0111?10?111[01]100??011?1?1??????1?111111??????????????1022111010102?2202?1?11221001?111000000?000?11010010010??00??00110?0?0?0001??00100?????????11????????00?0????1??????????0?????????00??00???000?0???????????000??010000[01]0???????????????200?0???00??01110??????????????????????????????????????????????00?000000000000000000000000000?002??11?00???00???21?000??0??0001?1?????????0???0??0?????????????????????????????????0?????????1??00??????0000010??000??0??00?0?0?00????????0??????????0????????????????????????0??????????????????0???????11201????????????????????????????????????????????????????????????100000??0?01???????????????0?0???00?0????????000?00????10?????????????????????????00??????0??00???????????000000??000010000??00010??????????01??????????????????????0??????0?101101??????????

*Achillobator_giganticus* ?????????????????????????01?1??????????????????????????????????????????????????0000101??????0?01100?11210???????0??011?11??????????101????????????00??10220??11010102102?011011?001?21110?0????00?000?101????0???????1???0????001??00100021100??0??100????????0???????????????????????????????????????????????????????000????????????????????20000?0?0?0?0??10??????????????????????????????????????????????00???0?00000??000???????0?0000????????0???????????????0??????????0???????????00???????????00?001???0?0?000??1????????????????????????????????????????????????????????????????????????????????????????????????????????????????????????????????????????????????10??????????????0100??????????????????????????100000??0101?0100000?1000??????????0?????????0??01?????1??0??0??????????????01??0?????????0??00?????????0000000???0000100???????1???????????????1?1?????0?00????00??0??????0??0?111?0????????

*Saurornitholestes_langstoni* ?????????????????????????????????????????111????????????11????0?????????????????100101?00?11000110011121011011100?1011?1?1????????????111?????????000111221?1?1?1?2?0?????????????????11??0????00??001101??00???00?0?1??00?0000010?001000????????1110011??????0????1220???????0????????????????????0000???????????0????????????????????????????????0???00??1????????????????????????????????????????????????0?????????????????????????????????????0???00??????????0??????00111????????00?00???????????00?01??0?0?0?000?00????????????????????????????????????????????????????????????????????000??00?1???0?000??????????????????????????????0??0000000????????????????????0???????????????1?0???????????????????????????????????????????????????????????????0??????0????????????????0???????????????1??01????????????0?????????00???????????????????????????11??????????????????0???????1?????????????????20????????

*Saurornithoides_mongoliensis* ?00??1?1??1101???0?110001?1000????????2?????????????????1?010?100?0010??1????0001110101?????????1???0??1?????100??1??????????????????????????????????????????????02022020?010??10[01]1?11110???????????0???10?01??????0??1?0???????????010??11000??0?1?01?0?1???0?000??????0??0000????0?1000?0??0???????????0000???0?????0100?0????????????????????????????????????????????????????????????????????????????????00????????0?0????00000000000000?20?02100000000000201001?10???1110?1????????????00?0100000??2??11?0?0?1?0100010100??00???0??0?????0??0?????????????00????????????????????????????????????????0???????1????????000????????????????0[01]????????????????0???????00000??????????????0????????0?0?????????????????????????????1?010???????????00????00000????????00??00????0?0???00???????????1??????0??000?0?????0???????????????????0?00???????0?101?0????????1???0??????0?0?00?001??0??0?0???0??????0????????

*Zanabazar_junior* ?00101?12?110100?001?000??100000????2022000?21??0??11100??????100?001???1????000111010100??????????????????1?1000?1020?1??????????????????????????????????????????2?????????????????????????????011????2???????????00??00???11????????0??110000000100000?1??1??00???????00?000?????001??0?00??????1??????0000??0?00????????0[01]0???????????????????????????????????????????????????????????????????????????????????????????????00010000??000??2????1??0???00000201001?11???1110?1????????????00??100000??2??11?0?0?1?0100010100?000???0??0?????0??0?????????10??000??00?0??????????????????????000?00011010??????????????????000?00000????0000???000?001???????????0????00000???????????????????0??????????????????????????????????0?????????????0?0????????000?????????????0?????00???0?????????????????01?00?0000????????????????????????????????0?00????100100???????????1????000?0??00??????????????????10????????

*Xixiasaurus_henanensis* ???????????????????01000101110??????2?22????????????????????0?00??001????????000201??01?0?????????????????????????????????????????????????????????00?1????????????????????????????????????????????????????????0???00?????????????????????1?000000???01?0000????00???????000000???00????????????????????????0?????????????????????????????????????????????????????????????????????????????????????0??????0????????????????????????????????????????????0?0?????????????????????????????????00????110100??2?01100?0?1?01000101000000???0??0?????????????????????????????????????????????????????000??0????????????????????????????????????????????010?000????????????????00000???????????????????????????????????????????????????????????????????????????????000??????0??????0????0?0??????????????0????????????0100???????????????0????????????????????????1?010??????1??????????000????00????????????0???????????????

*Byronosaurus_jaffei* ?????101???101?1100110001011?0??????20220??????????1?100??????0000001??11????000211??01?0?0???????010121????????0??02????????????????????????????????????????????????????????????????1????0?0??0???????21??????????0????0????1???????????11000000?1?00????0??0?000??0???000000????????????0??????????????0?0????0?????010?????????????????????????????????????????????????????????????????????????????????????????????????000?????????????????????????????000200011??0????????1????????????00??100000??2?011?0?0?1?0100010100??00???0??0?????0??0?????????????00????????????????????????????0???????????0??????????????????00?000000????????0??00??00?????????0???????0?0000??????????1???????????????????????????????????????????????????????????????????000?????????????0????000????????????????????????00?0100?????????????????????????????00?????????1?0?0?1????1??????????000?0??001???????????0??????0????????

*Sinornithoides_youngi*

?0??01?????????????1?000??1??0????00???2???????1??????????????00??0010???????0001110?01??????11??001???????????0011?102121011???1?00?1?101?0???100000????21??01??02??30???11?0??001??1110?????0???1?000110001?0??0?0?1??00?0???0???0010?0??00000????0??0??0???00???0?2000000?0????????????????????00?????1?00??000??????????[01]0?0????????0??0?20??0?00000?0??1000???0?1000???00?0?????????0??010?0000???000000??00??0??000????00000000000000020?02100000000????????101??????1?010100??1??000?????????????????????????????????????0????????????????????????????????????????????????????????????000??0??????????????????????????????????????????[01]????????????????????????0?0?00?????????0???????????????00?110101?000?000???????????????1??????0?????000???00?0???0???0?00??0??1????0?????100????0?0????????0???000?000???00??10?000?????0??00???00000000010????0?1?0?0101????0????0????000?0101100?2??00??0??0????????

*Mei_long* ?0????????0????????11010?????0?11?002012100001?1010??100??????00010010??1?0000?021???01?0011011100011?2110?01???0110222111?1?????111?11100?0?1?1000001?012111?1??1200302?1110221?[01]?0111100?0101001000??110011?0?0000???00000??000100?1??0?1?000000?001?0?0000?000001020000?000?????????00?0??0??00??0101????????0?000?010000[01]0?0????????0100020?00?00000?0111000?0?0000000?0000000?000?01?1?0???00000????00000?00000010000??00000000000?000021?01100000??0???20???1010??101100?1100?10?0000?110??0??0??????????0?1?01????0?????00???????????????0?????????????00??????????????????1???0??????0????0????0????????????????????????????????????0?000?????????????0???????0?0000?????????0?????????????????????1?1?0????????????????????????????0??????00???00?00?0??????0????0????1?0?000?1001??00?0001?0?010???0???0?0000000000?000????00???0???00?000000100?????????01??????????0?0???0001???1??0????00?????0????????

*Jinfengopteryx_elegans* ?0?????????????????1?01???2000????????1??00?0????1???100??????00?00??0??1??0?000?1???0???1??????????????????????02????2?200???????1101110??????100000?????1??????????????????2??0??????????0?????????????????0?0??00????00??????????????01?000001???0??0?0??????0????200?0?000???????????????????????????0??????0?000???0?0??0?0??????????????00?0???0?0?01???0??????????????????????????????????????????????????????????????????????????????????10?0000???????????0??11?01?????????????000?110?????0?01??1????0?????????0?????00?????????????????????????????0??????????????????????????????0????0????0????????????????????????????????????0??00??????????????????????????0?????????????0????????0????1??0????00????0??????????????????????0???????????????0?0??00??????00????1?0???0????????0????????????????000??????????????0?0???????00?????????001?0?????????????????????000???00??01011?0????0??????0?10??10?

*Anchiornis_huxleyi* 000100?????????????10010??11100?????0?2?000??1???11???????????000??01??1?????00020???01??0?101???00?0??010?01???01101220??01?????011111110000??100000010?11?10011020?202?1?1122121???1?1?000??1?01?000121100010?1000?1??00?0???????0?11?011000001???0??00?????000???030000?000??????????0??????0????0??????0??????000?0?0000[01]0?0????????0000020000?0?00??0?10000?????10?0???0????????????????10?01000??0?00?00??00?00000????????????00000?0?00??0100000000????????00??1110?1?1?1100010??000?110??0??0?01?010?0?0?1??101?00??0??00???????????????0?????????1???000??0???2??????????1?????????00????0????0????????????????????????????????????02000??01?????????0???????0?0000?????????0???000??????000??????1???00??0000100000??0?01?????????0?????0?0???00?00?0??100?00??00???11?0?0000???????0??????????0????1000????0????0??0?0000000???00?0???????00100???0???0?01??????????000?????????????????????????11101110?

*Xiaotingia_zhengi* ?00?????????????????1?1???111000????0?2??????????11????????????001001??1???00??020???01??0??0???1?0?????10?01?????1??????10??????01?01111?10???1000001011?1110?1102122?????102?12[01]??????????10????????1?010001010000?1??0?10??00???0010?011000??1???00??0?????000???22000??000?????????????????????0000?????????0??00??10000???0????????010002????????????11?000?0?00???0???0?0?0????0???????????0100??1?00000???0?0000???????????????????0??0?02????10000????????00??1??????1?1100?10??000?110??0??0?01??1????0?????0???0?????0????????????????0?????????1???00?????????????????????????????0????0????0????????????????????????????????????0??00???1?????????0???????????00?????????0???????????????0011??????0???000010?000???????????????0???????0???00000?0??100?00??00?1??1?0???????0????0??????????0?????000?????0???0??000?000?????0??????????0?100???0?????0101????0???000???01111101120000100?100??????1???

*Aurornis_xui* ?0010??????????????0101???11100???000?1?0??????????????????????00?001????????0?020????1??0????????0????????0????0??0?221?00??????01?01111??0???10000??001?011????02122020??10???????????????????????0??100000?000000?1??0??0??00????111?011?0000????0???000???0000???20000?000???????????????????????????0??????000??????000?0??????????00???2??0????????0?0?010????????0????????????????????10?0??00??0?00?00?0000000????????????????0???0??????1??010000????????0???1?1?1??1??1000100?000?110??0??0?01??10???0?1??101?001???000???????????????0?????????10??000??0??02???????00???????????00????0?????????????????????????????????????????02??0???1?????????0??????????00???????????????????????0???01???1???00??0000100000???????????????0???????0???00??0?0??100?00??00????1?0???0????????0??????????0?????00??????0??????0?000000???????????????00100???????????0?????????00????0???0101?00?0??00??????????????

*Eosinopteryx_brevipenna* 00010??????????????1?01??????000????0?1?0?????0??1?????????????00?001????????00020????1??0????????0?????????????0??0?231?10???????1101111??0???100000?001?1?1??1?02002?20??10???????????????????????00?1??0001000000?1??0??0??00????011?0???00??????00?0?00???0000???20000?0000??????????????????????????0??????0000???10000?0?0????????00???2??0????0???0?1?010????????0???????????????????????0??00??0?00?00?000?000????????????????0?????00??01???000??????????0???11??1??1??1??0??0?000?11???0??0????????????????????0?????00???????????????0?????????10??0??????????????????????????????0????0?????????????????????????????????????????0???0???1?????????0???????????0??????????????000??????0??0011??1???0???000?100000???????????????0?????0?0???00??0?0??100??0??0??1????0?0?0????????0??????????0????100??????0??????00000000???????????????00100?????????0?01????0????0????????????????????????????101000?

*Troodon_formosus ???1?*1112?1101000001???0?011?0??????20220000210?0??01100????0?10??001??????????0111010100???1111100101211111?1000?1020??11?????????????1?010?????000010??????????0?022?20?11001?0??01111000???00010000021??01????000??1?0??010???1??0??0?1?00000?0?0???????00??????????????????????001??0?000?????1??1?1?????????00???????????????????????????????????????????????????????????????????????????????????????????????????????????????????????????????0?????00000201001??0???1110?1?????????0000??????????????????????????????????????????????????????????????????????????????????????????0?????0000??00110????????????????????0000001000??000000??0000000????????????????????00???????0?0??00?????????????????????0?????????????????????????????????00000?000??0????????00??0???0??0????00??01??0000?0?1??01?01?00?0????????????0?0?????????????????0?000?10?0?10?0?0?0????????????0??0???????????????????????????????1

*Sinovenator_changii* ?0???0002?000011110010101?1110?011?02??2??0011???1?10100????0?000?001??????0000011110?1?????11?10100012110?001000110222?1?????????110111???????100?0???0?211?010112[0 2]0302?1110221201?11110000??10011000111??110?000000???0000110001000110011000??1??011??0000?00000??0??00000000000?000000?00?0??0000010100[1 2]00??00000??010000?????????????????20000??00?0101?10??????????????????????????????????????????????00?1000000100000000000000?0?00?020?0110?00000000020?010?10??1011000???????000000?????0000?02?011??00?1?0100010?????0??????????????????????????????????????????????????????0????????0?00????1???????????????????0????????0???00000??00???0????????????????????00?????????????00????0??????00111?????????????0000?0??0?01???????1??0????0?0?0???0?0???????????0?0?1?1??0?????100????????0????0??01?01?0?0000?00??????0?00??0???0000?0000000001?1???0?????0??1??1?????000?0????????????????????????????????

'EK_troodontid_IGM_100/44' ?????0012???????????????????????????????????????????0???????????????1??11??????????1??????0????????????????????????????????????????????????????1?00000???????????????????????????????????????????????01?1?001???00??????0?0??1?????????0?????????????????????????????????????????????????????????????1?1??????????????????????????????????????????????????????????????????????????????????????????????????????????????????????????????????????????????????????????????????????0?????????000???????????????????????????????????????????????????????????????????????????????????????????0?????????????????????????????????????????????????????????????????????????????????????????????????????????????????????????????????????????????????????????????????????????????????????????????????????????0??????????1????0????????????0000????????????????????0?1????????????????????????????????????????????????????????????

'IGM_100/1126'

?0????002?0000?11[12]01?010??2000?011?020121000110?01010100??0??000000000?11?000000211??00?0??????????????????0????01102???????????????????????0??1000001???211?010?020020200010131012??111000????0??2?001210001?00000011?00?0?020000?00100?1?00000111?01?0?00?000003??????000000?????00??00?00?0??0??0000?00?00???0?0?????????0????????????????????????????????????????????????????????????0?00???0000???0100000?00??0???0?00?00?????00?1?000?20?02????0000001020?111??0??101?001???????0?000?????????????????????????????????????????????????????????????????????????????????????????????????????????????????????????????????????????????????????????????????????????????????????????????????????????????????????????????????????????????????????????????????????????????????????????????????????????????????????????????????????????????????????????????????????????????????????????????????????????????????????????

*Almas_ukhaa* ?00???????0???11???1?000??20000?1??020121?00?1??0?0??????00?0?000?0????1??00000021???0000???????????????????????011???????????????????????????????????00??1??????020?20200[1 2]102?1011???????00???0????0??2???01??00??0????0?0???00?0?00100?1?000000?1?01?0?00?00?003??????0000000????00?000?00?0?????0000?0?000??0?00?????????????????????????????????????????????????????????????????????????????????????????00?????00?00?0?00?????????1000??2????????????????????????????01?????????????????????????????????????????????????????????????????????????????????????????????????????????????????????????????????????????????????????????????????????????????????????????????????????????????????????????????????????????????????????????????????????????????????????????????????????????????????????????????????????????????????????????????????????????????????????????????????????????????????????????????????????????

*Archaeopteryx_lithographica* 100?0000??000??112010010??1110?011000012100?10?0000??100111?0?000001000002?00000200??00100?1?1???00?0??1?0?0???0021012311000????1011111111000?110000000112111010?12003020121022?2?2?111100000?0001000000030?0000000001100000??00?000110011100001001?01000000???0000?030000?0000000?00110??00?0??000?00??000???00?0000?0000[0 1]000?0????????00000?0000?000000010000000?0011000?000??000?0000100000??00000?000000000000?0?00000000?00?0?[0 1]1000000?00???00000100001020?00001011101?0101100?10000000110?00000?01?01?0000?0?0100?10100?000???0000?01??00?0????00???10??000??????????000000?0?0????????000??0011000?????0??????????000000011??????000002000000000??00???0???0???0?0100???????000?0000????????0?0011101?10000?000?100000??0?01?01??????0????00?0???00?00?00?100000?000?1?1000?0?0010010??000000?0?010020?1000?000000??0?000000?0000?000?????0?0000100001001???0111????00?100010?00011101120011?001101011201010?

*Confuciusornis_sanctus* 10010??????????????1?000?00??0001???0??2??0??0??00??01???????000010000?10?0001?1?????????0??????????1021?0?2???0?2????4??111?11010??13111000?11110000001121?1?1??12000?2?111023?2??12??10??11??120211010030?0000000001102000??00?0101??11?100011001??????0?????000?123001[01]00120?????????10??00??011200??01[12]0111110000?01100001001000120000?00?0000?0??0??010000000?000211??0010000000010102101011[01]?10?0000001010000000001010100100011000011000?0000000[01]000???2000001101110?00??1100?10?0000?110?00000??1?00?0000?0?01?0?10100?00??????????1??00?0????0000000??000??01?02???00100000?0????????000??00???0????????????????????????????????????00010???0?0??00??10????????????0???????0001?000???0????0?00111?0?1?000?100??????0??0??1?????????0????00?0???00?00?0??100000??0??1??0?????0?????0??00???????010???????0?????0?0000?0?0??????0??0??????0?0000100?0?0?????01???????????1????00?11101120????00?????-1201010?

*Jeholornis_prima*

10????0????????1???0???0?????001??????????????????0???????????0000?1???00?00?1?1?????????0????????0????1?0?11???02111031??01?0???011031110001??1000001011?1??0??01?0???2???1?221?10???????01????0101101003000000000??0??1000??00?010????1???000?1?????0???0??0??0??12300?10010??????????????????0????00?00000??00?000?00100000?0??001???0000?00000?0?1000120001000?0010000?000?0000?00001000010110000?000000000000?00000?010000000001000001000?00000001100????????011?1110???1?1100?10?0000?110?00000??????????0?0???????0??????????????????????0??????????0??00?????????????????????????????000??00????????????????????????????????????????02?00???0??????????????????????????????0?0??0000???????0?0011101?1?0???000???0000??0?01?????????0????00?0???00?0??0??100000?00??1?10???0?00???????00???????010???????0?????0???0??0?000?00???000?????0?0000100???0?????0??????????1?1?1??0??11101120?11??0?101?0?200010?

*Jixiangornis_orientalis* 100?????????????????0010?????001????0?0??00000??????0100??????000001????0?0??1??0????????00?0???1?00????1??20???02??1031??11?1?000110311100011010000011112101010212000020101?221?10?2?11?001??1?01[12]1101003000?00?000?0??1000??00?0100??111?001?1?????????00?000000?12300?00010??????????????????????0?????000??00?000??11?0?10?11?0?????0000000000?0010011210010?0?0000000?000???0??0????0??010?00100??0000?001000000?00??0000??00000100000?00?0000?001100????????00??11????01?1100?10?0000?11??00000??????????0?0???????0??????????????????????0??????????0??0???????????????????????????????????0?????????????????????????????????????????00010???0??????????????????????0???????????????????????????????0?1?00??00???????????????????????0?????0?0???00?0??0??100?00??0?????0??????????????00?????????0?????????????????0??0?0????????????????????00100???0?????0????????????1???????????????????????????????????

*Yanornis_martini* ?00????????????????1?010?????0?11???0??2??0??????00???????????0000011???0????000?1???00??0????1???0????????4?1???????04??????110?011031110?011?130010????????????????????????23?010?2??10??1??0?0121[23]0000300000??0001???00?0???????0???21?1?0??????????????????0?????300100012??????????10???????1?????????????????0??011?00???0210?[12]??10100001010?1?11?112100111??0101?0101?10000?0?????021[01]101311[01]1100[01]100?0?1?0??0?00??????0??00?1110[01]?1?00??1?0??01100????????01??1????????1100?11??001?11??????0??????????0?0???????0?????0????????????????0??????????0??0?????????????????????????????????????????????????????????????????????????????0???0???0??????????????????????0???????0?0??0?0??????????00111?0?1?00??100???0000??0?01?????????0?????0?0???00?0??0??100000??00?1??0??????????????00?????????0?????000?????0????????000?00???????????????00100?????????0??1?????????0?1??1???11?1101????00?????0?2?000??

*Apsaravis_ukhaana* ???????????????????????0??????????????????????????????????????0002?1?0??0????????????????1??11??10200??1???5?100021??24??????1?010110311000011113003??000?1???1?201000?2?021?23?03?12?010?????1??12130100?00000??20???1?20?0?????0?00??21????????????????????????????300?10?????????????????????0?1[01]?????1?0????????20010?[01]001??21??1????????00000?0?1000121011111?01011010[12]0111110000101021110?[23]120100?11??20?1[01]0010?11?110?1012112111111?0000?1?0??01101????????011??????0?1????????0001?????????????????????????????????????????????????????????????????????????????????????????????????????????????????????????????????????????????????????????????????????????????????0?????????01???0?????0??0?0011100010001?1?0?1?0000????01?010??????????0000??000????????????0??0??1????????00?00100?????0??????0???????0????0000000????00000????000????0?000?10????????0?0?11????0????????????10?0?11?0?1??0?-100?????????

*Yixianornis_grabaui* 100?????2?021??1?????0?01????0?1?1?0????????????????0100??????000?010???0?00?00120???10?01???1???0000??1?0?40??0???0024???11?1100011031110?0010130000100021?101?212003020001023?23?0211?0??1??????2130100100000??000?0??1000?20??0?000001???????????0??0??0???0??0?1230010001???????01??1????????11[01]?????1?0?????10?2?011000?11021??[12]??10100001010?11110112100111100?0110???01?000???0?01021[01]10?31201100[01]100?00100?00?002?101??????11110[01]?1?00??1?0?001100??????00011?11?01????0100?10??000??1?????????????????????????????????????????????????????????????0?????????????????????????????????0??????????????????????????????????????????????????????0??????????????????????0?????????????00?0????????00111?0???000?100?1??000????01?????????0?????0?0???00????0??100?00??0??1????????0???0????00?????0???0???????0?????0???0????000?00????00??????0??00100?????????0?11????0??1??????0???1101101?????0???????2???10?

*Sapeornis_chaoyangensis* ?001???????????????0?010??11000????00??21?0?00????0??????0????0000010??10?00000020??????00????1???000????0?211??0210004??100?????21101111010011100000101121??00?212000?201[0 1]00221210????1??01??1001210?10030000000000????2000??0??0100??0111?000???1???00??0???0000??23000000000??????????????????????????0?00???00000?0010000100????????0000020000?00000?010001000?0?01010?0010000?0???010?1[01]10?11100??0[01]00?0000?0000?00?000??00?00??000001?00??0?00001000????????001?1?1011?1?110001000000??1?000000??????????0?0???????010??00?????????01??00?0?????????00??0??????????0?????000??0????????000??00????????????????????????????????????????02000???0?????0???0???????0?0100???????000??000?0??????0?00111?1?1?000?000???0000??0?01?0???????0?????0?0???00?0??0??100000?000?1?10?0?0?0??0?1???000000?0???0????1??0???0?0??????0?000?000??000?????000000100?0?0?1???00?1?????????1?1??0??111011?0?1???0??01???201110?

*Neuquenornis_volans* ???????12????????20?????????????????????????00?????00??????????????????????????????????????????????00??????????????????????1?11011?1031110?01111000???????????????????????????????????010???????????[01]010030??0????0??????0?00???????????1???????????????????0???????0200??????????????????????????????????????????????110???????21?????10110??0100?001?001?0??[12]??1???01?0???0?????[01]?????????2???20?1??01????????????????10?1????????1?0???1011???????0?000???200000110??????????????????0??0??????????????????????????????????????????????????????????????????????????????????????????????????00???0???????????????????????000??0100??????????????????????????????????????????????????????????????????0111?????0??????????????????????????????????0?0???00???????????00??0??1???0??????????????????????????2???????????????0?????????????????????????0010?0????????0????????????????????????????????????????????????

*Patagopteryx_deferrariisi* ?0????????????????????????????????????????0010????????????????????????????????????????????????????2????????????????????????????????????????0?1???????????211101?????????????????????2?010001??100121301001?000???????1????????00?0?????1??????????????????????0?????0??????????????00?1??????00??111110000?????????010000000????????0????????000????0101?1210[0 1]00???[0 1]?10?0???01000001[0 1]0001?[1 2]00???30???????00?2000000?010120????0?000[1 2]11001100000?[0 1]?0??0?100?1?200??01?0???0?001????????00???0?1????????????????????????????????????????????????????????????????????????????????????????0??????0?0??0?110????????????????????0??????????????????????????0020000??00??????????0?0?00??0001?00000????????00111?00100???????1?0000???????0100??0?0???0?000???00????0??????00??0??1??????0?10???????????00???010???????00000000??0?????000000???00?1???00000010??????1???0????0???0?1???10????10?0110?0????0??00??????????

*Cathayornis_*spp. ?0?????????????????1?010?????0?1??????????0???????????????????00?????????????00001???????0????????[02]????????3?????2????4???0??11?02?1031110?011?1300?0?01111?0?00?12000?201?1022?23?????????1???0??[12]1[01]1?0?3000?0???00????0??1??0??0100???111?????????0??0???????0????2300[01]0?012???????????????????????????0??????0????0111??0010?20??1??10110?101????010?01200010010110[01]?01020111110?[01]?1?????210120?11?01?0101011?0?000001???1?0?0?1[01]1?0???????????0??01110????????01????????01??????????0???110?00000??????????0?0?0100?10100?000???????????????0??????????0??00?????????????????????????????000??00?0?0????????????????????????????????????0??00??00?????????????????0?0100???????0?????????????????00111?0???0?????????0000???????????????0?????0?0???00?0??0??????00??00?1?1??0???????????????????????0????100??????0???0?????00?00???????????????00100?????????0???????????000???1???0111121?????0?100?0????????

*Concornis_lacustris* ????????????????????????????????????????????????????????????????????????????????????????????????????00????????????????????0??11?02?1031110?0?1???0030????????????120?102??11?23??3?0???1???1???0?1[02]1[01]??0?3000?0???0????????????????001??1???????????????????????????2300??????????????????????????????????????????????111????10?20??1?0101100101?0?0010001?0??[01]001?110??0102?1???10???1[01]1?[12]??????????????[01]????????????00?????0?100??1?00??1?[01]1??0??????110?????????????????????1100110??01????????????????????????????????????????????????????????????????????????????????????????????????????????????????????????????????????????????????????????????????????????????????????????????????0??????????00111?0?1?0????????????????????????????0?????0?0???00???????100?00??0??1????????????????????????????0?????????????0????????0?????????????????0??0010??????????0?????????????????????01011?1????????????????????

*Gobipteryx_minuta* ?0?????????????????1?01010?0?0?????????????????????????????11?000?0??????????1?1?????????????????????????????????????????????????2????????????????????????????????????????????????????????????????11???????????????????????????????????1????0??????????????????0????????1100100?00110?10?????00?????0???0??0???1?????????????10??????????11??10000?0?100012000[12]001?1?0110???0????????????1?121??[012]0?????1?0??1????????000?0???10?001111000?1?11?0????????????????????????????????????????????????00000??????????0?1?01????0???0?0????????????????????????????????????????????????????????????????????????0????????????????0??????????????????0?01000?000??00???000???????????????????????????????????????????????????????????????????????????????????????????0????????????????????????????????????????????????0???????????????????????????????????????????0???0??????????????????0??????????????????????????-????????

*Vorona_berivotrensis* ???????????????????????????????????????????????????????????????????????????????????????????????????????????????????????????????????????????????????????????????????????????????????12?010011101001112110???00????????????????????0?????2?????????????????????????????????????????????????????????????????????????????????????????????????????????????????????????????????????????????????????????????????????????????????????1010111100001?000000?????????????????????????????????????00?????????????????????????????????????????????????????????????????????????????????????????????????????????????????????????????????????????????????????????????????????????????????????????????????????????????????????????????????????????????1???????00000000?00??????????????????????????????????????????0?10001????????????????????????????????????110?00000010??????????0????0?????1???1?????????11??????????????????????

*Songlingornis_linghensis* ?????????????????????0?0??????????????????????????????????????00?????????????000?0????0??????????????????????????????????????11??[01]???3?????????????????????????????????????????????????????????????????0????0?????0?????00?????????????????????????????0???????0?????????0?01???????????????????????????????????0???????????????21??[12]?000100?010???1?11011????????????????????????????????????????????????????????????????????????????????????????0??0?????????????1??1?????????????????????????????0???????????????????????????????????????????????????????????????????????????????????????????????????????????????????????????????????????0??00??00?????????????????0?0?0????????????????????????????1?1?????0????????????????????????????????????????????????????????????1??????????????????????????????????00????????????????????????????????????0?1????????????????????????0???????????????????????????????????

*Pengornis_houi* ?0010??????????????10010??0?00??????0?12??0??????0?????0??????000?001????????0?0?00??00???01??????20???0???2??????????4????1?????2?1031111?0??????03??001?1??????????????????2??2??????????1??????211???0?000??0??000???0??0??00???0???11?1?000??????????00????00001230000?000?????????????????????????????0?????0???0011??00100????????01[0 1]00?00??????0??1??012111[0 1]0?011010001[01][01]1?????1??1???10[12][12]0??1??1?11??????0????00?0????0??0[01]?1?00??1?11??0?0??0?100????????01?????00?01?1001011???1??110?00000????0??0??0?0?0100??0?????00???????????????0??????????0??000??????2?????????????????????0?0??0?????????????????????????????????????????0?0?0???0?0??00???0???????0?0100??0?0??0??1?0????????????00111?0?1000????0???0000???????????????0?????0?0???00?0??0??????00??00?1??0?0???0????1??????????????0????1?00?????????0??????0?0?????00?1???????00100??????????1?1????????000???100??1?1121????0???????????????

*Hesperornis_regalis* ?0?????????????????00010100?00??????00021?001????00???00????1?000?001???1?0001?021???00??10101110?2100??11?50100?210?24??11??10??00103111??????????3??0202100?1??01000???021023?03?12???0001??11?1213110010??0?00??01?1?0000?000???00??2??1?1111????0???????10000??10?1?10?1121200110?1010??1110011100??00?0???1010121011000012?0?000100100?0???10?0???00011??0?????????0???0????0??????????????????????????20?1100001112110211110021111122122002?0?????00??????0001?0???0?000?0100?11???1??01??00000??????????0?1?0100??0?????00????????0???0??0??????????0??00????????????????00??0????????0?0??0????0????????????????????????????????????0?000???0????????????????????000???????0?0??000???????0???01?????????????201??000????01?????????0????00?0???0??00?0??????00?????1??0?0?0?0???0???????????????0?????000?????????0?????0000?????00?????????001?0??????0???1??????????000???200???011????0?00??0000????????

*Baptornis_advenus* ??????????????????????????????????????????????????????????????????????????????????????????????????2????????5?????????24???????????????????????????????????????????????????????????????????????????212??????????????????????????????????2????????????????????????????????[12]????????????????????11001??00??1??????????121011000012????012?0?????01?10?0???000????0?????????0???0??????????01?[01]1[01]???????????????20?11000011121102111100211111?21020?2??????????????????????????????????????????????????????????????????????????????????????????????????????????????????????????????????????????????????????????????????????????????????????????????????????????????????????????0???????0?0??000???????0???01???????0?????201??000????01?????????0????0000???0????????????00?????1??????0?0???0???????????????0???????0?????????0?????0000?????00?????????0210???????0????????????????????2?01??011??0????0??0???????????

*Ichthyornis_dispar* ?????00????????1220?????????????????????000111????0???????????000??1?????1000??0210??10???1?01111?2100?1???5?000?21??34???01?100?001031110?011113003??0102?1??10212000????21023??3?12??10011??11?1213110???00?0??20?????00????0??00????21???????????0?????????????012300[12]0?11???????????10[01]1?111011101001020???101?1200111?101[12]?211022010100001010?1111011211011110010110111010000101[01]10112111123120110011?12001[01]0010?112110211110021111[1 2]2?100002?0??011?100?20???0110??1??00?00100?11??01??01???????????????????????????0?00?00??????????????????????????????????????????????????????1?0?????00???????????????????????????0????0100?????0?00?0000?00?????????000?0???????00??000??0?01?0000??????0??00111?00100?0?10201??000????01?0???????0????0000?0?0???0?0??1???00??0??1??????000??00100?0???001??01??2?0?00000?00000000????0000?????00?111?0?00001????10?1?0?0?11?0??0????0??0?200111011?1????00???0?0????????

*Iaceornis_marshii* ?????????????????????????????????????????????????????????????????????????????????????????????????????????????????????????????1101?010311??????113?03????????????????????????????????2??1??1???????21?????????????20???1??0??????????????????????????????????????????????????????????????????????????????????????????????????????21?02[23]11???1101010?11110112101???????????????????????????????11131401110111020010??1?1?12110212110021??????????????????????????????????????????0100?11???1???????????????????????????????????????????????????????????????????????????????????????????????????????????????????????????????????????????????????????????????????????????????????????????????????????????00111???????0?10???????????????0??0??0??????0???????????????1??????????1???????????00????0???00???01??????????????0??????1??????????????1???0?00????????????????11?0??0??????????????1?11?1????????????????????

*Limenavis_patagonica* ???????????????????????????????????????????????????????????????????????????????????????????????????????????????????????????????????????????011?13????????????????????????????????????????????????????????????????20??????????????????????????????????????????????????????????????????????????????????????????????????????????????????????????????????????????????????????????10000[01]010101011?11[12]313011?011????????????????????????????????????????????????????????????????????????????????????????????????????????????????????????????????????????????????????????????????????????????????????????????????????????????????????????????????????????????????????????????????????????????????????????????????????0?0????????????????????????????????????????????????????????????????????????????????????????????????????????0000???????????????????????????????????????????????????????????????????????????????????????

*Lithornis_*spp. 100????????????????01010100?00?111??2?02?00?0???????????????10000?00????02?101?1?????????1????????2????????[67]?1????????????[12]??11000010311110011?13003??000210101?20200102??2102?????12?01000???11?12130100?????0??200?01?2010???0?0?0???21???12???????????00???00000??300?11212100?00011111010110?11[01]1101?1111?01?10121012?0111[12]1211122110100?01011011110112101111100110001[12]1110000101010101111113130110[01]11102[01]0120110?11211020110002111122210010[01]?000011?1????????011?111??00???????????01???1??????????????????????????????????????????????????????????????????????????????????????????????????????????0????????????????0?????????????????????????????????????????????????0???????0?0?????????????????????????????????1??000?????????????????????????????????0??????????????????????????????????????????????????0???????????????0000???????????????????????????????????????????????????????????????????????????????

*Hongshanornis_longicresta* 101????????????????11010?????0?11???0????00???????????????????000?001??00????1?1?10???0??0??01??1?0????????[234]??????????4???1??11?011103111000111130020?0112???01?20????????2??23?23??2??100?1??????21[23]01000000000?000?01?1????????0?0????1?1?0????????????0????0000?1?300???012???????????????????????????00??????1?????0?????110????????01?0??11???0?10???21000111???110???2????0????????????1??2000??000100?0??0?0??000????????1???1100??[01]?00??2?0???1????????????????????????0100?11??001?11??????0??????????0?????????0??????0???????????????0??????????0??0???????????????????????????????????0?????????????????????????????????????????0?0?0???0?????????0????????????0???????0??????????????????01?1?0?000?0?10???????????????????????0?????0?0???00????0??100?00??0?????0??????????1???00?????????0?????????????????0??0?0?????????0??????????00100?????????0????????????1????????????????????????????21??1??

*Liaoningornis_longidigitris* ?????????????????????????????????????????????????????????????????????????????????????????????????????????????????????????????????????3??1??0???????????????????????????????????????????100?1????0?21211003000?????0??????????????????????????????????????0?????????????????????????????????????????????????????????????????????011?01??????????0???0?????????????????????????????0?????01???2??????????????????????????01?1?0??001111110201000?00??????????????????????????????????????????????????????????????????????????????????????????????????????????????????????????????????????????????????????????????????????????????????????????????????????????????????????????????????????????????????????????????0????????????????????????????0?????0?0???00???????????00??0???????????????????????????????0???????????????????????????????????????????0010??????????0??????????????1?????????????????????????????????

*Crypturellus_undulatus* 10????012?020002?2001010100?00?111?12002100001011100010???1?10000?000??102?001?1?????????1110111122100?01107?110??12?24??121?11010010311011011113003??001210001?202201120021023?23?12?01000100110121301?0???01000200001?20000200?0?001021???120??01??????0????00000103002112121[01]010001111101011001101111111110010101210101121121211112?10100021011010111112100111110110001201100001110101011111031401011111020?120110?11211020211002111122210010110??011?10102000001111110000??1100?10??01??11????????????????????????????????????????????????????????????????????????????????????????0?????????????????????????????????????????????????????0????????????????????????????????????????????????????????????????????????2??????????????????????0???????????????????????????????????????????????????????????????????????????????????????????0?????????????0?????????????????????????????????????????????????????121001??

*Gallus_gallus* 100???102?021002?2000010100?00?111?120021?0001?1000001????1?100100000000021101?1?????????101[01]1111221?0?01007?120??12?24??1[12]1?11012010311020011113003??001211111?2?2001120021023?23?12?0100011011??213010010001000200100?20100200?0?0???21???121??01???0??00???000001030011021212111112211011111111111101100011010111210101120111211112110100021011111111112100111110110001201100001011101011111131401001211021?121111?112110202110021111322200111?00?0110001020?000111111?000??0010110??01??11????????????????????????????????????????????????????????????????????????????????????????0?????????????????????????????????????????????????????0????????????????????????????????????????????????????????????????????????2??????????????????????0???????????????????????????????????????????????????????????????????????????????????????????0?????????????0?????????????????????????????????????????????????????121001??

*Crax_pauxi* 1001011?2?021000??001010100?00?111?120021000011100000100??1?100000000000021101?1?????????1000111122100?111?7?120??12?24??121?11002010311121011113003??0002100?1?2?2001120021023?03?12?0100011011?12130100100010002001?1?20100200?0?0???21???121??01??????00???000001030021021212111112211011111111111101100011010111210101120111211112110100021011111111112100111110110001201100001011101011111131401011211021?111111?1121102021100211113222001101000011000?020?0001111110000?11100011??01??11????????????????????????????????????????????????????????????????????????????????????????0?????????????????????????????????????????????????????0????????????????????????????????????????????????????????????????????????2??????????????????????0???????????????????????????????????????????????????????????????????????????????????????????0?????????????0?????????????????????????????????????????????????????121001??

*Anas_platyrhynchos* 100???102?021002?2101001100?00?111?10002??00?1?100010?0???1?10200000100?021101?1?????????101?1111221?0?011?7?11???10?2410121?11011110311111011113003??0012000?1?2?2001120021023?13?12?0100011011?121311?0???010002101?1?20100?00?0?0?1021???121??????????00???000001130021021211111112211021112111111101100011110111210101020111211113120100101010?1[01]111112100011100110001201100001011101111111231401010111021?111110?111110102110021111[23]2210010210?0011?10?020?000111111?000??0100?11??01??11????????????????????????????????????????????????????????????????????????????????????????0?????????????????????????????????????????????????????0????????????????????????????????????????????????????????????????????????2??????????????????????0???????????????????????????????????????????????????????????????????????????????????????????0?????????????0?????????????????????????????????????????????????????121001??

*Chauna_torquata* 000??1012?0210002?101010100?00?111?100021?00010100010100??1?100000000??0021101?1?????????1011101112100?111?7?11???1012412111?10000010311111011113103??0012101?1?2?200102?021023?23?12?0100011011??213010010000000200101?20100000?0?0?1021???121??01??????00???00000123001102121[12]111102211011112111111101100011110111210121020111211114010100101011111101112110211100110001201100001011101011111231401011111021?111111?1121102021200211112222001001000011000102000101111110000?11101?11??01??11????????????????????????????????????????????????????????????????????????????????????????0?????????????????????????????????????????????????????0????????????????????????????????????????????????????????????????????????2??????????????????????0?????????????????????????????????????????????????????????????????????????????????????????????????????????0?????????????????????????????????????????????????????121001??

*Pedopenna_daohugouensis* ????????????????????????????????????????????????????????????????????????????????????????????????????????????????????????????????????????????????????????????????????????????????????????????????0?0?0??0010??????????????????????????????????????????????????????????????????????????????????????????????????????????????????????????????????????????????????????????????????????????????????????????????????????????????????????????00?000?????0??0????00????????0???11???????????????0??????????????????????????????????????????????????????????????????????????????????????????????????????????????????????????????????????????????????????????????????????????????????????????????????????????????????????????????????????????????????????00????0???00???????????00??0???????????????????????????????0???????????????????????????????????????????0?10??????????????????????????????????????????????????????11???

*Epidendrosaurus_ningchengensis* ??????????????????????????????????????????0??1????????????????10??????????01?????0???????0??????1??????????????????????????????????001??1??0??????001????????????????0???2???03????????????0????????0000030?0?0???0?????1???????????????1????????????????????????????3?0??????????????????????????????????????????0???????0???????????????????0??0?0??????1???0?????????0???0???0????????????????0??????000??????????????????????????100?00000??0?0??0?010????????00??????1???????????0??00??????????????????????????????????????????????????????????????????????????????????????????????????0????0??????????????????????????????????????????????????????????????????????????????????????????????????0011??????0????????????????????????????0?????0?0???00?????????0?00??0???????????????????????????????0?????????????0????????0????????????????????0010??????????0????????????????????????????????????????????????

*Epidexipteryx_hui* ?01???????????????????1??????00?????0??2??0011?????????????????100?0?0????00001020???10000???????????????0?[01]????1?1?123??10??0????0101?010?0??????00?1???????????022?0???211?03?0??????????0????011?0??0?10?000???00??1?1?0???00?0?00???0?0?????????1??2?????0???????2?0?????0??????????0???????????????????????000?0????00??0?0??????????????0??0?0??????1???0?????????????00?[01]0????????????????????????????????????????????????????10?0?0?0????10?00???0?????????1??11??1????????????0?00?100?????0??????????0?????????0?????0?????????????????????????????????????????????????????????????0????0?????????????????????????????????????????00200??02?????????0???????0?1????????????????????????????0011??1???0?????00??????????01?????????0?????0?0?????????0????0?0????0????????0???????????????????0???????00??????0???0????0?????????0??????????001?00?????????????????????0????0?????011??????0????????000001?

*Incisivosaurus_gauthieri* ?00?00?01?001??1??10001110100[12]001?010?120000110001010110101111210100001001?1?000210??00?0??????????????????????????????????????????????????????????????????????????????????????????????????????????????????????00??00??01?0??0?0?????????100000001000102?00010?100??????0000000000100001??0000???0000101?1100??0000???????????????????????????????????????????????????????????????????????????????????????????????????????????????????????????????????????01020?00???0??000?0?0????????????11?1100000????1000000?0?0000?000?0?000???1000??1??00?0????000?110??010??01?0000?00000???00?00????0100??0111000????????????????001000000010000????00200??02?1?000000000?????00110???????????????????????????????????????????????????????????????????????????????100?????????????0??0?001????????????????????????0201100????????????????????????????????????????0?0?0??????0??????????000?0??00???????????00?????00????????

*Citipati_osmolskae* ?001001001001??221000101111?02011?000102100011000010001000110121120?0100010111?1?????????1011101100101211001??201??002200111?0111200110100100?11000001000211001??02012020??10111111?2101000000000?000000000000000000011020000000?0?001000?000002011???0???[01]000020001220001000000001102?1??0000??0000010001010??000002?011000[01]0?00?0010000101020??????0????111000?0?0010000?000000000000?00100?0??0?0???0000000????00000?2000000000000000000002?000?000000000020?000110??00[01]10?011011000000011?1100001????1000001?0?0001?11000?001???1002??0???0?0????000?000??000??01?0000?0000000000100?????100??0111000????????????????101000000010000????10201???2?1?0000000???0????????????????????????????????????????10???00?00???????????????????????????0?0?0???00100????000?00??0???0?00?????????0?0?0?00?0???0?00211??????????0??00?000?0???????0??????0?0000100?0100????10?????????1?00?0?00??00???00?0??0?????0-????????

*Oviraptor_philoceratops* ?00?0??????01?1????0??1??111?2011???0???1?0?11???01?0???0?11?121120?01?00?01?1?1???????????????????????????????0????????????????12??1?????00???10??00?00??1??0????????????????????????????0????0????0??0?????00?00000???2?00?0????????????10000?0?0???????0??0???0????00???0?00????????10?0?00??0??00?00?1?1????00????0?100????0????????01010?0?00?0?0????1?100??????1100???0????????00?????????000?0??00000??????????????????????????????????????0??000?????20????1?0???02?0??1101100???0011?1?????1????10????1???0?01????????01???????????????0?????0???00??0?0??0??00?????00???????????????????0?????????????????????????????????????????1020????2?1?0000000????????????????????????????????????????1???1???0??????????????????????????????????????????100????000???????????0????????????????0??0???????211?????????00??????0??0??????????????????????00??0??????0???????????00???????0?????0?0?00??????-????????

*Microvenator_celer* ??????????????????????????????????????????????????????????????21?20?0?????????????????????011?0?11000121100???0?1?1002????????????00?000?11000?????0010002???0000?????????????1110??0101100?0000010???????0??0???00??11?20?0??00???0?1??0?????????????????????0???0?2200000????????????????????????????????????????0??001????????????????????20000?000?01?????00?0??010?00?000000000000010000?????00????????0???0??000002000000000000????????????10??0??????????????1????????0???????????00????????????????????????????????????????????????????????????????????????????????????????????????????????????????????????????????????????????????????????????????????????????????0???00??0001?01001?????????????010000?0?????100???????01?01??0000????00?????????????0?????0????????1????000?10?000???0010?0001????1??????00??00?10??00000000??000?0??00000??????????1?01??11100??001?????????1??01?????0??00100??????????

*Caudipteryx_zoui* 00010??????????????0?111??10?0001?10???21000???0?0????????????21120?0????????0?1??0?????00????0??00????1???0???01?????30?????0???0??0???01?0???100000?000?1??????0201202?1?1????101?11?????0????01000??1?00?0?000000?1102000??00?0?001000100000?00????0???00?00000?1[0 1]2000000000???????????0??????0000????1000???0000??0?0000?0?0?????????????20??0?0?000?11??00???????0?0???00?00????????????1??0000???0000000?000000000??0?0?00????0000000??0??2000000??0????????01101100??01?110???0??000?10???0000?????0????0?0?0001?00?????0????????????????0????00???00??0?0??0??0?????????000?0????????1????0?????????????????????????????????????????00201???2?????????0?????????0??0???????????????????????????1???????00??000???0000???????????????0?00??0?0???001?0?0???00?0???0????10?????0???000??0?00?0???0?0???11??0????00????0?000?0000???0?0?????0?0?00100???0????????????????1?10???01-100011000-010011000-?10001??

*Heyuannia_yanshini* ?00?0????????????????1?1?????2????????????????????????????????21120?01000?0111?1???????????????????????????1???01????2?00???1011120011??00000??100000100021??????02012020011011?11101101?0000?00011?000000000??0000??11?2000?000?0?001000???????????????????0002?0??2200?1000000??1?????0?0??0??0000?00??1?10???000?????1??0[01]0??02??????0101??0??0???0?0?0101000?????1100???00?0?????????????????00????0?00?00?000?0?000??0?0?000?0?0?0??00?0????00?000000????????0110??00?1?0?1101000??000?1????????????????????????????????????????????????????????????????????????????????????????????????????????????????????????????????????????????????????????????????????????????????????????????????????????0011000?001?0?01??101000??0101??11??0??????00000???00?????1?000000010??1?1?0??????10???0???0??0?000????11???0???010000000100000000??000100?000000010??????????0??1?0??00?1???1??00??0101000?00??00100?-????????

*Rinchenia_mongoliensis* ?00?0????0??????????0111?1???2?11?00010??00???0000?????00??1?12112??01000??111?1????????????????1??????????????0?????220?????????2??1???00?0???1??0001000?1?????1???????????????1?????????0????0??0?0???????0??00??001??2?00??????????????0??????00????????00??210??????2??0120????????10?0?10??0???0????????????0??????????????????????????????????????????????????????????????????????????????????????????00???0?00???????????????????????????????????????????????????002?0????????????00?1?1?00001????1??0001?0?0001?11??0??01???1???????????0????000?000??000??01?00???0?000000?0????????100??0????0????????????????????????????????0??????????????????????????????????????????????????????????????????????????????1?1001??????????????????????????????0???????????????????0????????????????0???????????11????????????????????0??????????????????????0??????????0????????????0???00???????????0??0??00?-????????

*Conchoraptor_gracilis* ?0010??????????1???00111?1???2?11?000??21000110?00????1?0????121120?010?0?0111?1??????????01010110010??1?012?110??10????01?1?????20011000010???????0010002100010102002020??101101[01]1?11010000??00010000010?000??000?001102?0?0000?0?0010001000002001???????0?0?0200012??????000000000????????10???0100100????????000????010??????????????????????????????????????????????????????????????????????????????????00?00??000002000000000000?0000?002?01?????????0?020?000??0??002?00???????????0011?1?00001????1??00?1?0?0?01?11??0??01????0?2????????0????0?0??00??0?0??01?00???0?00?000?0?0??????100??0???00???????????????????10???0???????0????????????????????????????????????????????????????????????????????????0?0??????????????????????????????0?0????010?????010?????0?????0???????10???????00?0???????21?????????????????0?000000???????????????0?1?00????????00????????????0?0?00???1??????0?00??????-????????

*Chirostenotes_pergracilis* ?????1??01?01101??0???1?110?0??????????????????????01010??????21120?00000201???1????????????????1101?12????1?12???0????????????????101?1??????????00?100021?00101022120201110?111[01]??01??100???00?100000200000??000?0?1??20?0?000?0?00100????????????????????000?????[12]????10????????001????00????????????01?10???0?????011??00????????????????20000?00000?????????????????????????????????????????????????00000?00??00000?00?0????????000000?0???0000?????001020?00???0????????1????????0?001???????????????????1?0?00???0??????????????????????????????????????????????????????????????????????????????????????????????????1000001000???????00200???2?1?000??0000??????????0???????0?0??00???1000?0??????????????0??0??101000?????1?0100?0??0?00??00000?001?0??1?1?000?01?????1?0??????10?????000??0??????02?1???0110?1???????000000000???00????00??00010?0??0?????1?0??0???????0?1???????1????1???????????-????????

*Avimimus_portentosus* ?00?0???10011?00??0??1?1???????1???1?????00?11??00100110??????2?1???00?0??0111????????????011010110101?1?00??100????????????????????????0100??????????00?211?01??0201202010?011?0?1000100?0???00011110020???00??0?0?01?02?000000?0?001000????????0?????????00?01??0?02001????????????????????0??0???00??0??????????02?01000??????????????????????????????0?????0?0?0010000?000000000????????????2???????????[02]0?000?0000020?010000000000000??00?01??0??????0????????????????????????????????1?0???????????????????????????????????????????????????????????????????????????????????????????????100??0?1100????????????????????0000001?0?00???????????????????????????????????0???????0001000001??????????????00001???????101001??0101?0100?0?000??001000??00???????0??0?1001??101????0000100000?????1001101102?????101010000000????00?000???001000000000010?0????1??01????0??00?1???1?????1??0110????????0???-????????

*Falcarius_utahensis* ??01?11100001111010?????1????00??????????00??????0000000??????100?000??0???????001010001?1??0001110101210100012000100??0000??????000001000100010?0000100?011001010220301010100100010000000000000110000000000000??000?10?00?0?000?0?001000????????0?001???00?0?0?0??11?0??000???????000??00???0??00?00100?0?00??0000???010000[01]0??????????0100020000?00000001110?1?0?0010000?001000000000010000???0100???0000000?0000000002000000000000?00000000?0000??00000?0?200000??0???00000?1100?00000000???????????????????000?00?00100????0????????????????????????????????0?????0??????????????01?00????00?00????????????????????????0000001010010????0??00000001???0??????????????0?0???????0001000001000??1000??1010000000100000000000?0101?010000000001?0000001000?0??00101000010?1?01?11?1000100100000000011101002?0?101111000000101000100000??000001000?000000?0?10?10000?011011002?00?10????????????????????????????????

*Beipiaosaurus_inexpectus* 0??1???????????????????????????????????????????????????????????10?10?????????????1001001?100????1??????????????000??0220?????????0000??0????0?1000000?0000??001???20220??0??0????0??00?0??00??????0000????0???0??00111???0?0??0????0010?0??????????0??????????0???0??????0?????????????????????????????????????00?????01000?????????????00?00????????????????????????????????10000???????????1??0100???000000???0??0??0?????0???????0000000000???0?0?00000????????????10?????1?1100??0??000?1?0?????0????????????????????0?????????????????????????????????????????????????????????????????????????????????????????????????????????????????????0??0?00?????????????????????0???????0??????????????????????1???0?00??00???00?1????????10?????0?0?????????00???????1?1?0????????1??1?????10???000?0?00???????????????????0??0?01000?01010???0?11??10??0?1?0???1???????????????????1???????????????????????????000??00?

*Segnosaurus_galbinensis* ??????????????????????????????????????????????????????????????21??10?0000?000???0100?001????????1??????????1?0?0??????????????????0?10?1?00000????0???20011100102020221??0110201101?11001?00?00?11000000021?0??00?01?1??0?0???00?0?010?00??????????0?????????00???????0?????????????????????????????????0?0????0??????????????????????????????0??????0????1????1???0010000?00100000?????????????????????????00?00000??10??????00?????000000000??21?0????00???????????????????2??????????0????????????????????????????????????????????????????????????????????????????????????????????????????????????????????????????????????????????????????????????????????????????????????????????????????10????????????0?00???????21020010?0101??02???????????0?00??????????????0??01??0??1??1????????2???????0??110????????1???????10100??1?11122210111111112?111201??????????0???1???????????????????000????0??0??00??????????

*Erlikosaurus_andrewsi* ?0012???2?0?1?1??1010011100??0001?1000001000010000000000??11112100100000020001?001001001?????????????????????????????????????????????????0?????????????????????????????????????????????????????????0000?021?0??00??11??00?0?00????????????0?00000010010??00000?000????0?000002000001???10?0100???0000?0?10000??0000????????????????????????????????????????????1?0?0010000?0010000000?????????????????????????????????????????????????00000000????????0000?0????000?????000?0?????????0????01?0001100????1?10000?0?00?0?100000000???0??0?00??00?0????000001000000??00?0200?00000101000?00001?000??00?0?00????????????????00000001101????0???01001000200000000000000??????0?????????????????????????????????0?00???????????????????????????????????????????0000???????00??0???0?011????????2??????????????11210?11???????1?0?1????????????????????????1?01001000????01??????????010?0?000??????0?????0?????10????????

*Alxasaurus_elesitaiensis* ??????????????????????????????????????????????????????????????210?100????????????1001001??????????0?01010000?1?0101002?0?1???????????0?000??0?10?0000120?11??0??1?20?21???11?????????1????0?????????00000?10000??001?1??00?0??00?0?0???00??????????0??????????0?????0????0??????????????????????????????????0??00???????00??[01]0????????????????0????????0??11??01??????0?0???010000???????????10?0100???000000???0??0?????????????????????????????1000000?0????????0?????????????????????000????????????????????????????????????????????????????????????????????????????????????????????????????????????????????????????????????????????????????0??0020?????????????????????0????????????11101?????1??0?11??0?00??010?0?????01?????????2????????????????200??0????1?1??0??0????1??1?100???????0000000??1??0???0?00???11?0????01100??11?1?????1111?????1?00???10?1???0????????????1????????0??0000?????0??????????????

*Neimongosaurus_yangi* ??????????????????????????????????????????????????????????????21????0?????????????0??001?1??000?110101?1?0?10?2?1?1?02200????????0001010000??????????????111001?2?????????????????????0??00????0??000000021?00????0??1??00?0??00?0?????0???????????0???????????????1[12]200?0?????????????????????????????????????0??????0?0000?0??????????00000200?0?00000?0?11001?0?0?10?0???010000?0?00?????????????????????0???????????20?00???????0?00000000?0210??????0????????0??????????2?1100?000????????????????????????????????????????????????????????????????????????????????????????????????????????????????????????????????????????????????????????0????2??????????????????????0???????0??1??11???????0??01?10?0?00???????21?2????????????2?1???1?00??00000?1???0??00????00????00??????100010?2???????00?????1???0?0111?111001011????1?1121?????111111????10????1??11??0?0??0?100???1?1?????????????????????????????????

*Erliansaurus_bellamanus* ???????????????????????????????????????????????????????????????????????????????????????????????????10??1??????????1???????????????0?0???10?0?0???00000????1?0?1??????????????2??1????10?00?0?0001?0??00??????????00?????????????????????0?????????????????????0??????200??????????????????????????????????????????????????0???????????????????????????????????01?0?0?10?0???010000???00010???????100???000000???????????20?00???????0????????????????0??????????????????????????????????000????????????????????????????????????????????????????????????????????????????????????????????????????????????????????????????????????????????????????????????????????????????????0???????????????????????????????0?0?1?0?01????????????????02?0???1?000??????????????00100???????0?????????00???100?????00?11????????????????011?111111??11?1???0?1?11111011?0?????????1??????????0?1?????????????????????????????????????

*Suzhousaurus_megatherioides* ??????????????????????????????????????????????????????????????????????????????????????????????????0101??01000??0??1????0??????????001010000???????????200111001?2020?2?10010020110101100?00??????????????????0???????1????????00???010????????????????????????0?????0?0?????????????????????????????????????????????2?010000[01]????????????????20000?00000?01010?1?0?0010?0???0100000??00?????????????????????00?000?00?1020000?????????????????????0??????????????????????????2??????????????????????????????????????????????????????????????????????????????????????????????????????????????????????????????????????????????????????????????????????????????????????????????????????????10111100100000?11010?00??????021020010?0101?00201010????????????????????0???0??01??00?1????1000?0?2???????00????????????????11?100011????1112212111110??????????????????1?0??01102?0?2??????????????????????????????????????

*Nothronychus_*spp. ?????1112??11?1?0?0????????????????????????????????00000????????????????????????????1????????????10101?10?01?1?00?100220?001?????00000?100?00????00000200??10?1?2020221100100201101?1100100?100011000000021?00???00??????0?0?000?0?010?00?????????????????????0????1[12]200???????????1??????????????????????????????????010000[01]0??????????00000200?0?00000?0211001?0?0?10?0???010000???00010???????000???00???00?000000?1020??000000000?00000000?0210??0?000?0????000????????0???1100?000?000????????????????????????????????????????????????????????????????????????????????????????????????????????????????????????????????000?01100???????????????????????????????????????0???????0?0??1010110???0000?110?00001?0?0?021?20010?1101??02???1?10000?0?000?10?????0?1?0000010?0??1?11?1?0010020000?00001110?1?2??????1?1110?0001???111122210111111?12101120??0????1?1?0???10?????1???10????????????????????????????????

*Enigmosaurus_mongoliensis* ?????????????????????????????????????????????????????????????????????????????????????????????????????????????????????????????????????????????????????????111?01?2020221100??0201101??????????????????????????????????1????????00???010??????????????????????????????????????????????????????????????????????????????????????????????????????????????????????????????????????????????????????????????????????00?000000?10?????????????????????????????????????????????????????2???????????????????????????????????????????????????????????????????????????????????????????????????????????????????????????????????????????????????????????????????????????????????????????????????????????????1??1?????????????????????2?020?10?1101?????????????????????????????????0??01?????1??????????????????????????????????????????????????1?122121100????????????????????????????????????????????????????????????????????????

*Nanshiungosaurus_brevispinus* ?????????????????????????????????????????????????????????????????????????????????????????1???????101?121???0??????????????????????????????????????????????????????????????????????????????????????????????????????????????????????????????????????????????????0?????1???????????????????????????????????????????????2?0??0??????????????????????????????????????????????????????????????????????????????????0????????????????????????????????????????????????????????????????2?????????????????????????????????????????????????????????????????????????????????????????????????????????????????????????????????????????????????????????????????????????????????????????????0??????????1???1??0????????????????????????21?2??10???01????????????????????????????????????01??0??1????????????????????????????????????1??????????????11222??11?????????????????????????????????????????????????????????????????????????

*Therizinosaurus_cheloniformis* ??????????????????????????????????????????????????????????????????????????????????????????????????????????????????????????????????0010?0000????0?00200?????????????????????????????????????0??0?11000000021?0????00???????????????????????????????????????????????????00??????????????????????????????????????????????????0??????????????????2000??00000?01?1001?0?0?10?0???010000?????010???????100???00000??????????????????????????????????????0??0100????????????????????????????????00??????????????????????????????????????????????????????????????????????????????????????????????????????????????????????????????????????????????????????????????????????????????????????????????????????????00?1010?00100101??????????????????????????????????????????001?0????????0??????????10??0000????????????????????????1110111111????????????????2?111?0?????????1???01????0????????????????????????????????????????

*Haplocheirus_sollers* ?0010???00010?0?12011011101111001?000020100?0?1010??0001?0010000010110110?1000001101010100??10??0?0111201??0?00001?0???0?00???????100210001210?0?00010???001?00?101002010010?011000?00000000?0000?0000000??00?1110001100010001?0???0010?01000000000??0000001?00000?102000000000????00???00???0???000000??0??0??0000?0?0?000????0?????????????20000?000000010101??????100???000?00???????????0???00000??0000000?00?000100200000???????000?0??0???0100?000???0?0??????????00?0??????????0?0000001?00001??0?0000000?0?01000000?0??11???0??0?01??01?0????000001000000??00??100?00000000000?000????00??0??0?0??????001????????0?0??0?0000????0???00000??00?00000??00??????????000???????0??1???????????00?1010100??0?00?00?0????????0?01??0???00?0?????0?????00000?00?101000??00?0??010?11001001000100000110??00??0?101???00000???0000?????????000????????00101???0?1?1?010?????0??0000??100??0101100?0??0??0??10????????

*Alvarezsaurus_calvoi* ???????????????????????????????????????????????????????????????????????????????????????????????01000???0???0?20?0?2012????????????000?00????????0??1?0000101?00?2??????????????????01?000??0??0??11000000??00????????1???0?0??00?0?????0??????????????????????0????10?????????????????????????????????????????????????0100???0???????????????2?000?????0?0?0????????????????????????????????????????????????0??000?00???2????00000000?000???00?02???0?????????????0????????0??????????00??1????????????????????????????????????????????????????????????????????????????????????????????????????????????????????????????????????????????????????????????????????????????????0???????0001?00????????00?10?0?0???????????000?000?????????????????0??00?????00?????????1??0??0??0????????1000???????10?????101???????1????00?????????00000???????1???0?000?10??????1???0?0??0??0????????????????110?0?0??00?00??????????

*Patagonykus_puertai* ???????????????????????????????????????????????????????????????????????????????????????????????1?01?112????012010?2?2??????????????00210???210?????1?0???1????1010?????20????21202??11000000000001210??0????001??0???????0?????????0????0?????????????????????0?????01?????????????????????????????????????????????0??0100???????????????????20000?000?00??????0?0?0000??0?0010000???0??????????1???????????0???0????100200000000000??00?0???????10??0???????????????????????????????????0??????????????????????????????????????????????????????????????????????????????????????????????????????????????????????????????????????????????????????????????????????????????????????????????00001?01100?0?????000?1100??????11???0?0?01?01?1?000?10??1????0?00?????0????00?000?0??1???0??1001?101??1?0?1111111????????????0?00000??0?????00??0000?00?0?00??1?????????10??1???0???0????1?????1??0????0?????0100??????????

*Achillesaurus_manazzonei* ????????????????????????????????????????????????????????????????????????????????????????????????????????????020???2??????????????????????????????????????10??01?2???????????????????1??????0??1?01000??0???00????????1???????????0?????0??????????????????????0?????????????????????????????????????????????????????????????????????????????????????????????????????????????????????????????????????????????0???????????2????00000000?0??0??0??????????????????????????????????????????????????????????????????????????????????????????????????????????????????????????????????????????????????????????????????????????????????????????????????????????????????????????????????????????????????????????????????????????00?0??????????0????????00?0????0??????????????????????????????11????????????????11?????????????0????????????000???????1???0?00?????????????0?????0???????????????????????????????????????????

*Mononykus_olecranus* ??????00???????112???????????????????????????????????100????????????????????????2????00???????1?1?11102001???2?10?2??????1???1000?000200030210?02201100??1????1?201000?2?????3??0??0210100111011212000030000001??00???1??0?0??00?0?0???00??????????????????????????00011???????????????????????????????????????????0??010?0?????210???00?????2?010?0?0?0?010?011?0?0001000?001000000000??0000???30000???0???0??00???????2000000000000??0000??0?00?0??0??000???????01?0?????0?0????????11102????????????????????????????????????????????????????????????????????????????????????????????????????????????????????????????????????????????????????????????????????????????????0???????1001001000????????1010100000100?????011???0???01?010000010101?100000100????0??????00??1??0???021?1110010111111111101011???????0100000000000?000?0000??000010000?000?10????????100?10100?0?01???1?????1???1?0???????0?00??????????

*Shuvuuia_deserti* ?00101002?010111120110001011?01111?10020100001010010010???1110?00101001002100??0211??000??0111111011102100?0?201012012110100?1000?00020003021??0220110000101001?201000?2002203??03?02101001110112120000300000011000001100000010000?00??001000000001?010??000000000?000100000000?101000?10000110?0111000??0[12]0????0100??010000[01]0?0210?0?00?????20010?000?010101011?0?0001000?00100000?000?????????30000??000??00?000?00?1020000000000000?0000000?00100000?0000020?0001?01?1010001???????1110200?000?000??0?1??0000?0?00000?00000100???0??0?00??01?0????0?????0??000??00?0200?000000??0?0?0??00?000?001?0?0???????????????????000000000????????00000?000?0??000000??00??????0?0???????00010010001011?0001010100000100??0?01110000???01??1??????????0???0????00001????1??00??1??00110210?110010?1111111??0?01002?0??001?0?0000000?000000000??000?????00000010101?0??0?00110????00?1101?0?000???0110?0???000?00?00???????

*Albinykus_baatar* ??????????????????????????????????????????????????????????????????????????????????????????????????????????????????????????????????????????????????????????0????????????????????????????????1101??121100300000?????????????????0000?????0?????????????????????????????????????????????????????????????????????????????????????????????????????????????????????????????????????????????????????????????????????0??????0????????000000000?0000000?00??0????00????????0???????????????????11??????????????????????????????????????????????????????????????????????????????????????????????????????????????????????????????????????????????????????????????????????????????????????????????????????????????????????????????????????????????????????0?010000??00???????????00??1???????????????????????????????????????????????????????????????????????00000?10??????????0??????????1???????????????0?????????????????????

*Parvicursor_remotus* ????????????????????????????????????????????????????????????????????????????????????????????????????10?????????1??2????????????????????????????????????????1????2?1000???02203??03??2??100011?11211000030??00?????????1??????????0?00??0??????????????????????0?????0??????????????????????????????????????????????0??0?00???????????????????????????????????????????????????????????????????????????????????0????????1?2000000000000??000?000?00?0???????????????0???????????????????11?????????????????????????????????????????????????????????????????????????????????????????????????????????????????????????????????????????????????????????????????????????????????????????????????1000?????00?????????????????????????0???01??10??00?0?01??000??100???????????00??1????????1?111???????????11100010????????????0????????????0?????000??00000000010??????????0???1?0????1???1?????????????????????????????????

*Albertonykus_borealis* ???????????????????????????????????????????????????????????????????????????????????????????????????????????????????????????????????????????211?????1?0????????????????????????????????????????????????030?????????0???????????????????????????????????????????????????????????????????????????????????????????????????????????????????????????????????????????????????????????????????????????????????????????????????????????????????????????????????????????????????????????????????0??02????????????????????????????????????????????????????????????????????????????????????????????????????????????????????????????????????????????????????????????????????????????????????????????????????????????????????1??????????????????????????????0???00????0??????????????????????????????????11???11??1???????????????????????????????????????????0????0???????????1??????????????????????????????????????????????????

*Bonapartenykus_ultimus* ????????????????????????????????????????????????????????????????????????????????????????????????????112??0????????????????????????000210???????????????????????01????????????212?2??1????????????????????????0???????????0?0???????0????????????????????????????????0?????????????????????????????????????????????????0100???????????????????20000?000?000??10???????????????????????????????????????????????????????100??????????????????????????0?????????????????????????????????????????????????????????????????????????????????????????????????????????????????????????????????????????????????????????????????????????????????????????????????????????????????????????????????0?1000??1?????0?010?010??????????????????0?0?01?????????????????????00?????00???????????0?????01???011?????????????????????????????0??????????????????0?????0????????????????????10????0????????????????????????????????????????

*Ceratonykus_oculatus* ?0010???2??10?????????????????11???10?201?0?0?0100??0?????????????01?0??????????????????????????1?1??????????????????????????10????0?2???0?????????????????????????????????????????????1????????212?0003???00????????????????????0?????0???????????????????0???????????????????????00???????1????111????????????????????????????21?????0??????0000?0???01???????????????????0????????????????????????????????????????????????00000000??000?000?00?0????????????????1????10?0???????????????0??0?????????????????????????????????0?????????????1?0????0?????0??000??0??02???0?0000??0?0?0??00?000??0????0???????????????????0??0?00??????????0?0????????????????????????????0???????0001?????????????????????????????????????????????????????0?0??1000???????0??????????????????1?????1?00?0?????????????1???????????????????????????????????????00?00001???????????01???????????????????????????????????????????????

*Linhenykus_monodactylus* ????????????????????????????????????????????????????????????????????????????????????????????111?101?1??1?0???2010020?2???????10?0???1??????????0?21110??????????2??????????????????0?1010001????2?2?0?030?0000???0????1??????????0?????0???????????????????????????01??????????????????????????????????????????????0??012?0?????210????0?????20??0???0?0?0???????????????????10000???0??????????31??????00??0?????????1?2000000000000??000?000?001?????0?0?????????1???????0?????????????02????????????????????????????????????????????????????????????????????????????????????????????????????????????????????????????????????????????????????????????????????????????????0???????0001?01000?????0??????1??????????????????????????0?0??00?0?0???000???00?0??????1??0????????????1011????????1111?1???0??????????????0????????00???????????01??00?00001???????????0?1?110?0????????????????????????????????????????

*Xixianykus_zhangi* ????????????????????????????????????????????????????????????????????????????????????????????????????102?00?21201??0???????????????????????????????????0?01?1??1?200000?20022?3??03?02?010011101121211??3???000???????11???????00?0?00??0??????????????????????0????????????????????????????????????????????????????0??010000????????????????????????????????????????????????????????????????????????????????00?000000?1?2000000000000??000?000?0?????????????????????????????0???????????????????????????????????????????????????????????????????????????????????????????????????????????????????????????????????????????????????????????????????????????????????????????????????????????1000?0??????????????????????000110000???01?010??00?0?00010?0??????????????????????0??1???1011????????????111??11?????????????0??????????0?0000??000010000000001??????????0????100????1???1?????????????????????????????????

*Nqwebasaurus_thwazi* ?0????????????????02????2010????????0100?00??1?????0?00????????????????????????02????1??????00?11001???1??????????????????????????0102?000??0?00?10110??????????????????????????00?????0?0000100010000000?000?1??00??????0?0?0?????0????01?00???00?????????????????10200?????????????????????????????00????????????????????????0?????????????20????0?0???0101?0??????10?0???????????????????????00000??00000??????????00????????????00?0????00??0?0??00000????????0??????0?0??????????0?001?????????????0?0??????0???0??????????0???0??????0??????????????????????????1??????????????????????000??0??0?0??????????????????????00???????????????????????????????????????????0?????????????????????????000??01?0?1?0?0????????????????????????0????00?0???00?????0?111?0???1??0??012?????100??00010000100001???????0?????????????0?????????????????0?000010??????????0???????????1?1??????????????????????????????????

*Shenzhousaurus_orientalis* ???0???????????????21000??10?0??1???000??00?000?0???????????0?00000000??0??0?1?12?0??1????????????000??1???0???00000???0??????????????????????????010000000??11000100101?000001100??00001?0??????????????????0??1??1?0011?????00???0010??10000010????????00??0?00???0???00?000????????????????????00?????0??????0?00???100?0?0?0?????????????????????????????????????????????????????????????????0?????0?00000?00000?0002?000?????????????????????????00?????????????????00000???????????01?00??00000??0?0000?00?0?0000000000??00???0??0??1???1?????????????????????????????????????????????????????????????????10???????0??????????????????00?00??00?00?0????0?????????????????????????000???????00?????????????????010100000?0001?00??0?????????????????01?????0?10??00??1??00?2?0000????????100?0????????00?10????00????????00000000???00?????????????0???0??0???1???????????1???000?10?0????000?000001?-????????

*Ornithomimus_edmonticus* ?00010?1101101?101021000?0101010110000000000000001000000??????00000000010?1001?1???????0?001?1011000011100010100000000100100??????01120012000000?20200100000011000110101100000110010000011000100010000020??00?011111110121110000?0?0010001000000001?????0001?000000002100000000?????????0?0?10???000010110??????00000?010000[01]0?0??????????????0??0?0?0?0?01?1001?0?00?000???0000000??00??????1??00000??0000020?000?000002000000000000?0??0?????0??0??000?1???????0???0??0000000????????00010000?00000?00?0000?01?0?0100000??0??00???0??0001??01?0????000001000000??01?00????0000101?0000??000000??0????0????????1??????????1????????????????00210???0?00000?010????????????010?00?00001000011???????????????????10?00???1?????????????????????????1???????0111100111111111?11000???????100??0???00?01????101?0???1??0?0000?10000000000????0000000000000100?0????????1???????????10?0100?100????0?0???????0?-0???????

*Archaeornithomimus_asiaticus* ??????????????????????????????????????????????????????????????????????????????????????????00?101100001110001?100000000?0??????????010200?20000?0110[12]0???000?01100011010110000010201000001100??000?0000020??0000?10??10??1?1??00??0?001000??????????????????????????????????????????????????????????????????????????0??01000?[01]0???????????????20?00?00000?0?0??01?0?0010000?000000000000010000???00000??000??00???00000002000000000000?0000??00?0?10?000?????????????0??????0?0????????00001????????????????????????????????????????????????????????????????????????????????????????????????????????????????????????????????????????????????????????????????????????????????0???????0??1?00001?00??00000?00020010?010?010100000?01011?00000000000?000001000?????001?101?001?11?0????000010000001100001??011???????110?000001100000000000??000000000?000?10??????101?0?1?000?000????1?????100011??0?0??00000?-????????

*Anserimimus_planinychus* ??????????????????????????????????????????????????????????????????????????????????????????????????????1?????????????????0??????????1?200?2????00?20200100000011000110101????001?001????????????????00002???00????11??10??1?1??00?0?001000?????????????????????????????????????????????????????????????????????????????????????????????????????0????????0??1?????????????????????????????????????01000??00000?????????????????????????10000??00??0??0??00?1??????????????????????????????001?????????????????????????????????????????????????????????????????????????????????????????????????????????????????????????????????????????????????????????????????????????????????????????????????????????????????????10?0??1?1?????????????????????????1?0??????????11011011001??1?0????????100???01?00?????????????????????000?10?000?????????????????????010??????????????????????????????????????1?0?????????-????????

*Struthiomimus_altus* ?00010?110??0??1010210002011101?1100000000000000010??0001?01??0001000001020001?1?????????001?1011000011100010100000000100100??????011200120000001201001000000110001101011000001?0010000011000100010000020??000011111110121110000?0?0010001000000001??????00?1?0000??2210????0???????????0?0?1????000?????0?00???00?00?0?0000[01]0?0?????????????200?0?0?000?0101001?0?0010000??00000000000?100000??00000??0000000?000000000200?00000000000000??00?021000000?10022000000?0??0?00?00?????????0010000000000?00?0000?01?0?01000000000000???0??0?01??01?0????0000010?0000??01?0000??0000101?00?0??000000??00?0?0???????????????????1000011010?00????00210???0?000000000????????????00??00?00001000011?????000000000200?110101?10100100?01011?00000000000001000100000111001111111?1?110000??000010000001100001000110100???1??0?00001100000000000???000?000000000100?0?0?101?1110000?0000?1010?0???00011?0000?00?000?-????????

*Gallimimus_bullatus* ?00010?110110101010210002011?0101100000000000000010000001?01000000000001020001?1?????????00111011000011100010100000000100100??????0112001200000??20100100000011000110101100000110010000011000100010000020??000001111110121110000?0?0010001000000001?????000110000000221000?00000001000000?0000??00000?0?10200??100000?010000[01]0?0?????????????20010?0000000101001?0?0010000?0000000000000100001??00000??0000020?000000000200000000000000000?000?021000000?1002??0000?00??0000000???????000010000000000??0?0000?01?0?01000000000000???0??0?01??01?0????000001000000??01?0000?00000101000?000000000?000?0?000???????????????0010000111100000???00210?0?000000000000000????????000000?00001000011[12]000000000000020011?0101010100100?0101100000000000000100?10001110111111011001?110001??000010000001100001000110100???1100000001100000000000??0000000000000010000?001010111000010000?1010?00?10001110000?0000?00-????????

*Garudimimus_brevipes* ?000?????01101????02?00020101000??00000000000000000?00001?0100000000000002?001?1???????0??0111????0001?10001?100??00?????10????????????????????????????0000001100????????????0??001000001?000?000?000001000000?01??1110?2?1?0?00?0?0??000100000000??????0000100000010???00?0000?001000000?0000??0000010110000??000000?010000???0????????????????????????????????????????????????????????????????????????????00?0000000002000000000000000000000?021?0????0000????00???0??001000????????00???0000000000002?0000?00?0?01000000000000???0??0?01??01?0????000001000000??01?0000??00000000000000000000??00?0?000??00?010???????001000000010???0???00210???000000000000000????????0?0?00?0000??0001110000000????????????????010100100?0101?0000000?000000100?10001111??????001000?1?0001??0000???????????000010110100??????000??????????000000??0000000000000010000?0010??01??0011??00?111?000?1??011??0?000000000-????????

*Pelecanimimus_polyodon* ?00???????1????????2100?2?1??0000??000000?0000??????????????0?000?0000???????000211??0001??000???0?0?111?00???????????????0??0?00???120????00??0?2010????????????????????????????????????????????????????????1001010????0?????0?????0????10000??0?1?0????00????0000?0?10???0?0??????????0?0?????????????????????00?????10??????0??????????????0??0?0?0???0???????????????????????????????????1??00000??00000??????????????????????????????????????0??000???????????1?????0?00???????????001?00??00010??0?0000?00?000000000000??00???0??0?01??01?00?0?0000011??000??????????????????????????00??0?000??????????0010000????0??????????????????0??00??00?????????????????00000000?10???????0?0??????????????????????0?01?????????????????????????????????????010?1??111??????0????0?2?0?????000001100??????????0?1101??????????0?000????????????????????????1???0??????1??????????111??1?00????????????0??????0????????

*Harpymimus_okladnikovi* ?0?????????????????2100??????0??????000000000000??????????????000000?0000????1?1200??1???0???1???0?00??10001?100000?00?001????????0?1?001200000000010?10??00???????1????????????00?00??????0??0?010000010??000001011????1?0???00?0?0???001??0000??1?0????00??0000??11?10000000????????????0??????????????0000??00?000?0?0000[01]0????????????????????????????10??01?0?0010000?000000000000???0?010?00000??0000000?0?0000?00?????00000000?00000?00??11??0000?0????????0???????000?????????0?001?000000000????0????0??0?0?????0000?000???0??0?01??0??????????????????0???1?00??????0????????????00000??00?0??????????????????????????????????????00210??000????????0????????????0???????000??0000?1??0????0000??2001?00?01?1??0010?????????0??????????00?0??0001100100011??0??0?11?00????000???000001000????011???0??01???00000110?000000000???0?00???0?000?100?01??1?1?01??00??00???1?????????0????0?0?????????-????????

*Beishanlong_grandis* ??????????????????????????????????????????????????????????????????????????????????????????????????????????????????????????????????010200120000????0100?????????????10???1??00??????000001100?10001000?01??000?0???1???0??1?1????????01000????????????????????????????210??????????????????????????????????????????????????0??0???????????????20010?0000000101001?0?0010?00?000000000000010???????????????????0??????????2000000000000?00000?00????0??0?0?0????????0?0?????????????????00?01??????????????????????????????????????????????????????????????????????????????????????????????????????????????????????????????????????????????????????????????????????????????????????????????????????????00000020011?????????????????????00?????0?00000?0???00?????00??1?0???0??1??????????1000000?1??00?0?011?????????????000100??00????????????00000000001?????????1???10?0??0??0???1?????????????????????????????????

*Sinornithomimus_dongi* ?0001?????1?0????1?21000??1010001?000000000000000000?000??0???00000000?00????1?1?????????0001?0?10?0???100?1?1??0?00?0?0?100??????010200120000?01101001000000110001101011000001?00?00000110001000?000?010??000001011100121?1??00?0?0010?01000000001?????00001000000?02100000000?????????????00??000001?1?0??????00000?01?000?0?0?????????????20010?00?00?0101001?0?0010000?0000000000??01000010?00000??0000000?000000000200000000000000000??00??1100?0000?????????000???000000????????0?0010000000100?00?0000?01?000100000??0?000???0??0?01??01?0????000??10??000??01?0000??000??????00000000000??00?0?0?0?????????????????10000????????????00210???0?000000000????????????0???00?00001?000?????0?0??000000200?100?01010100100?0?010000?000?00?0??1?0?1?00111?1001110?1001?11?00???00001000000110000100?11??00???110000000110?000000000???00000000000001000??0?101?011?000?0?00?101?????????????????????????????????

*Qiupalong_henanensis* ?????????????????????????????????????????????????????????????????????????????????????????????????????????????????????????????????????????????????????????00??1100????????????01100?????????0??0001000002???00????????1????????00?0?0???0????????????????????????????????????????????????????????????????????????????????0???????????????????????????????????????????????????????????????????????????????????20?0?0?00000?????00000000?0000?000?02????????1????????0??????????0????????00??????????????????????????????????????????????????????????????????????????????????????????????????????????????????????????????????????????????????????????????????????????????????????????????????????????????????????????????10100?00?0001??????????000?010001??0??????????1??110????0?????????????????????1??01??????????????????????????0000??000??0000?00001??????????01??????1???????1?????????????????????????????????

*Kinnareemimus_khonkaenensis* ???????????????????????????????????????????????????????????????????????????????????????????????????????????????????????????????????????????????????????????????????????????????????????????????0???00001???00????????????????????0?????0?????????????????????????????????????????????????????????????????????????????????????0??????????????????????????????????????????????????????????????????????????????0?????????0??????0000000000000?000?02?????????????????????????????????????00?????????????????????????????????????????????????????????????????????????????????????????????????????????????????????????????????????????????????????????????????????????????????????????????????????????????????????????????????????0???????????????000??00000?00??????????????????????????????????????????00???1????????????????????????????????0???0000???001???????????0??????????????1?????????????????????????????????

*Huaxiagnathus_orientalis* ?00?0??????????????0001???1???0?????00??0?????????????????????00?0010????????00010?1010?00??0???1????????0??????00???0?002010????000000000?00??010000000020?0??000100201?000012?0???0??????0??0?010000000?0?011??000????00?0??00?0?0010?0100000?0?1?00????0????0000??200???0000??????????????????????????????????00?0??1??0?[01]0?0????????0000020?00?0?0?0?010100??????10?0???0???????????????????010????000??00?00??00000??????000?0??00??0??0????100000000????????000????00000?0?00??000000?01??00000??0?00?0?00?10000000000???1??????????????????????????00?00???????????????????????????????????0?????????????????????????????????????????02?00???0?????????????????1?0?00???????0?0??0000??????0??0001100???1?0?000??00000??0????????????0?????0?0???0?0?0?00?101000??01?0?00?0?00001000?000??0???????0?????10?????00????0?0?0?0000????0???0????0000100?????????????????????000???10?100011?????0000?00?0????????

*Sinosauropteryx_prima* 000?0??????????????0001???1???0?????0000??????00??0???????????00??01???????1?0001001010?000?01??100????1?0?0????00?110000201??????00000000?10??010000?00020??????0100201?000?11?0?1?00?0??000?0?01000000000?011??0000??000?0??00?0?000??010000000?1?00?00?????000?0??20000?000??????????????0???????0101????????0000????0?00[01]0?0??????????????0?00?0?00??010?000?????10?0???00?00????????????1??0100???0000000?00000??0?2?????00??0??00??00?0???0100000000????????000?10???000?????????0000?01???0000??1??1????0?1?0?00??0??????0?????????????????????????00??0???????????????????????????????????0?????????????????????????????????????????02?00???0?????????????????000100???????0?0??0010??????0??00111?0???110?000??00000??0?0???0??0???0??0?00?0???0?000?00?11100???0000?00?0?0?001000?0?0?0??????010????1100???0000???0?000?00000??000???????0000100?????100?????????????000??????100011?0000000?100?0000000??

*Compsognathus_longipes* ?00?0???????????????00101?1??00???00000????000????000???????0?00000100?1?001000010010101000?01??1010???1?0?0???000?0120002010?????0?000000?10????0?000000??????0?01002010000012?0??????????00?0001000000000001100?00????0?0???00?0?0010001100000001?00000000?0?000??020000?0000??0?0??0??????0???0000????01?0??000000?010000[01]0?0????????00?0?20000?0?0?0?010100????0?1000???0???00??????????????010????0?0??00??0?000?00??????00??0?0000?00000?00100000000????????00????000000????????00000?001?001000?1?0100?0001000000000000??0???0??0?000?00?0????0000000?00?0??01?12????????000???????11?000?????????????????????????0??????????????????02000?00000020000?0???????1?0000???????000??0000?0????0???????01???1????00????0??0?0?0???0??????0??0000?0???0?000000?101000??00?0??0?000?0010???0?0????0????10????1100??0000????0???0?00??????00???????0000100?0010100??1?0????????001????0?100011010?0100????10????????

*Juravenator_starki* 0000???????????????00010??11000?1???00000?0000?0?????00?????0?0000010???0????000100101??00??0???1???????0???????00??100002010????000000000000????0000000000?01100???????????????????0?????00??????0?0??000000?10?000?0??0??0??00?0??????010000000?1?00?0?000??0000???20000?000??????????????0????????????0??????000?0????00??0?0????????[01]00002???0???????0101000?0?0010000??000?000?000???0?0????100???0000?0???00?000??2??00??????000??0?0000?00100000000????????00???00?0?00???00??0?0000?011000?00?01?0100?00?1?00000000000?00???0??0?00??00?0????0000000??000??01?10????000??00???????11?000??00?0????????00?????????0??????????????????02000???0?????0???0???????0?010??????????????????????????0000??00??1?0?000??????0????????0??????0?????0?0???00000?0??101?00??00?0??0?0???0????0?0?0??0?0?????0????0?0?????000???0?000?0???????0??????????00100?0?????00?1??????0???00????????0??110?????0??????0????????

*Sinocalliopteryx_gigas* 0000???????????????0001??0???000???00?000?000??0??????????0?0?000001???????1?00010010101000?01??10?????1?0??????00??1000020?0?????0000000??00??0100000003?0?0??000110201?000012?0?????????00???0??000??0000?011??000?1??0000??00????010?01?000000???00?0000???00000??20000?0000?00?????0????00?????0????????????00000?01000?[01]0?0????????00?0?20000?0?000?010100??????10?0????0??00???????????1??010????0000000?0000000002??00?0????00?0??00?00??0100000000????????00??10?00000???00??00?000?01???0?010?0?0000?000??0000000000???0???0??0?00?????0????0????00?00?0??0???2?????00???????????11?0????0????????????0?????????0???????????????????2?00??00?00????????0?????1???0000??0??000?00010??????0??000010????1?0?000??00000??0?0???0??????0??0??0?0???0?000?00?100000??01?0?00?0?0?0010?0?0?0????0?0???0??0?0?00??0??0???00?000?0000????00????????000101?????10???1??????????000??????????????????????????????????

*Mirischia_asymmetrica* ?????????????????????????????????????????????????????????????????????????????????????????????????????????0??0?0???????????????????????????????????????0????????0001?02000???012100100010110????0?????????????1?????????????????????0010????????????????????????????????????????????????????????????????????????????????1?0??????????????????????????????????????????????????????????????????????????????????0???00?0?000?????????????????????????????????????????????????????????????????????????????????????????????????????????????????????????????????????????????????????????????????????????????????????????????????????????????????????????????????????????????????????????????????0???000??????????????????????1??????0?0?00?000?1??0?0??????????????????????0??????0??0????0????0???????????1????????????????????????????000?0????00??00??????????????????0??????????0????1?????????????????????????????????

*Ornitholestes_hermanni* ?0002???0?0?00?1???0?010?01110001?100101?0??1000001?01011????0000001000?0?000010?00101001?????011?0101210000??000?0010??1???????????????01?00?????????00001??1?0?0100101000001??0?1???????0????????00?00????00000?0010?00?0?0000?0?0000?01000000001?01?00001000002?112000000010?0??000000?0?00???0000?0?0000???000000?010000[01]0?????????????????????????????????0?0?0010000?000000000000??0??0????1000??0000000?000?00000?0000?????????0000?000?001??00?0????????00??0???0001?0?????????00??00?1?0000000000000000?000000000??00??0???0000?000?00?0????0000000?0000??01?0000?00?0000100??00001?000?0001100?00000?01000?????000000000000???0???02000000000020000?00000?00000000???????0001000002?10?00?0??????10010?01??000000000???0100????000????0?00?0000?0000???????????001??1010?0000???0???0????0?00??001000000?00???000000???000000??000?????????0?1000?1001?0001????0??0?1010?010001??0?1??0?00000?0?10????????

*Coelurus_fragilis* ?????????????????????????????????????????????????????????????????????????????????????????0??010010000121000?????0?0002????????????0?0??0?10001????0??????????????????????????01100?000001?0?0000110?0?0????0?00??00???0??????????0?0????0??????????????????????????11200??????????????????????????????????????????????01000??????????????????2?????????????01000?0?0010000?000000000000010000???0????????0000?????????002000000000000??000???????????0000???????????0??????0???????????0???????????????????????????????????????????????????????????????????????????????????????????????????????????????????????????????????????????????????????????????????????????????????0???????0000100002?????0?000?0??100100????????????0?0001?000000000?0000?????0????????????1??00??????????0000??000???0??00?0100????????110000000000?00?????????00000??00000001?????????0?1?0?11221111?????????101011?100??????????????????

*Tanycolagreus_topwilsoni* ?000????????????????00?0??????0???00110????????0?00???????????????????????10?00??????1?????????????0011?000?????0????0?0??0???????000000000011?1000000????????????????????????110010000011000000010000000000000??00???0??000?????0?0???00??????????????????1???1????[0 1]2000???0???????????????????00??0?0??????????0?00?01000??0?0?????????????20000?0000000101000?0?0010000?0000000000000100000??01000??00000??????????002000000000000000000000?00100?00000????????0?0?????????????????00000?????001000???????????????????0??????021000000?000?0?????????????????0??0??00???????0?0?000??00???????????????????????????????????????????????????????????????????????0??????????????????????00001????????0011001001000000??????????00???000000000000000000?000?????00100000000??0??0?0?100?1000000000000101000?????????????0000000000?????????0?0000000000010?????0??0?100101221011???1?????????????????????????????????

*Tugulusaurus_faciles* ????????????????????????????????????????????????????????????????????????????????????????????????????????????????0??????????????????????????????????0?0?????????????????????????????000?0?100??00110?0?1????0?????0??????????????????????????????????????????????????????????????????????????????????????????????????????????????????????????????????????????????????????????????????????????????????????????????????????2000000000000????????????????????????????????????????????????????01??????????????????????????????????????????????????????????????????????????????????????????????????????????????????????????????????????????????????????????????????????????????????????????????????????????????????????00?????????????????000000000000?0???????0?????????0???????????????????????????000001???1????????????????????????????????????0?????00??????????????????0001??1????1?????????????????????????????????

*Zuolong_salleei* ?0000??????????????0000010111?0???000?0000001100000?????1000?????????????????00010?101??1?010001000????1???000000?0000??????????????????000?0??????0?????00?0100?????????????01100110000110?00?0???00010?0?00?????0?0100??????00???0???0011000000??0???00??1???0??0002000??0000?????????????00??0000000??????????010???10??0?0????????????????????????????????0??????10000?000000000000010000???????????????00?0?0?00?0020000?????????0000?000?0?1???0??????????????0???000100????????0??00?????0000000000000?00000001000???????1???0??00?0000??????????????????0?????0000?00000?0?000??10??0010?0001101?00000????????????????????????????????????????????????0??????????100???????00000??0???000???0??????00000?????0?00000?0?0?0??0000000000????000?000?00??0????0?????000??00?0???0???????????000011?????0?0??0000?0?00000??00???0?0???0000000????00??0?????1?0?10??000??01?0?01?????????????????????????????????

*Bicentenaria_argentina* ???????????00?1??????0?0??????00??00?????????????00????????????????????????0?000000001????????????100?????????0?????????????????????0?00???????????0?1????0??10????????????????0???00000?10???0?110???????0??????????1????0?????????????????00??0???0???????????????1???0??????????00????????0??????00???????????0????010????0???????????????2???0???0???0?????0?0?001??????00000000?0??????????????????????0??0???0????2000?00000000?????????????0?????????????????????0????????????????00????????0???????????0???00??0???????????????????????????????0?0000000???????????????00000000?1?????????????????????????????????????????????????????????????1?2?00??000??0??????0???????????????????????1?00??0????00????????0??0?????????0000?02???00?0???????00????????0?????00??????0???00???0???????00???00?????0??????00??00?0??????0??0?????00?????00???????????0?????0?0??0?1?0????????????????????????????????????

*Kileskus_aristotocus* ???????????????????0?01120111????????????????????????????????????????????????00000?101?????????????????????????????????????????????????????????????????????????????????????????????????????????????00?00??0??????????????????????????????1?0000?0???0??00??????1????????0??001??????????????????????????????????????????????????????????????????????????????????????????????????????????????????????????????????????????????????????????????????????????????????????????????????????????????????1011201???000?0000?0000?0?????????????????????????????????????????????????????????????????????????????????????????????????????????????????????????????100010?????????????00???????????????????????????????????????????????????????????????????????00?????00???????????????1??????0????????????????????????????0??????????????????????????????????????????1?????????????????????0????????????????????????????????????

*Guanlong_wucaii* ?00020?000000?010[01]100011201111000?001100000010000000000?10????00000010?00??200000001010?100?000000000111000000000000?0???00???????000000100000?0100000003000010000100200000001110010000011000?0011000010000000000000000000000000?0?0010001100000001100?00?11101102?1120000?0010????0000?000?00??0000000??0?0????00000?010000?0???????????????20000?0?????0101?00?0?0010000?000000000000010000???01000??0000000?0000000002000000000000000000000000100?0000020?20?000?00??0001000???????000000000000112010?1000?00?1000001010000?003??0000??0??00?10001000001100010??01?1000?00000000000000?1000011?000000?00000?????????????020?01000100000000000????0?000000010???????010100???????000010000210000?0?0000010001000000000000001000000?0110000000100000?0000000?0001100000100?010010?1000100000000000010000000000?00100100000000000000000??0000000000000010100???1?0011?1001??1000001?????????????????????????????????

*Sinotyrannus_kazuoensis* ???????????????????0?01???101?????????????????????????????????00??0?1????????0?0000101??????????????0??????????????????????????????????????????????0??00??0?0?000????????????????????????????????????????????????????0??0?????00?????????1?0000?0??100??0?????11????????00?001?????????????????????????????0????????????????????????????????????????????????????????????????????????????????????????????????00?000?0?0???????????????????????????????????????????????????????0???????????00??????01?2?1??10???0??1?00????1?????????????????????????????????????????????????????????????????????????????????????????????????????????????????????0??????????????????????????0??????????????????????????????????????????????0001?????????????????????????????0?0??????0?????????????0?1????????????????????????????0??????????????????????????????????????????????????????????????00???????????????????????????????????

*Proceratosaurus_bradleyi* ?00?2??????????????00011?01111000?00??????????00?00???????000?00000010?00?120000000101011??????????????????????????????????????????????????????????????????????????????????????????????????????????????????????00??0????0?0??0???????????110000?0?1100?00?11?0?1?2??????00000100000?????????00???0??00??10?0????00??????????????????????????????????????????????????????????????????????????????????????????????????????????????????????????????????????????????0???????0??????????????????00?0010112?10?1000?00?1?0000101???0????????????????0?1000100000110001???????0???0???00000000??0?0????????????0000??000?????0??0?0??????00100?????0001???10?00000001000?????11000???????????????????????????????????????????????????????????????????????????????0?00????????????1????0?0????????????????????????0??0010????????????????????????????????????????1?0?1?????????????????000????0????????????????????0????????

*Dilong_paradoxus* ?0002??000000???0?100011??1111000?00110?000010000000100???????00000000?00??200000001010?1?0000?0001?0??1000?????0??0?0?0??0???????0000001100?????0000000?00??1??00110201?0?0001?00?000?0??00??00010000100?0000?00000?0?00000?000?0?0010001000000001100?001111?110201?20?000000????00000???0?0???0000010110000???000???0??00????0?????????????20000?0?0?0?0101000?0?0010000?0000000000000?0??0????1000??0000000???0?00000200000000000000000??00?0?1000?0000?0?20?00???010000100????????000000000010002?11?1000100?0?000000000000103??0100?00??00010111100001100010??00?1100??000000000010????00000?000100???????????????????020?010?010?0????01001??10?10002?00?11?????01110000000100000100001???????00000110001????10000?0001??000???011000000000000??010000000??11000????1?01?01001?????00??00???00???00000?00100?00?0000010?0?000000????00??0?000?0001?100???1??10112????0??0000???000?00011?1?2?000?0??10000000??

*Eotyrannus_lengi* ????????????????????000120??11??0???110???????????0?????????0?000?0?0??1???20000000101012?00?????01?0??1??????????????????????????000000010????????0?0?????????????????????????????????????????0???00000?0?00???00?0????00?0?????0?????0?1?000?00??10??0121???111?0?211?0000?0???????????????0??00?00100???00??0???0??010?0??0???????????????200?0?0?000?0?0?0?0?0?0010000?0000000?0000???????????00???????????????0??????????????????????????????0????????????????????????1???????????0?00?????1?00101???????00?0?01????01000?1010??1??0?00?00????????????????0?????????????????????01?1??0??????????????????001??????0?00????????????????????000?1002110100??????????2??00???00???00????0??????????00??011011?000???????????????????????????01??00?00100?000?0???0??????1?0??0?00????1000??????0?00????????0000?00???0000100?00??0??????????0000???0?1????0??????011??????1???0?1?1???????????????????????????????

*Juratyrant_langhami* ??????????????????????????????????????????????????????????????????????????????????????????0?00?0000001?10??00000??0???????????????????????????????????00?00?01000011010110000011?01???0??10????0?????????????????????1????????00???0010???????????????????????2????11??????????????????????????????????????????????0??0100?0?0??????????????????????????????????????????????????????????????????????????????00?000000000?0?00?000??00????????????1?????????????????????????1?0?????????????????????????????????????????????????????????????????????????????????????????????????????????????????????????????????????????????????????????????????????????????????????????????0????????00?100011?00100?0????????????????100000011001000?????1100001????????????????????0??01??1??1?????000????????????0?????????????0000?0??????????000000??000??0000?????????????1??1??????11??0????1?????????????????????????????????

*Xiongguanlong_baimoensis* ?0002???00000?????1?000220???0000?001101000?21000000?0001??000???????????????00000?101??2000000000000111000????????????????????????????????????????????0?12?01000???????????????????0000110??????????????????0??????0?00?????000?????????1?000000???00?0?1121?2102011???0??0000?00?000??????00???0??000???????????00??0100??????????????????????????????????????????????????????????????????????????????????00?0000000??20000???????0???????????????????????????0???????000100?????????????0010?10001????1?00?01?0?01?0000100??0010?0100??0??0??0?????0???01000?0???0?0100???001?111110010????100?1?11??00?100????????????001001000010?1???????????????????????????????2?000??000000011000011????????????????????????111??001???????0111012???????????????00??0???????????1??1?010?10?????????????00??????000?0??00001???????????000000?????000000???????10????1?????????1???0?0?01?????????????????????????????????

*Dryptosaurus_aquilunguis* ????????????????????????20??????????????????????????????????????0?0?0??2???????0000101?1????????????????????????0???00???????????????????10???????10?0????????????[01]?0?0?2???????101?01000100010001000??2???0??????????0?0????????0?0???????????????00????????????????11????0???????????????????????????????????0?????????????0?????????????????????????????????0?0?0010000?00????????????????????????????????0????????002000000000000??000??0?????????????????????????????????????????00?01????????????????????????0??????????????????????????????????????????????????????????????????????????????????????????????????????????????????????????????????212?1?1??????????????????????????????????????????????10?????????????????????1?0???012?1?0111?????1????0??????0?????????????0????????????????000000????????????????00?1???00?????????00??0000?00001????01??????????0?1??00?0?0?????????????????????????????????

*Appalachiosaurus_montgomeriensis* ????????????????????0?0?201110??????120?????????????????????00000?000?0????????00001010?2???????????????????????0?0?00???????????????????????????????????????????000010120?10???10??01000100010001000002?0?00??????0????0????????0?00100?11000000?1???1?021??????????????000??0??????000?????0???????????0000???0????????????0??????????????????????????????????????????????????????????????????????????????00????????002000000000000?0000?000?00?????????????????????????????????????00?????1????????000?000?01?010111?101??1?0020?01?10??0?0???????????????????????????????????????????????????????????000000010100010?00?????????????????11?110??1?????????0??1?????2?0?????????????????????????0??????????????????????????????1?01110???111111111021?0000????????????0?????0?0???00???????????0000000???00??0?????0???????????????????00?0??00000001???0???????01?????1??00000??1???????????????????????????????

*Bistahieversor_sealeyi* ?1102?????????10?2200002?01110000?001201000?21100000??????????00000000?20??2?000000101012??0????????????????????????????????????????????????????????????????????????????????????????????0??????????????2???????10??00??00?0??0???????????1200000011?00?00213?0?112??????0000000?????????10??0????00001???0?0????00???????????????????????????????????????00?????????????????????????????????????????????????????????????????????????????????????????????????11??????????0???????????????????0?1011001000?1010101?01011101010101002010001001???10000??00000020000211000010000??1111111?1?1001011001211111?01000?010011111??????11?111101?10111111100?11212101100112?10??2?00????111?????????????????0111?1???????????????????????????011?????01101111?120010000????????????1??1?0?0???????????????????????????0??0????????????????????????????????????????11001??????1??????????000?11???????????????????????????????

*Albertosaurus_sarcophagus* ?1102??000000?10022000022011100000001201000021100000?00010000000000000020??200000001010120????0?10?001?100?0????000??0????0???????000000010000???011001031200110000001012001000110??0?00?1000??00100000200000001000000000000?000?0?0010?01200000011000?0021310111210201100000000???00000100?00??0000010010000??000000?010000?0?0????????0000020000?00000?0001001?0?0010000?000000000000010??0?????000???00??00?0000000002000000000000000000?00??0100000??020100?000??0??001100?0110?000000101110110010000[01]1001[01]100101110102011100211[01]1[01]1101?001000000100010201002001100100011111011111111101011001111111000[01]000111[01][01]1111000010110111111110111111100111212111100?12?10?121011???????1?????????????????1111102011111?1021111110211101??1??1???111111111122010000000??00000101?011010?100010?000??00?000000100?000000??0?00??110??00000000??0000?0000000001011001111010111????01000000110?????????????????????0????????

*Gorgosaurus_libratus* ?11020?00000011002200002201110000000120100002110000010001000000000000002001200000001010120?0000010000101000000?000000000000?00001000000001000000101100103120011000000101200100011010010001000?0001000002000000010000000000000000?0?00100012000000110001002131011121020110000000000000000100000??0000010?10000??000000?010000[01]0?0????????0000020000?00000?0001001?0?0010000?00000000000001000010??1000??0000000?0000000002000000000000000000000?0010000000020100?000000??00110000110?000000101110110010000[01]1001010010111010201120021101[02]1?010001000000100010201002001100100011111011111111101011001111111000100011111111??00110110111111110111111100111212111100112?10?121011???????1????1101????1?0?0111110201011111021111010211101?011?1?2?111111111?2201000000010000001011011010?100010?000010000000001000000000000?0000110?000000000??0000?000000000101100111101011110??0?0000001100???1??1?0???00????0?0????????

*Alioramus_*spp. ?01020?00000011002200?0?20111000000012010000211000001000100000000000000200?20??000010101?000000010000111000000?00?00?0??000??????????????????????????????120011000[01]001012?010??????001000100??0?01000?02??000??10??000000?0?0000????010??11000000110001?0213101?12102????000?00000000000100000??00000100?0000??00000??0100?0?0??????????????????????????????????????????????????????????????????????????????00?0???000??2000000000000000000?0??0?1?0????0020110?00???0??0011000???????0????00111?2????001110111110?0111010100120121001021110101110001000010200001000000101101112011111111001011001111111?101110011011101000110110111101101110111100111212111110??10000???000001000011110?101221011111?????????????????1111010??????1?1111121??00?111?12?0100000??????0???0?1?1?010??000???????????0????01?0000?000000?0????????????0000??0??00???0?000?1??1101111?1?1??1011??0?000?11???????????????????????????????

*Teratophoneus_curriei* ?1?0???0???????0??2??????0111?00????1201???????0000???????????00??000??????????000010101??1????????????????????????????????????????????????????????????0????????????????????????????????0??????????????????????????0????0????????????????1?0000?01?????????3?????????0???000????????????10??0???00?001???0?????0???????????????????????????????????????????????1????????????????????????????????????????????????????????????????????????????????????????????100????????????????????????????????????????0????0?01??????????????2?021?0001??10??????????0???01?1??????0???01??1?1??21?111?100111101211?????????????????????????011?1?120?1????1??1100?11?????????11????????10???????????1?????????????????1?01???1????????????????????011?1?1???????????????000??????????????????0?0???????????????????????????0??0????????????????????????????????????????????1??????1??????????00??1????????????????????????????????

*Daspletosaurus_*spp. ?11020?000000?1002200002201010000000120100002110000010001000000000000002001200000001010120100000100001?100?0????000??0????0???????000000010000???111001031200110000001012001000110??0100?1000??001000002000000010000000000000000?0?0010?0020000001100010121310111210201100000000???00000100?00???000010010000??000000?010000?0?0????????0000020000?00000?0001001?0?0010000?000000000000010??0????1000??000??00?0000000002000000000000000000?00??01000000?020110?000??0??00110000110?000000101110120011020110021110211110102101201210101[12]111111111111101101020000211001011[01]11211212111111100?111012211111110111001101111[01]100111110111101111111111100111222111101112?10012101100011101111021111???????111111?211?111?112111??1??111?1??1????2?11????11?12???00000??11000????1??1?010?1000???000?1000?0??????0?000000000???0011000?0????????????????????????111011110??1??????????000?1100?00001100???00??????0????????

*Tyrannosaurus_rex* ?12020?01100011002200002201010000000120100002110000010001000000000000002001200000001010120100000100001010000001000000000000?0????0000000010000???1110010312001100000010120010001101001000100010001000002000000010000000000000000?0?00100002000000210001012131012121020110000000000000000100000??0000010010000??000000?010000[01]0?0????????0000020000?0000000001001?0?0010000?000000000000010000????1000??000??00?000000000200000000000000000000000010000000020100?000000??00110000110?00000011111112001102101[01]0[13]01102111101021112010??10121011111011[01]1101111021010211001011111211[12]12111111100111121230111111111110110111111111111101102011111111111112112221211011121111121121111110111110211112111111111111021101111112111111021111111122112111111111112211000000010000001011011010?1000100000??0000000001100000000000100001100?10000000??00000000000000101110111101011110110100000011000001001000?0000000010????????

*Tarbosaurus_bataar* ??2020?011000?10022000022010100000001201000021100000100010000000000000020012000000010101201000001000010100000?1000000000000???????000000010000?011110010312001100000010120010001101001000100010001000002000000010000000000000000?0?00100002000000210001012131012121020110000000000000000100000??0000010010000??000000?010000?0?0?????????????20000?00000?0001001?0?0010000?000000000000010000????1000??0000000?0000000002000000?0000000000000000010000000020100?000?00??00110000??????00001111111200110210111301102111101021012010??10121111111011111011110210102110010111112112121111111000111212301111111111101101111111111111011020111111111111121122212?101112111112112111111?111?1021112??1??1?11111102110111?112111111021111111122112111111111112211000000010000001011011010?1000?0000001000000000110000000000010000110??10000000??0000000000000010111011110101111011??0000001100?00000000??0000000010????????

*Alnashetri_cerropoliciensis*

?????1?02-0????????1?000??11?0??????????????????????0???1????000??001???0????00021?--10?000110?11-001??0000012010?[12]002???10??0?0-?100200111????011011001?1110011002?00-20???022223-011010??0??00011000000--0001??000?01?00?0?[12]00-0-0?--0001000??0??-01?0?00???000?0?02000000010????????????0??????????????000--?0???0?010??000-0??00-0?0?????200?0-00000?0101000?0?0000000-00001011?000-?00?01??00000??000??00?00000000020???0000?0?110000-00000010??001-10??201??01?0?????1?0????????00001????011000??[12]101?0?00?0000??000???0???????????????????????????????????????????????????????????????????????????00000001000?????0??????????????????00?00??00?????????????????0?00?0??000?00001?00?0?0000?1?000111010000?000?0010000???0????0100000?0?00?00?000?0?0?0??0??01010??0000?1??2??100101010?010100???110???01100100000001000?00000000???0001??00?0000101???0?1?100??010?20001001??0???????????????????????????????

*Gobivenator_mongoliensis*

?001???12-1?1?00??????0?20100000110020220000110100?1?1001?0000?0000010?111?000?0211--010?001?1?11?0??????111???00010221121?????????00111001??????????????11?0100002212020111011?0???111?0???????011?0?12????10?01?000?1000001100?1-0010000000000001-000?-100000?0001[0 1]20??00000000000010000?000--?0100101?0??????000?0?0?100000-0??????????????0000-0?000?01110?0???????00???00?0?0??????????????????????????00-00??000001???????????0000000?2?20200000????????00??1?????111101????????00???0000????????2?01??00000001000001000001???0??0?01?00?-0----000?01000000--00-0000-000000?0???00?-??0000-001110001?01??010???0?0?000?00??000---0-???000?????0?110000000??????????00??????-00?????0000?????0?000111010??????????0?100?[12]10101??1???????????00???????0001?0?????011????1010?0?0000100????????1????01?100????0???0000????????????????000???????000?1?????0?10????00????00??000?00???????????????????????????????

*Talos_sampsoni* ??????????????????????????????????????????????????????????????????????????????????????????????????0?001?11???1??0???2??????????????????????00????????????????????02222?20??00??1??????1??????00?01000012100010????0?????????????11?001?00???????????????????????????[01]?????????????????????????????????????????????????0?10????????????????????????????????????????????????????????????????0?????????????????0?-???????0?1????0??00????0000002?202?????????????????1???????????????????00????????????????????????????????????????????????????????????????????????????????????????????????????????????????????????????????????????????????????????????????????????????????????????????????001?0??????????????????0??????????????????????????????00??0?000000???????????01??0?0???????10??????00????????0??11???????????0???????????????????00????????000010????????0?0??????????0??????00?0??0?11?0??1100000?0????????

*Philovenator_curriei* ???????????????????????????????????????????????????????????????????????????????????????????????????????????????????????????????????????????????????????????????????????????????????0211100?0??00011110121??01???????????????????11?????0?????????????????????????????????????????????????????????????????????????????????????????????????????????????????????????????????????????????????????????????????????????????????1??????????????????2?202?????????????????1???????????????????00?????????????????????????????????????????????????????????????????????????????????????????????????????????????????????????????????????????????????????????????????????????????????????????????????????????????????????????????????????????????100?0100?00?00?0???0?????????????1???????????????????????????0????010??????????????????????????????????00?????000?1???????????????00???????????????1?????0?????????????????????

*Pamparaptor_micros* ???????????????????????????????????????????????????????????????????????????????????????????????????????????????????????????????????????????????????????????????????????????????????????????????????????11??00???????????????????01??????????????????????????????????????????????????????????????????????????????????????????????????????????????????????????????????????????????????????????????????????????????????????????????????????0???00?02?????????????????????????????????????????????????????????????????????????????????????????????????????????????????????????????????????????????????????????????????????????????????????????????????????????????????????????????????????????????????????????????????????????????????????????????????0000??0????????????????????????????????????????????????0???????????????????????????????????????????0?1???????????0????????????????????????????????????????????????

*Linhevenator_tani* ?0????????????????1?????1????000??102?2??0002101???0100??1???????????????????0?011?010??0?????????01?????0??????????????????????????0??0001???????????????????????10?3????11??????????????0?????????0??211011??????????00???????11???100?????000??1??????10?0???00??121???????0??????????????????????????????????00??0???00???????????????????????????????01?0?0???1?1000???000101??000?????????????????????0???????????0?0???????????0000112??02???????00??????????????11????????????10?????10????1?????????????????????????????????????????????????????????????????????????????????????????????????????????????????????????????????????????????????????????????????????????????????????????????????????????????????????????????????????????????????????????????????????????????????????????????????????????????????????????????????????????????????????????????????????????????????0?0?????10????1????????????????

*Diuqin_lechiguanae* ?????????????????????????????????????????????????????????????????????????????????????????????????????????????????????????????????????????01???????????????????????????????????????????????????????????????????????????????????????????????????????????????????????????0??????????????????????????????????????????????????????????????????????????????????????????????11?0??001000000000??????????????????????????????????????????????????????????????????????????????????????????????????????????????????????????????????????????????????????????????????????????????????????????????????????????????????????????????????????????????????????????????????????????????????????????????????????????0??1??????10???????????????????????????????????????????????????????????????????????????????????????????????????????????00010???????????????????????????????????????????????0???????????????????????????????????????

*Shri_devi* ?????????????????????????????????????????????????????????????????????????????????????????011000110001121011[01]?100??1????1?1????????????????????????????00?211?0111?202202011112?100???1110000??0001??0110100100???????1????????00???00102???????????????????????????12??????????????????????????????????????????????00000000???-?????????????????????????????????????????????????????????????????????????????0000000000000000000000000?00?00000-01???????00?????????0???????1?1????????00???????????????????????????????????????????????????????????????????????????????????????????????????????????????????????????????????????????????????????????????????????????????????????????0001000100????0000????????????????0010000011??01???0000000?000000000000???????????02??0????1????0000???????????00?0?010???????000000??????????000000???0001?0000000010??????0??00???100???01???1?????1??011??0?0??0??00??????????

*Kuru_kulla* ????????????????????00?0??????????????[12]???????????????????????000?010??2?????0??00010100????0???1?01111?????????0??01[01]?????????????????????0?1?10?0001????1????????????????????100?011100?0?????????01101?0?????00?0?11?0?????001???????????????????????????????????2????00??????????????????????????????0?0?000???1?001000??????????????????????????????????????????????????0000???????????????0000????0???0??????00?000000??????????????0??????1???0?0?0???????????????????????????????00?????00001???????????????????????????0????00???????????????????????????????????????????????????????????????????????????????????????????????????????000000001020000????????????????????????????0100?????????????????0??010???????????0??????00000???????????0?00?00???????00??00?????0????0??????0?0?????0???0???????0000000????0?00??0???0?????0?????????????0???11??????1??1?0??????0????0?01??0??0?0???????????????????

*Suskityrannus_hazelae* ???????????????????1?012??111???????????000???????????????????00??000??1??12?0?000?101???????0?010000111????????0????0??????????????0?00????????????????????????????????????????10?000001100000001000002???00???0?????0???0??????0?0???0?1?000??0?1000?????????1????????00?0?0??????????????????????????1?????????00?001000??0???????????????2?????????????0??????????????????????????????????????????????????????????002000000?0?000?0000?000?0?????????????????????????0?1??????????00?????????0001??1??00???0?0?0110?0???????????????????????????????????????????????????????????????????0010010????????????0???????????????????????????????1??????21??1????11?????1?1000????????0111?0011?????0?????????????????????????????????0011?11??101?1110?21??0?0?????????????1??????0??000???????????00000?1????0??00000000??????????????????00000000?000?1?1?????1???0???011?0?1000?1????01??001?????????????0????????

*Moros_intrepidus* ????????????????????????????????????????????????????????????????????????????????????????????????????????????????????????????????????????????????????????????????????????????????????000011?0???00???0??2???0??????????0??????????0?????0????????????????????????????????????????????????????????????????????????????????????????????????????????????????????????????????????????????????????????????????????????????????20?0????????0???00??0???????????????????????????????????????????????????????????????????????????????????????????????????????????????????????????????????????????????????????????????????????????????????????????????????????????????????????????????????????????????????????????????????????????????????????0??10???0???????1001?????????????????????????????????????????????????0????????????????????????????????????000??0?001?????????????????????00????????1????11??????????????????????

*Timurlengia_euotica* ?????01001??????021?????20111??????????10000???????0??????????????????????1200?0000101?12????0?0?01001110???????0??000?????????????????????????????0?0??????????????????????????????????????????????????????????0???????0?0?00???????????1?0?????1??0???0???1?????????????????????????????00?????0??00????????????0??00100?????????????????????????????????????????????????????????????????????????????????????????????????????????????????????????????????00???00???0???0010????????????010??????????00??00??0100?01?100?????????????????????????????????????????????????????????????????0?01100?1????????????????????????02??01000?0???100??????????21?11?1??11??????21000????????01??00?11?????0?0????????????????????????????????????????????????????00????????0?????0??????10?1000?????????00????????0???0??000?00???????????????????????????????????0????1???????????????0???0????1??????????????????0????????

*Ypupiara_lopai* ?????????????????????????0?????????????????????????????????????????????????????02??--1?????????????????????????????????????????????????????????????????????????????????????????????????????????????????????????????????????????????????????????????-1??????????????????????????????????????????????????????????????????????????????????????????????????????????????????????????????????????????????????????????????????????????????????????????????????????????????????????????????????????????????????????????0??????????????????????????????????????????????????????????????????????????????????????????????????????????????????????????????????????????????????????????????????????????????????????????????????????????????????????????????????????????1????????????????????????????????????????????????????????????????????????????????????????????????????????????????????????????1???????????????????1????????

;

ccode + 2 15.18 23 24 26 37.39 44 62 65 71 73 88 102 107 110 113 116 118 120 122 129 145 147 151 153 160 162 165 167 168 170 174 177 178 180 194 196 199 216 221 231 233 234 237 241 249 251 254 255 260 261 264 267 269 278 286 291 298 308 315 318 320 325 327 346 350 358 363 378 383 384 386 392 396 400 408 412 414 416 419 424.427 432 433 442 444 445 458 461 474 481 484 487 493 498 519 539 544 555 556 559 577 578 604 612 621 627 630 631 634 641 647 650 664 668 669 683 693 697 701 710 711 714 726 727 762 779 804.806 817 822 841 842 883 *;

proc /;

comments 0

;

**2. Unstable taxa revealed by IterPCR procedure (taxon numbers in parentheses)**

a: *Balaur_bondoc* (5); b: *Tianyuraptor_ostromi* (8); c: *Zhenyuanlong_suni* (11); d: *Graciliraptor_lujiatunensis* (13); e: *Hesperonychus_elizabethae* (14); f: *Pyroraptor_olympius* (15); g: *Shanag_ashile* (20); h: 'EK_troodontid_IGM_100/44' (40); i: *Archaeopteryx_lithographica* (43); j: *Sapeornis_chaoyangensis* (50); k: *Pedopenna_daohugouensis* (72); l: *Epidendrosaurus_ningchengensis* (73); m: *Epidexipteryx_hui* (74); n: *Rinchenia_mongoliensis* (81); o: *Erlikosaurus_andrewsi* (88); p: *Neimongosaurus_yangi* (90); q: *Therizinosaurus_cheloniformis* (96); r: *Ceratonykus_oculatus* (107); s: *Archaeornithomimus_asiaticus* (113); t: *Kinnareemimus_khonkaenensis* (123); u: *Mirischia_asymmetrica* (129); v: *Juratyrant_langhami* (142); w: *Pamparaptor_micros* (158); x: *Diuqin_lechiguanae* (160); y: *Ypupiara_lopai* (166).

**3. Jackknife values after pcrjak procedure (taxon numbers in parentheses)**

**
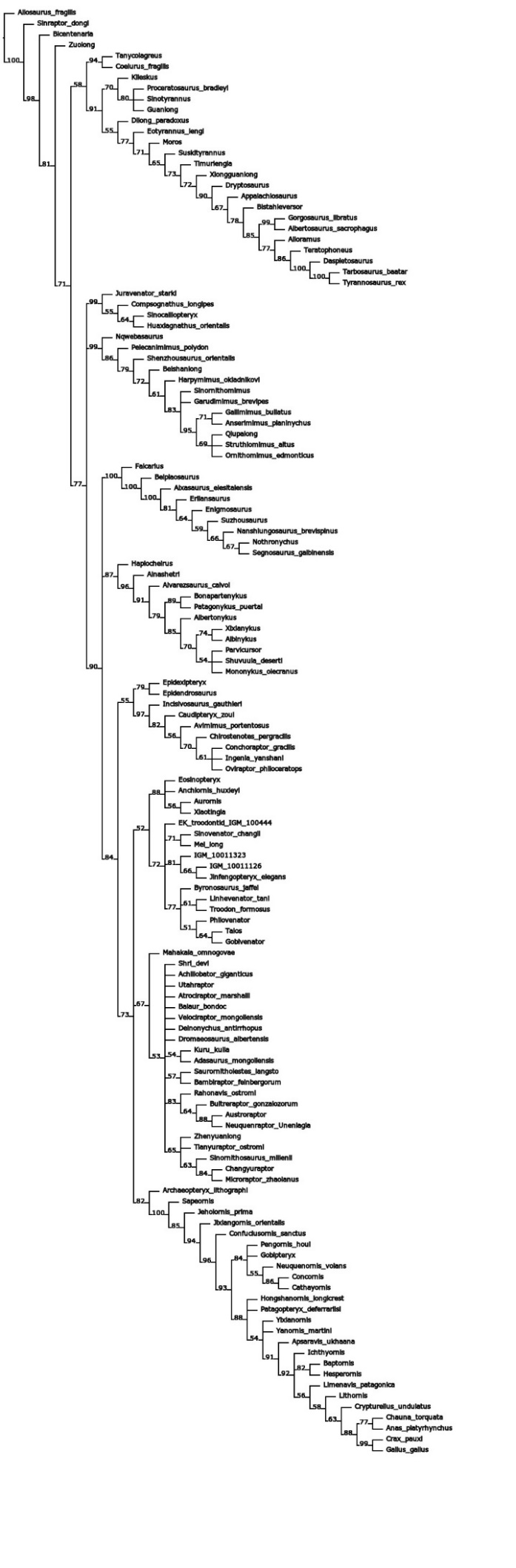
**

Figure S1. Majority rule tree (from 100 trees, cut 50).

Number of nodes in MRC: 105

Sum of supports in MRC: 7997

Average support in MRC: 76.9

Taxa pruned from MRC:

Groups of taxa:

**selected_unstable (group 28, 33 taxa)**: *Tsaagan_mangas* (6), *Graciliraptor_lujiatunensis* (13), *Hesperonychus_elizabethae* (14), *Pyroraptor_olympius* (15), *Shanag_ashile* (20), *Saurornithoides_mongoliensis* (27), *Zanabazar_junior* (28), *Xixiasaurus_henanensis* (29), *Sinornithoides_youngi* (31), *Vorona_berivotrensis* (56), *Songlingornis_linghensis* (57), *Iaceornis_marshii* (62), *Liaoningornis_longidigitris* (66), *Pedopenna_daohugouensis* (72), *Citipati_osmolskae* (76), *Microvenator_celer* (78), *Rinchenia_mongoliensis* (81), *Erlikosaurus_andrewsi* (88), *Neimongosaurus_yangi* (90), *Therizinosaurus_cheloniformis* (96), *Achillesaurus_manazzonei* (100), *Ceratonykus_oculatus* (107), *Linhenykus_monodactylus* (108), *Archaeornithomimus_asiaticus* (113), *Kinnareemimus_khonkaenensis* (123), *Sinosauropteryx_prima* (125), *Mirischia_asymmetrica* (129), *Ornitholestes_hermanni* (130), *Tugulusaurus_faciles* (133), *Juratyrant_langhami* (142), *Pamparaptor_micros* (158), *Diuqin_lechiguanae* (160), *Ypupiara_lopai* (166)

**all_unstable (group 29, 55 taxa)**: *Tsaagan_mangas* (6), *Graciliraptor_lujiatunensis* (13), *Hesperonychus_elizabethae* (14), *Pyroraptor_olympius* (15), *Rahonavis_ostromi* (16), *Buitreraptor_gonzalezorum* (17), *Neuquenraptor_argentinus*+*Unenlagia_*spp. (18), *Austroraptor_cabazai* (19), *Shanag_ashile* (20), *Saurornithoides_mongoliensis* (27), *Zanabazar_junior* (28), *Xixiasaurus_henanensis* (29), *Sinornithoides_youngi* (31), *Mei_long* (32), *Sinovenator_changii* (39), 'EK_troodontid_IGM_100/44' (40), *Vorona_berivotrensis* (56), *Songlingornis_linghensis* (57), *Hesperornis_regalis* (59), *Baptornis_advenus* (60), *Iaceornis_marshii* (62), *Limenavis_patagonica* (63), *Hongshanornis_longicresta* (65), *Liaoningornis_longidigitris* (66), *Pedopenna_daohugouensis* (72), *Citipati_osmolskae* (76), *Oviraptor_philoceratops* (77), *Microvenator_celer* (78), *Rinchenia_mongoliensis* (81), *Erlikosaurus_andrewsi* (88), *Neimongosaurus_yangi* (90), *Suzhousaurus_megatherioides* (92), *Enigmosaurus_mongoliensis* (94), *Nanshiungosaurus_brevispinus* (95), *Therizinosaurus_cheloniformis* (96), *Achillesaurus_manazzonei* (100), *Mononykus_olecranus* (101), *Shuvuuia_deserti* (102), *Albertonykus_borealis* (105), *Ceratonykus_oculatus* (107), *Linhenykus_monodactylus* (108), *Archaeornithomimus_asiaticus* (113), *Kinnareemimus_khonkaenensis* (123), *Sinosauropteryx_prima* (125), *Compsognathus_longipes* (126), *Mirischia_asymmetrica* (129), *Ornitholestes_hermanni* (130), *Tugulusaurus_faciles* (133), *Eotyrannus_lengi* (141), *Juratyrant_langhami* (142), *Philovenator_curriei* (157), *Pamparaptor_micros* (158), *Diuqin_lechiguanae* (160), *Kuru_kulla* (162), *Ypupiara_lopai* (166)

**unstable_within_replicates (group 30, 32 taxa)**: *Tsaagan_mangas* (6), *Graciliraptor_lujiatunensis* (13), *Hesperonychus_elizabethae* (14), *Pyroraptor_olympius* (15), *Shanag_ashile* (20), *Saurornithoides_mongoliensis* (27), *Zanabazar_junior* (28), *Xixiasaurus_henanensis* (29), 'EK_troodontid_IGM_100/44' (40), *Vorona_berivotrensis* (56), *Songlingornis_linghensis* (57), *Iaceornis_marshii* (62), *Limenavis_patagonica* (63), *Liaoningornis_longidigitris* (66), *Pedopenna_daohugouensis* (72), *Rinchenia_mongoliensis* (81), *Erlikosaurus_andrewsi* (88), *Neimongosaurus_yangi* (90), *Enigmosaurus_mongoliensis* (94), *Nanshiungosaurus_brevispinus* (95), *Therizinosaurus_cheloniformos* (96), *Achillesaurus_manazzonei* (100), *Albertonykus_borealis* (105), *Ceratonykus_oculatus* (107), *Linhenykus_monodactylus* (108), *Kinnareemimus_khonkaenensis* (123), *Mirischia_asymmetrica* (129), *Philovenator_curriei* (157), *Pamparaptor_micros* (158), *Diuqin_lechiguanae* (160), *Kuru_kulla* (162), *Ypupiara_lopai* (166)

**unstable_among_replicates (group 31, 42 taxa)**: *Hesperonychus_elizabethae* (14), *Pyroraptor_olympius* (15), *Rahonavis_ostromi* (16), *Buitreraptor_gonzalezorum* (17), *Neuquenraptor_argentinus*+*Unenlagia_*spp. (18), *Austroraptor_cabazai* (19), *Shanag_ashile* (20), *Saurornithoides_mongoliensis* (27), *Zanabazar_junior* (28), *Xixiasaurus_henanensis* (29), *Sinornithoides_youngi* (31), *Mei_long* (32), *Sinovenator_changii* (39), *Hesperornis_regalis* (59), *Baptornis_advenus* (60), *Iaceornis_marshii* (62), *Limenavis_patagonica* (63), *Hongshanornis_longicresta* (65), *Pedopenna_daohugouensis* (72), *Citipati_osmolskae* (76), *Oviraptor_philoceratops* (77), *Microvenator_celer* (78), *Erlikosaurus_andrewsi* (88), *Neimongosaurus_yangi* (90), *Suzhousaurus_megatherioides* (92), *Therizinosaurus_cheloniformis* (96), *Achillesaurus_manazzonei* (100), *Mononykus_olecranus* (101), *Shuvuuia_deserti* (102), *Ceratonykus_oculatus* (107), *Archaeornithomimus_asiaticus* (113), *Kinnareemimus_khonkaenensis* (123), *Sinosauropteryx_prima* (125), *Compsognathus_longipes* (126), *Ornitholestes_hermanni* (130), *Tugulusaurus_faciles* (133), *Eotyrannus_lengi* (141), *Juratyrant_langhami* (142), *Philovenator_curriei* (157), *Pamparaptor_micros* (158), *Diuqin_lechiguanae* (160), *Ypupiara_lopai* (166)

**4. Bootstrap values after pcrjak procedure (taxon numbers in parentheses)**


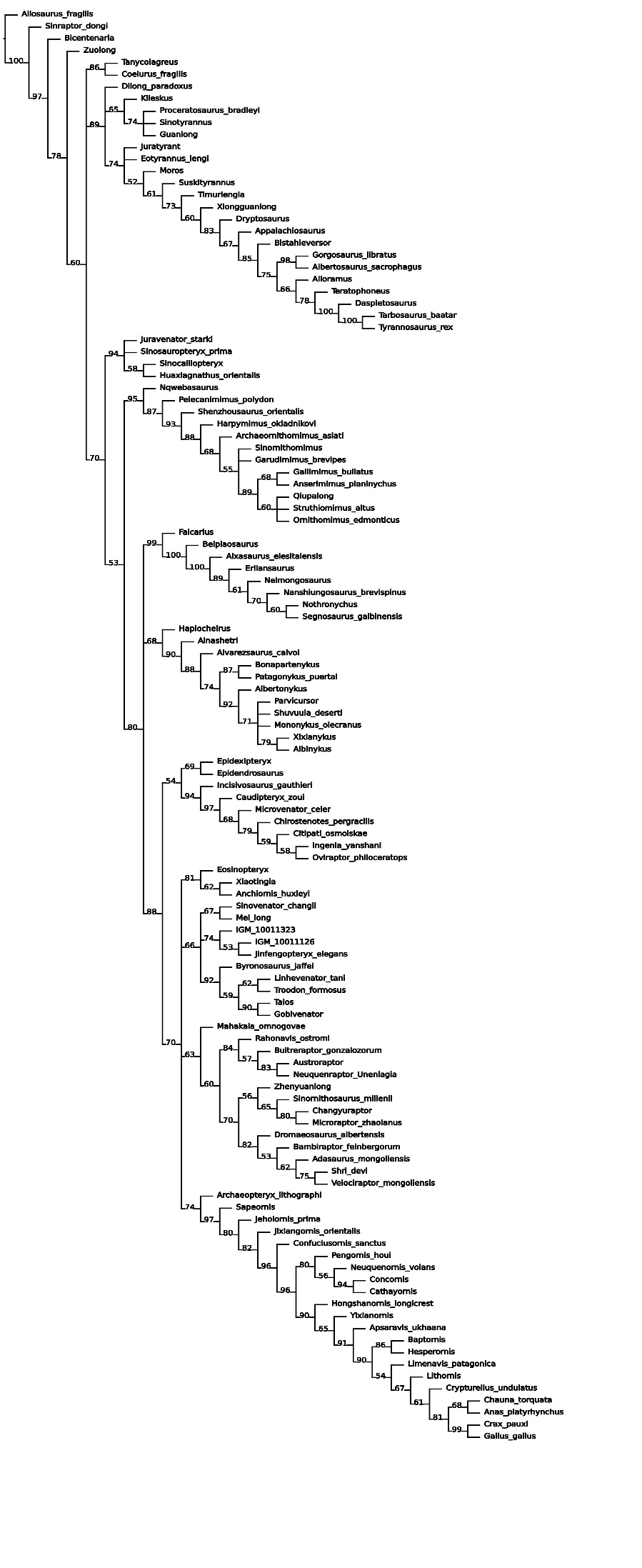


Figure S2. Majority rule tree (from 100 trees, cut 50).

Number of nodes in MRC: 104

Sum of supports in MRC: 7846

Average support in MRC: 76.2

Taxa pruned from MRC:

Groups of taxa:

**selected_unstable (group 28, 48 taxa)**: *Deinonychus_antirrhopus* (3), *Balaur_bondoc* (5), *Tsaagan_mangas* (6), *Tianyuraptor_ostromi* (8), *Graciliraptor_lujiatunensis* (13), *Hesperonychus_elizabethae* (14), *Pyroraptor_olympius* (15), *Shanag_ashile* (20), *Atrociraptor_marshalli* (22), *Utahraptor_ostrommaysi* (23), *Achillobator_giganticus* (25), *Saurornitholestes_langstoni* (26), *Saurornithoides_mongoliensis* (27), *Zanabazar_junior* (28), *Xixiasaurus_henanensis* (29), *Sinornithoides_youngi* (31), *Aurornis_xui* (36), 'EK_troodontid_IGM_100/44' (40), *Yanornis_martini* (47), *Patagopteryx_deferrariisi* (52), *Gobipteryx_minuta* (55), *Vorona_berivotrensis* (56), *Songlingornis_linghensis* (57), *Ichthyornis_dispar* (61), *Iaceornis_marshii* (62), *Liaoningornis_longidigitris* (66), *Pedopenna_daohugouensis* (72), *Rinchenia_mongoliensis* (81), *Conchoraptor_gracilis* (82), *Avimimus_portentosus* (84), *Erlikosaurus_andrewsi* (88), *Suzhousaurus_megatherioides* (92), *Enigmosaurus_mongoliensis* (94), *Therizinosaurus_cheloniformis* (96), *Achillesaurus_manazzonei* (100), *Ceratonykus_oculatus* (107), *Linhenykus_monodactylus* (108), *Beishanlong_grandis* (120), *Kinnareemimus_khonkaenensis* (123), *Compsognathus_longipes* (126), *Mirischia_asymmetrica* (129), *Ornitholestes_hermanni* (130), *Tugulusaurus_faciles* (133), *Philovenator_curriei* (157), *Pamparaptor_micros* (158), *Diuqin_lechiguanae* (160), *Kuru_kulla* (162), *Ypupiara_lopai* (166).

**all_unstable (group 29, 80 taxa)**: *Deinonychus_antirrhopus* (3), *Balaur_bondoc* (5), *Tsaagan_mangas* (6), *Tianyuraptor_ostromi* (8), *Graciliraptor_lujiatunensis* (13), *Hesperonychus_elizabethae* (14), *Pyroraptor_olympius* (15), *Rahonavis_ostromi* (16), *Buitreraptor_gonzalezorum* (17), *Neuquenraptor_argentinus*+*Unenlagia_*spp. (18), *Austroraptor_cabazai* (19), *Shanag_ashile* (20), *Atrociraptor_marshalli* (22), *Utahraptor_ostrommaysi* (23), *Adasaurus_mongoliensis* (24), *Achillobator_giganticus* (25), *Saurornitholestes_langstoni* (26), *Saurornithoides_mongoliensis* (27), *Zanabazar_junior* (28), *Xixiasaurus_henanensis* (29), *Sinornithoides_youngi* (31), *Mei_long* (32), *Aurornis_xui* (36), *Sinovenator_changii* (39), 'EK_troodontid_IGM_100/44' (40), *Archaeopteryx_lithographica* (43), *Confuciusornis_sanctus* (44), *Jeholornis_prima* (45), *Jixiangornis_orientalis* (46), *Yanornis_martini* (47), *Apsaravis_ukhaana* (48), *Yixianornis_grabaui* (49), *Sapeornis_chaoyangensis* (50), *Neuquenornis_volans* (51), *Patagopteryx_deferrariisi* (52), *Cathayornis_*spp. (53), *Concornis_lacustris* (54), *Gobipteryx_minuta* (55), *Vorona_berivotrensis* (56), *Songlingornis_linghensis* (57), *Pengornis_houi* (58), *Hesperornis_regalis* (59), *Baptornis_advenus* (60), *Ichthyornis_dispar* (61), *Iaceornis_marshii* (62), *Limenavis_patagonica* (63), *Lithornis*_spp. (64), *Hongshanornis_longicresta* (65), *Liaoningornis_longidigitris* (66), *Crypturellus_undulatus* (67), *Gallus_gallus* (68), *Crax_pauxi* (69), *Anas_platyrhynchos* (70), *Chauna_torquata* (71), *Pedopenna_daohugouensis* (72), *Rinchenia_mongoliensis* (81), *Conchoraptor_gracilis* (82), *Avimimus_portentosus* (84), *Erlikosaurus_andrewsi* (88), *Suzhousaurus_megatherioides* (92), *Enigmosaurus_mongoliensis* (94), *Therizinosaurus_cheloniformis* (96), *Achillesaurus_manazzonei* (100), *Albinykus_baatar* (103), *Ceratonykus_oculatus* (107), *Linhenykus_monodactylus* (108), *Xixianykus_zhangi* (109), *Archaeornithomimus_asiaticus* (113), *Beishanlong_grandis* (120), *Kinnareemimus_khonkaenensis* (123), *Compsognathus_longipes* (126), *Mirischia_asymmetrica* (129), *Ornitholestes_hermanni* (130), *Tugulusaurus_faciles* (133), *Philovenator_curriei* (157), *Pamparaptor_micros* (158), *Diuqin_lechiguanae* (160), *Shri_devi* (161), *Kuru_kulla* (162), *Ypupiara_lopai* (166).

**unstable_within_replicates (group 30, 10 taxa)**: *Graciliraptor_lujiatunensis* (13), *Hesperonychus_elizabethae* (14), *Pyroraptor_olympius* (15), 'EK_troodontid_IGM_100/44' (40), *Limenavis_patagonica* (63), *Pedopenna_daohugouensis* (72), *Erlikosaurus_andrewsi* (88), *Mirischia_asymmetrica* (129), *Pamparaptor_micros* (158), *Diuqin_lechigunae* (160).

**unstable_among_replicates (group 31, 79 taxa)**: *Deinonychus_antirrhopus* (3), *Balaur_bond*oc (5), *Tsaagan_mangas* (6), *Tianyuraptor_ostromi* (8), *Graciliraptor_lujiatunensis* (13), *Hesperonychus_elizabethae* (14), *Pyroraptor_olympius* (15), *Rahonavis_ostromi* (16), *Buitreraptor_gonzalezorum* (17), *Neuquenraptor_argentinus*+*Unenlagia_*spp. (18), *Austroraptor_cabazai* (19), *Shanag_ashile* (20), *Atrociraptor_marshalli* (22), *Utahraptor_ostrommaysi* (23), *Adasaurus_mongoliensis* (24), *Achillobator_giganticus* (25), *Saurornitholestes_langstoni* (26), *Saurornithoides_mongoliensis* (27), *Zanabazar_junior* (28), *Xixiasaurus_henanensis* (29), *Sinornithoides_youngi* (31), *Mei_long* (32), *Aurornis_xui* (36), *Sinovenator_changii* (39), *Archaeopteryx_lithographica* (43), *Confuciusornis_sanctus* (44), *Jeholornis_prima* (45), *Jixiangornis_orientalis* (46), *Yanornis_martini* (47), *Apsaravis_ukhaana* (48), *Yixianornis_grabaui* (49), *Sapeornis_chaoyangensis* (50), *Neuquenornis_volans* (51), *Patagopteryx_deferrariisi* (52), *Cathayornis_*spp. (53), *Concornis_lacrustris* (54), *Gobipteryx_minuta* (55), *Vorona_berivotrensis* (56), *Songlingornis_linghensis* (57), *Pengornis_houi* (58), *Hesperornis_regalis* (59), *Baptornis_advenus* (60), *Ichthyornis_dispar* (61), *Iaceornis_marshii* (62), *Limenavis_patagonica* (63), *Lithornis_*spp. (64), *Hongshanornis_longicresta* (65), *Liaoningornis_longidigitris* (66), *Crypturellus_undulatus* (67), *Gallus_gallus* (68), *Crax_pauxi* (69), *Anas_platyrhynchos* (70), *Chauna_torquata* (71), *Pedopenna_daohugouensis* (72), *Rinchenia_mongoliensis* (81), *Conchoraptor_gracilis* (82), *Avimimus_portentosus* (84), *Erlikosaurus_andrewsi* (88), *Suzhousaurus_megatherioides* (92), *Enigmosaurus_mongoliensis* (94), *Therizinosaurus_cheloniformis* (96), *Achillesaurus_manazzonei* (100), *Albinykus_baatar* (103), *Ceratonykus_oculatus* (107), *Linhenykus_monodactylus* (108), *Xixianykus_zhangi* (109), *Archaeornithomimus_asiaticus* (113), *Beishanlong_grandis* (120), *Kinnareemimus_khonkaenensis* (123), *Compsognathus_longipes* (126), *Mirischia_asymmetrica* (129), *Ornitholestes_hermanni* (130), *Tugulusaurus_faciles* (133), *Philovenator_curriei* (157), *Pamparaptor_micros* (158), *Diuqin_lechiguanae* (160), *Shri_devi* (161), *Kuru_kulla* (162), *Ypupiara_lopai* (166).
